# Phenotype and multi-omics comparison of *Staphylococcus* and *Streptococcus* uncovers pathogenic traits and predicts zoonotic potential

**DOI:** 10.1186/s12864-021-07388-6

**Published:** 2021-02-04

**Authors:** Niels A. Zondervan, Vitor A. P. Martins dos Santos, Maria Suarez-Diez, Edoardo Saccenti

**Affiliations:** 1 Laboratory of Systems and Synthetic Biology, Wageningen University & Research, Stippeneng 4, 6708WE Wageningen, Netherlands; 2 LifeGlimmer GmBH, Markelstraße 38, 12163, Berlin, Germany

**Keywords:** Staphylococcus, Streptococcus, Multi-omics, Comparison, Pathogenic, Traits, Prediction, Phenotype, Host-trophism, Zoonotic

## Abstract

**Background:**

*Staphylococcus* and *Streptococcus* species can cause many different diseases, ranging from mild skin infections to life-threatening necrotizing fasciitis. Both genera consist of commensal species that colonize the skin and nose of humans and animals, and of which some can display a pathogenic phenotype.

**Results:**

We compared 235 *Staphylococcus* and 315 *Streptococcus* genomes based on their protein domain content. We show the relationships between protein persistence and essentiality by integrating essentiality predictions from two metabolic models and essentiality measurements from six large-scale transposon mutagenesis experiments. We identified clusters of strains within species based on proteins associated to similar biological processes. We built Random Forest classifiers that predicted the zoonotic potential. Furthermore, we identified shared attributes between of *Staphylococcus aureus* and *Streptococcus pyogenes* that allow them to cause necrotizing fasciitis.

**Conclusions:**

Differences observed in clustering of strains based on functional groups of proteins correlate with phenotypes such as host tropism, capability to infect multiple hosts and drug resistance. Our method provides a solid basis towards large-scale prediction of phenotypes based on genomic information.

**Supplementary Information:**

The online version contains supplementary material available at 10.1186/s12864-021-07388-6.

## Background

Species from the genera *Staphylococcus* and *Streptococcus* are mostly commensals that live as part of the microbiota of various animals and humans [1]. Some of them are opportunistic pathogens, displaying a pathogenic phenotype when the immune system of the host is compromised or the epithelial barrier is damaged [2–5].

Few comparative genomic studies have been performed to analyse the evolution and the pathogenesis of *Staphylococcus* and *Streptococcus* species: the comparisons of the genomes of 11 *Staphylococcus* species determined that horizontal gene transfer of virulence factors is an important factor in adaptation of *S. aureus* to humans [6]; another study showed that protein domain based metabolic diversity among *Streptococcus* species could be used to identify differences in the metabolism of the highly pathogenic serotype 2 *S. suis* compared to other *Streptococci* [7]. Another study confirmed these results and showed that metabolic capability predicted using genome scale models (GEMs) could be used to identify *Streptococcus* strain specific biomarkers and metabolic determinants of virulence [8].

Protein domains and protein-domain architectures have been shown to be a fast and efficient method to define groups of functionally equivalent proteins that were used for comparative genomic studies [9, 10], including *Staphylococcus* and *Streptococcus* [11–13]. However, at the best of our knowledge, no work exists focusing on similarities and differences within and between *Staphylococcus* and *Streptococcus* genomes.

In this study we performed a comparative analysis of 235 and 315 fully sequenced *Staphylococci* and *Streptococci* genomes by annotating their proteins based on their domain content. We integrated this protein annotation with genome-scale metabolic-modelling predictions, transcriptomic and transposon-mutagenesis data sets to study gene essentiality and persistence. All annotation used in this paper as well as GO information is based on genomics annotation from databases based mainly on bacterial genomics studies. In this paper we compare within and between *Staphylococcus* and *Streptococcus* species with the objective to identify both difference and similarities in genomic properties as well as in specific combinations of genes that give rise to pathogenic phenotypes. We compared the clustering of *Staphylococcus* and *Streptococcus* genomes based on proteins selected using on Gene Ontology (GO) terms associated with clinical phenotypes such drug resistance, pathogenesis, and tissue and host tropism. Furthermore, we used the functional grouping of proteins to predict zoonotic potential of *S. suis* and *S. agalactiae*, that is their ability to infect multiple hosts including humans. Finally, we compared *S. aureus* and *S. pyogenes* to identify the genomic basis for their shared ability to cause severe bacterial infections like necrotizing fasciitis. Our results are compared throughout the paper with findings from literature.

## Results

### Pan- and core genome analysis

**Fig. 1:**
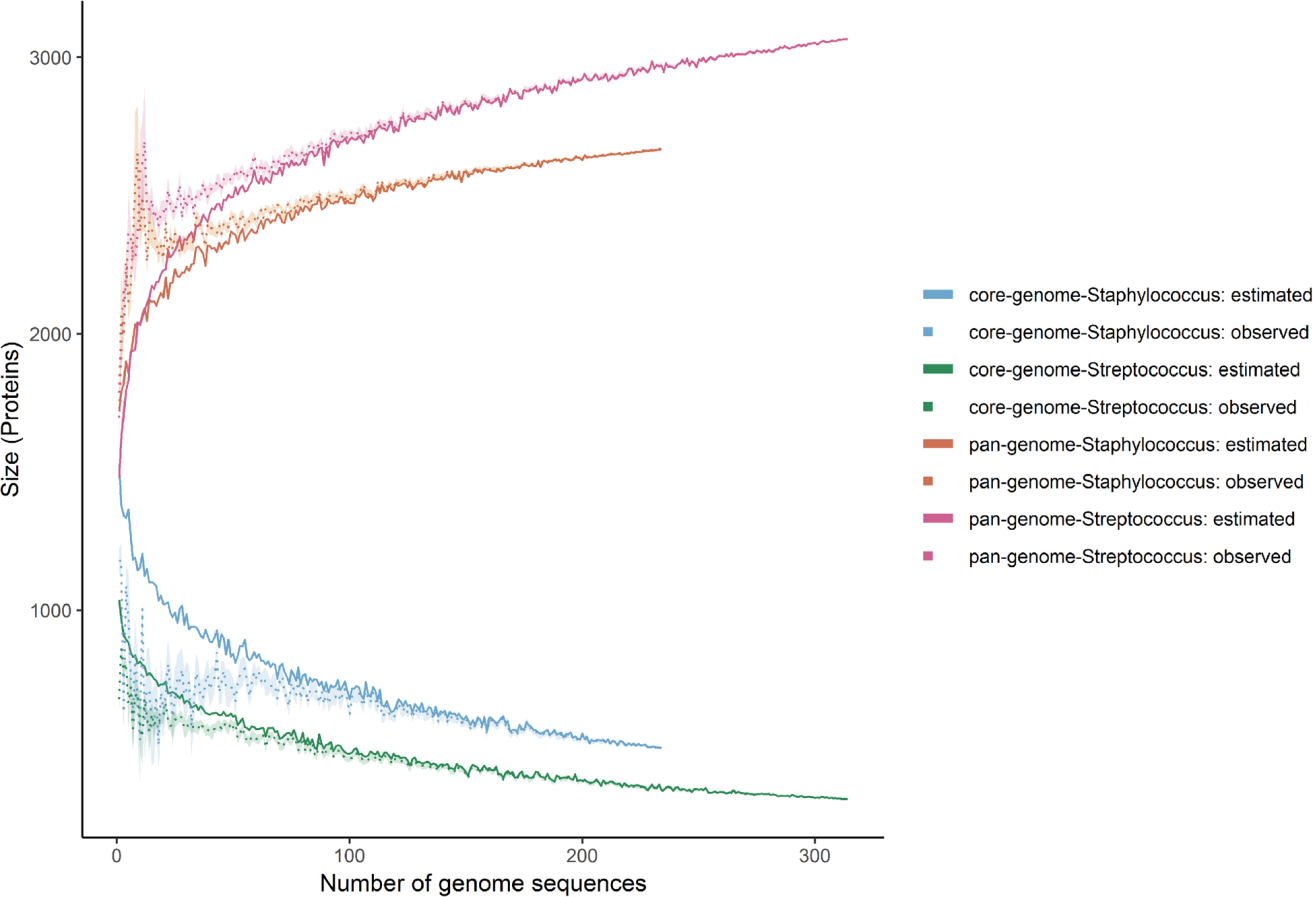
Mean observed and estimated size of the pan- and core genome. The shadowed area shows variation over 10 times sampling

The size of the pan- and core genomes of *Staphylococcus* and *Streptococcus* was determined based on protein domain content (Fig. 1). The pangenome contains all proteins present in the analysed genomes. The core genome contains only proteins that are present in all genomes and represents their genomic essence [14]. The ratio of the sizes of the core- and pan genome are 0.22 (557/2563) for *Staphylococcus* and 0.17 (458/2725) for *Streptococcus*.

A Heaps’ regression model was used to estimate the closedness of the pangenome [15]. The closedness of the pangenome represents how much the addition of more genome sequences is expected to increase the number of proteins in the pangenome. For both *Staphylococcus* (α = 1.10 ± 0.02) and *Streptococcus* (α = 1.12 ± 0.01) the pangenome was found to be closed (*i.e* few new genes are added as news strains are discovered/sequenced). Additional plots of the estimated pan- and core genomes size and the Heaps’ regression model can be found in supplementary material (see Additional file 5).

### Protein persistence and essentiality

**Fig. 2:**
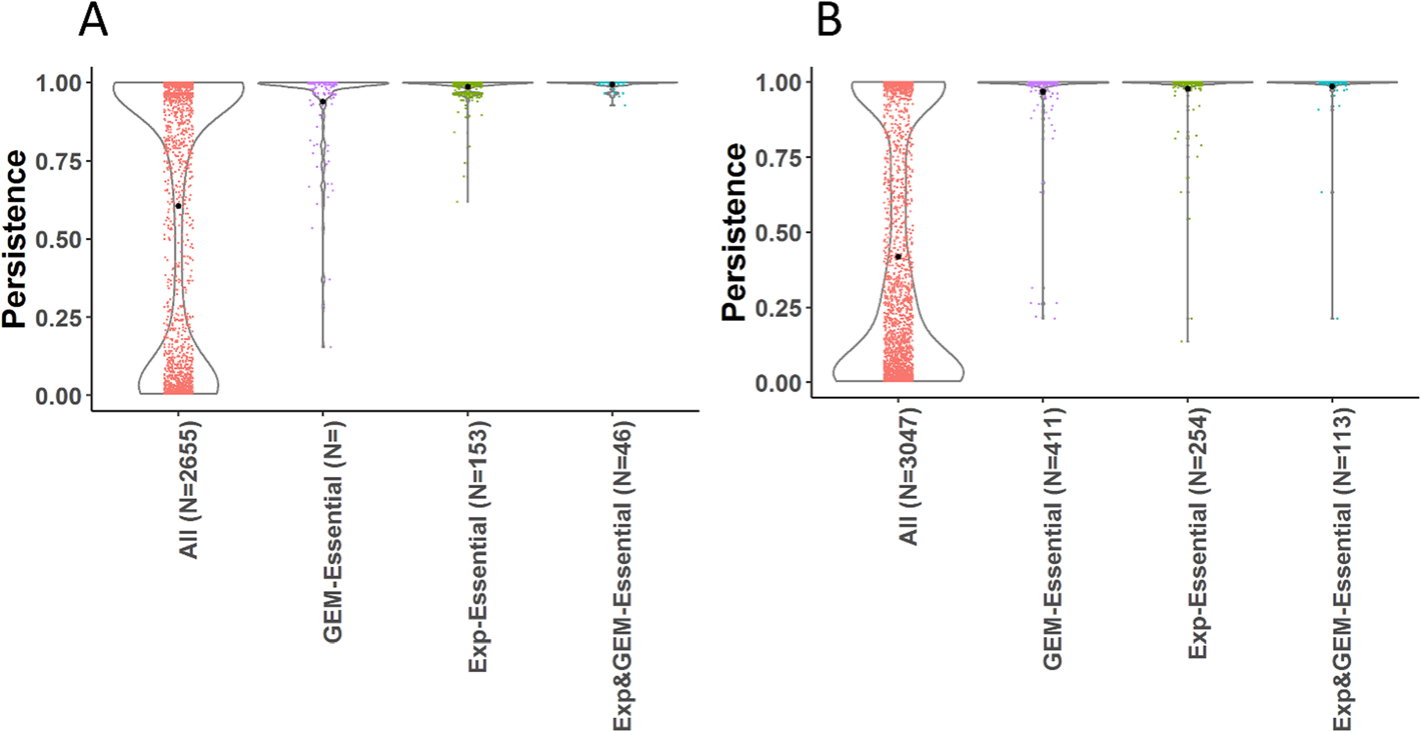
Protein persistence. **a** Staphylococcus, **b** Streptococcus. Group labels: All = all proteins, GEM = in silico predicted to be essential using a Genome Scale Metabolic model, Exp-Essential = experimentally determined to be essential. Combined group strains

Persistence of proteins over all *Staphylococcus* and all *Streptococcus* species was calculated. Protein persistence data was combined with model predictions of essentiality and experimentally determined essentiality data.Experimentally determined essentiality (labelled as EXP) is available for growth on rich media resembling in vivo conditions. GEMs predictions were made using minimal media conditions for all combinations of carbon, nitrogen, sulphur, and phosphorus sources. Simulations on rich media conditions were therefore indirectly performed since all rich media compounds are present in the models as exchange reactions and all combinations of these exchange reactions functioning as carbon, nitrogen, sulphur and phosphor sources were tested for essentiality. We used GEM to predict gene essentiality for *Staphylococcus aureus* NTCTC 8325 and *Streptococcus pyogenes* M49*.* The total number of medium combinations based on C, N, S, P sources was 12,432 for *Staphylococcus* and 714 for *Streptococcus*. The number of tested conditions for Staphylococcus is much larger than the Streptococcus model since the Staphylococcus model can use all amino acids as alternative nitrogen source through deamination, greatly increasing the number of minimal media combinations. The Staphylococcus model can use all amino acids as alternative nitrogen source through deamination, greatly increasing the number of minimal media combinations Protein persistence, in silico predictions of essential and in vitro essentiality data for *Staphylococcus* and *Streptococcus* were integrated based on their associated locus tags. Both GEM based and experimentally determined essentiality correlated with a high persistence, while essentiality by both criteria is associated with an even higher persistence (see Table 1 and Fig. 2). Proteins experimentally determined or GEM predicted to be essentiality are significantly different from the average protein persistence (Student’s *t*-test, *p*-value = 5 × 10^− 14^) for both *Staphylococcus* and *Streptococcus*.

**Table 1:** Persistence of *Staphylococcus* (Staph.) and *Streptococcus* (Strep.) for all proteins, proteins associated to Genome Metabolic model (GEM) essential genes and experimentally (EXP) determined essential genes

Group	Avg persistence Staph.	Avg persistence Strep.
All	0.60 ± 0.44 (*N* = 2655)	0.42 ± 0.42 (*N* = 3047)
GEM-essential	0.94 ± 0.14 (*N* = 153)	0.98 ± 0.09 (*N* = 225)
Exp-Essential	0.97 ± 0.03 (*N* = 411)	0.97 ± 0.12 (*N* = 254)
EXP&GEM-Essential	0.94 ± 0.01 (*N* = 46)	0.98 ± 0.00 (*N* = 113)

### Variability of gene expression and gene essentiality

**Fig. 3:**
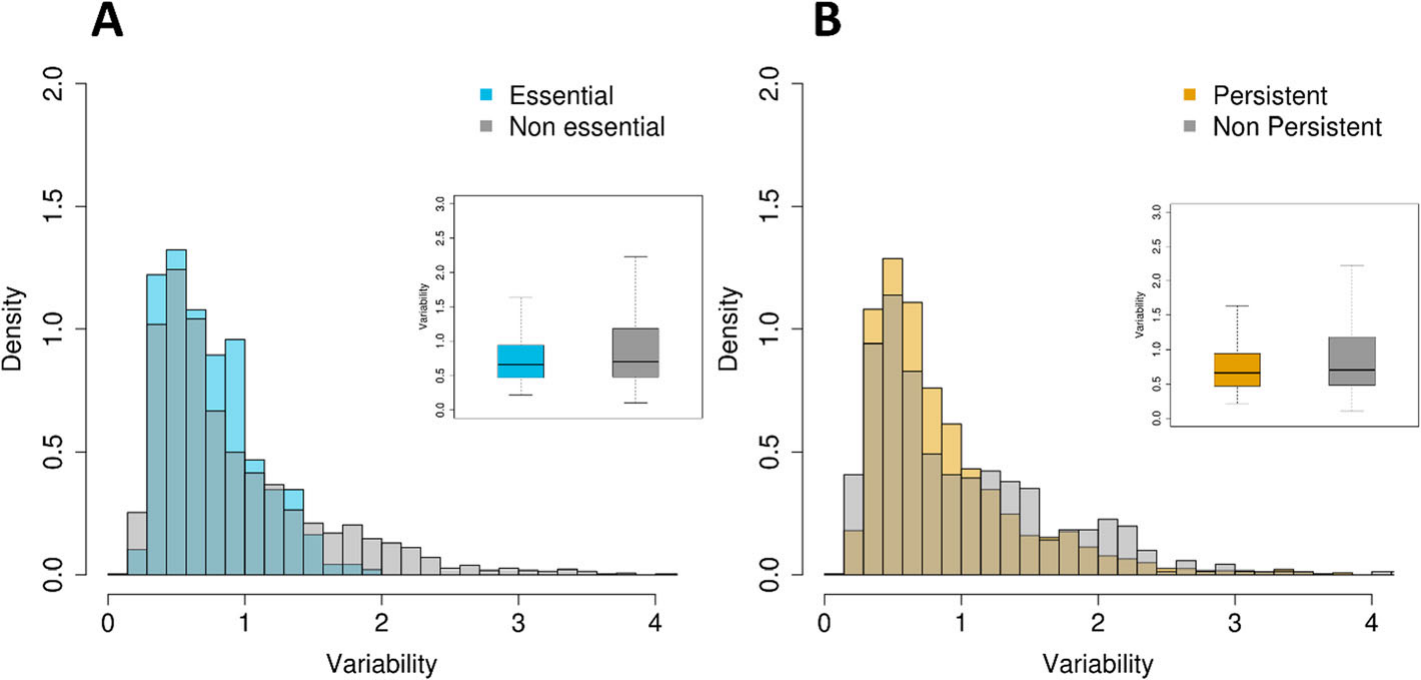
**a** Transcriptional variability of essential and non-essential genes in *Staphylococcus*. Box plots show Variability values for both groups. Difference between mean values is significant (*p*-val < 0.01). **b** Transcriptional variability of persistent and non-persistent genes (genes with persistence lower or higher than 0.95, respectively). Box plots show Variability values for both groups. Difference between mean values is significant (*p*-val < 0.01)

Essentiality and domain persistence information for *Staphylococcus* was combined with the variability of transcription (measured by log_2_ fold changes). The variability in expression for experimentally determined essential and non-essential genes as well as for persistent and non-persistent genes were compared (Fig. 3). The fold change transcription levels of experimentally determined essential genes are significantly less variable than the transcription levels of non-essential genes (Student’s *t-*test, *p*-value = 5 × 10^− 14^) as well as for persistent genes (Student’s *t*-test, *p*-value = 0.000124)).

### Functional analysis of pathogenesis and pathogenicity

For this analysis, we filtered proteins from *Staphylococcus* and *Streptococcus* on their association to 17 genome ontology (GO) biological process terms associated to pathogenesis and pathogenicity but cannot make predictions for rich media conditions were not all components are known or incorporated in the model. Filtering included all proteins associated to either the 17 main GO terms or any of their descendent terms. For all GO terms, proteins were found in both *Staphylococcus* and *Streptococcus* (Table 2). The ratio of proteins per GO function to the total number of proteins is similar for *Staphylococcus* and *Streptococcus* except for the group ‘Biological adhesions’ which has a larger fraction of proteins associated in *Streptococcus* than in *Staphylococcus.*

**Table 2:** Number of proteins in the pangenome of *Staphylococcus* and the pangenome of *Streptococcus* per GO term. Root ontology terms, terms without a parent, are marked in their description with an asterixis (*). GO terms are order as such that descendent GO terms are shown below their parent

Filter	Description	Staph	Strep
	All proteins	2655	3047
GO:0008150	Biological process	1974	2222
GO:0008152	*Metabolic process	1661	1871
GO:0017144	Drug metabolic process	59	77
GO:0042493	Response to drug	56	60
GO:0023052	*Signalling	217	280
GO:0065007	*Biological regulation	823	929
GO:0022610	*Biological adhesion	70	147
GO:0044406	Adhesion of symbiont to host	2	3
GO:0051704	Multi-organism process	348	456
GO:0044419	Inter species interaction between organisms	210	309
GO:0042710	Biofilm formation	9	10
GO:0098743	Cell aggregation	7	10
GO:0044403	Symbiont process	150	202
GO:0009372	Quorum sensing	7	7
GO:0035821	Modification of morphology or physiology of other organism	45	74
GO:0009405	Pathogenesis	65	115

Functional trees, PCA and t-SNE plots were used to compare the (dis-)similarity in clustering of the genomes based on functional groups of proteins compared to clustering based on all proteins. Dissimilarity was calculated using the Euclidean distances of genomes in the functional trees and by scaling these distances to values between 0 and 1 to make them comparable.

Functional trees, PCA plots and t-SNE plots for *Staphylococcus* can be found in supplementary material [Additional files 6, 7 and 8]. Functional trees, PCA plots and t-SNE pots for *Streptococcus* can be found in supplementary material (Additional files 9, 10 and 11). PCA plots and t-SNE plots for *Staphylococcus* and *Streptococcus* species combined can be found in supplementary material (Additional files 12 and 13).

Heatmaps were used to investigate which proteins are absent for each species. Each of these analyses and visualization methods has their own strength and weaknesses in showing the differences in clustering. In the following we highlight some of the differences in clustering of *Staphylococcus* and *Streptococcus* genomes based on proteins annotated per GO term as compared to clustering based on all proteins.

### Correlation between GO functional groups of proteins

**Fig. 4:**
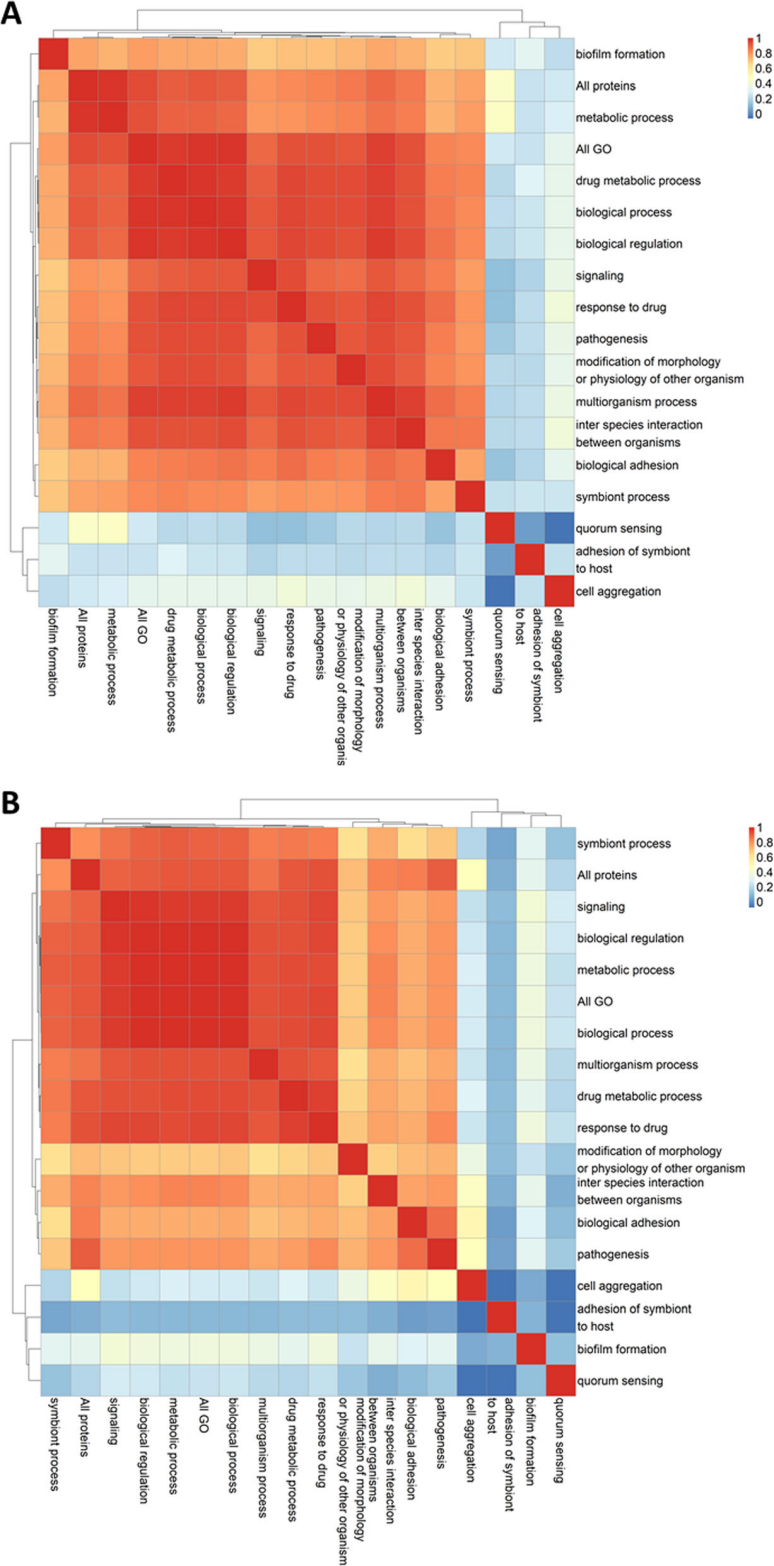
**a** Heatmaps of the correlation between *Staphylococcus* functional trees, **b** Heatmaps of the correlation between *Streptococcus* functional trees

We calculated the correlation between functional trees in order to compare the similarity in clustering per GO functional group of proteins (Fig. 4a-b). The correlation between functional trees is higher for children and parent GO terms as well as for GO terms with similar functions such as ‘drug metabolic process ‘and ‘response to drug’. In general, we see that functional trees based on fewer proteins have a lower correlation than functional trees based on many proteins. These results were expected since fewer proteins means less information to separate strains resulting in merging of branches in the tree. An interesting exception to this rule is the ‘symbiont process’ functional tree which has the lowest correlation with other functional trees for *Staphylococcus* even though there is a high number of proteins associated to this GO term.

There are some notable differences when comparing the correlation between functional trees for *Staphylococcus* and *Streptococcus.* For *Staphylococcus*, the ‘pathogenesis’ clusters together with the functional tree ‘modification of morphology or physiology of other organisms’. For *Streptococcus,* the functional tree of ‘pathogenesis’ clusters together with the functional tree of ‘biological adhesion’*.* Many ‘modification of host morphology’ proteins in *Staphylococcus* are also associated to the GO term ‘pathogenesis’ while many ‘biological adhesion’ proteins in *Streptococcus* are associated to the GO term ‘pathogenesis’. These results could indicate that modification of host morphology is important for the pathology of *Staphylococcus* strains while biological adhesion is more important for the pathology of *Streptococcus.*

### Horizontal gene transfer of proteins related to pathogenesis

**Fig. 5:**
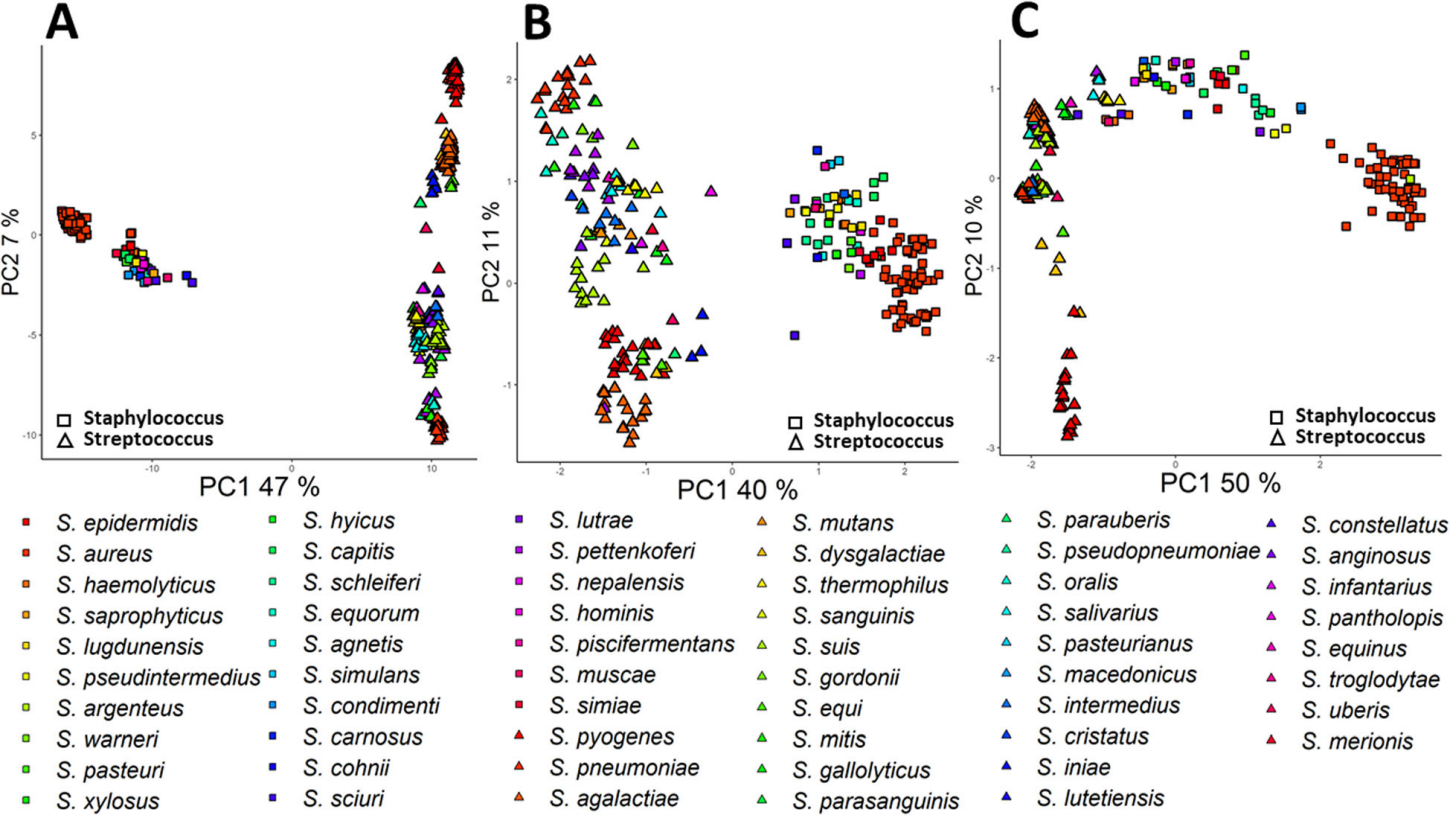
**a** PCA based on all proteins in *Staphylococcus* and *Streptococcus*. **b** PCA based on proteins association to ‘response to drug’ (GO:0042493). **c** PCA based on proteins associated to ‘pathogenesis’ (GO:009405). Fraction of variance explained by each PC is indicated in the axis

The PCA plot based on all proteins combining *Staphylococcus* and *Streptococcus* genomes supplementary material (Additional files 12 and 13), shows genomes of the same species to cluster together as we would expect (Fig. 5a). The PCA plot based on presence/absence of proteins involved in Response to drug (GO:0042493) shows genomes are not always separated on the species level, however, there is a clear separation between *Staphylococcus* and *Streptococcus* genomes (Fig. 5b). However, both in the PCA (Fig. 5c) and in t-SNE plots based on proteins associated to the GO term ‘Pathogenesis’ proteins, *Staphylococcus* and *Streptococcus* species cluster together.

Analysis of the presence and absence of proteins associated to ‘pathogenesis’ reveals that *Staphylococcus sciuri* GCA:002072755 and *Staphylococcus haemolytic us* GCA:001611955 only contain one pathogenesis protein (PF04647) that is not present in any *Streptococcus* strain. This protein, PF04647 ArgB, is part of a quorum sensing system. Also *S. saprophyticus* GCA:002209265 only contains one protein not present in any *Streptococcus* strain. This protein, PF05480, is a haemolytic protein unique to *Staphylococcus.* Among *Streptococci, S. parauberis* six *S. iniae* and seven *S. thermophilus* strains lack any pathogenesis protein that separates them from *Staphylococcus.*

### Domain shuffling of pathogenic proteins

The *Staphylococcus* pangenome contains 52 domains present in 65 proteins associated to the GO term ‘pathogenesis’ while the *Streptococcus* pangenome contains 88 domains present in 118 proteins associated to the GO term ‘pathogenesis’. 20% of pathogenic proteins in *Staphylococcus* and 25% of the pathogenic proteins in *Streptococcus* consist of a few pathogenesis associated domains combined with domains not directly associated to pathogenesis. This implies that domain shuffling might be an important evolutionary factor for these pathogens. In *Staphylococcus* 46% (30/65) and in *Streptococcus* 72% (85/118) of the pathogenesis associated proteins contain multiple domains. This percentage is much higher than the average percentage of multi-domain proteins of 8.9 and 9.2% for *Staphylococcus* and *Streptococcus* respectively. It could be argued that proteins involved in pathogenesis would more often require multiple domains since many of them are cell-wall associated, secreted or contain multiple domains to facilitate interaction between host and the pathogen. The importance of cell wall associated proteins is reflected by the high percentage of 40% of pathogenesis proteins in *Staphylococcus* and 66% *Streptococcus* that contain LPXTG cell-wall anchor domain PF00746. The importance of this domain for pathogenesis was shown in a *S. aureus* mutant with a knockout of *srtA* coding for a class A sortase, which is required for secretion of proteins containing the LPXTG motif. This mutant was unable to form abscess lesions in organ tissues or cause lethal bacteraemia when inoculated in the blood stream of mice [16].

### *Staphylococcus aureus* multi-drug resistance

We investigated the clustering of *S. aureus* genomes in the functional tree associated to the terms “response to drug”. We selected the genome of *S. aureus sub species aureus MRSA 252* (GCA:000011505)*,* which is known to be a multiple drug resistant strain [17]. Next, we searched literature for information about drug resistance for eight genomes that cluster together with this strain in the functional tree response to drug. For seven of these strains (JH1, JH9, Mu50, Mu3, T0131, 04–20,981), evidence was found for these strains to be multi-drug resistant as well as identifying two pathogenicity islands as the cause of their resistance [18–22]. For the last genome (GCA:001640885), no literature or other information could be retrieved. This genome has exactly the same proteins associated to response to drug as the seven strains for which multi drug resistance was reported in literature. Therefore, we can speculate that this strain may have the same multi drug resistance phenotype.

### *Streptococcus suis* pathogenesis zoonotic potential

Large differences in clustering were observed for *S. suis* genomes in the functional trees relating to ‘biological adhesion’, ‘modification of morphology or physiology of other organism’ and ‘pathogenesis’, supplementary material (Additional file 9). *S. suis* genomes form two groups in the functional tree of biological adhesion, and three groups in the functional tree of pathogenesis and ‘modification of morphology or physiology of other organism’.

**Fig. 6:**
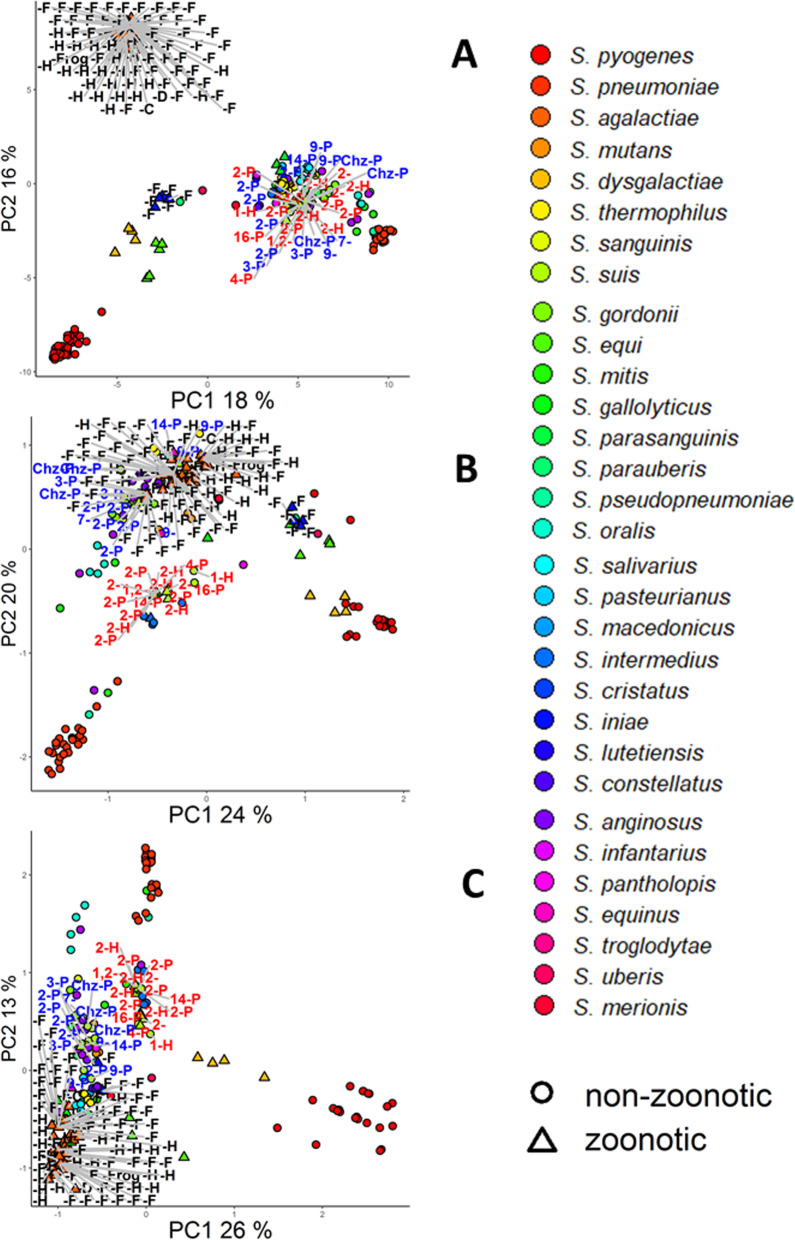
PCA plot of *Streptococcus* strains based on all proteins (**a**), proteins filtered on ‘modification of morphology or physiology of other organism’ (**b**) and proteins filtered on ‘pathogenesis’ (**c**). *S. suis* serotypes are shown in the label, genomes from species mentioned in literature as having zoonotic capabilities are marked with a triangle and the isolation host is marked in the label with D = dog, F = fish, H = human, P = pig, T = toad. Genomes predicted in this study to have zoonotic potential are coloured red while strains in the cluster predicted not to have zoonotic potential are coloured blue. Fraction of variance explained by each PC is indicated in the axis

Similarly, different groups can be distinguished in the PCA plot based on these three functional groups, as shown in Fig. 6a-c*.* We included information from literature on zoonotic species, namely *S. inae, S. agalactiae, S. dysgalactiae S. iniae* and*, S. equi zooepidemicus* and *S. suis* serotype 2 strains and serotype information and host isolation information for *S. suis* and *S. agalactiae* strains in the labels of Fig. 6a-c*.* Two *S. suis* clusters can be distinguished in the PCA score plot based on proteins related to ‘modification of morphology or physiology of other organism’ (Fig. 6b) and the PCA based on ‘pathogenesis’ proteins (Fig. 6c): the first cluster contains 7 out of the 12 serotype 2 strains, as well as serotype 1, 1,2, 4, 16, while the second cluster contains 5 serotype 2 strains as well as strains with serotype 3, 7, 9 14 and Chz which were all isolated from pigs. The first group contains *S. suis* zoonotic strains of which some are isolated from pig and some from humans. The second group contains are non-zoonotic strains all isolated from pigs.

### *Streptococcus agalactiae* zoonotic potential

**Fig. 7:**
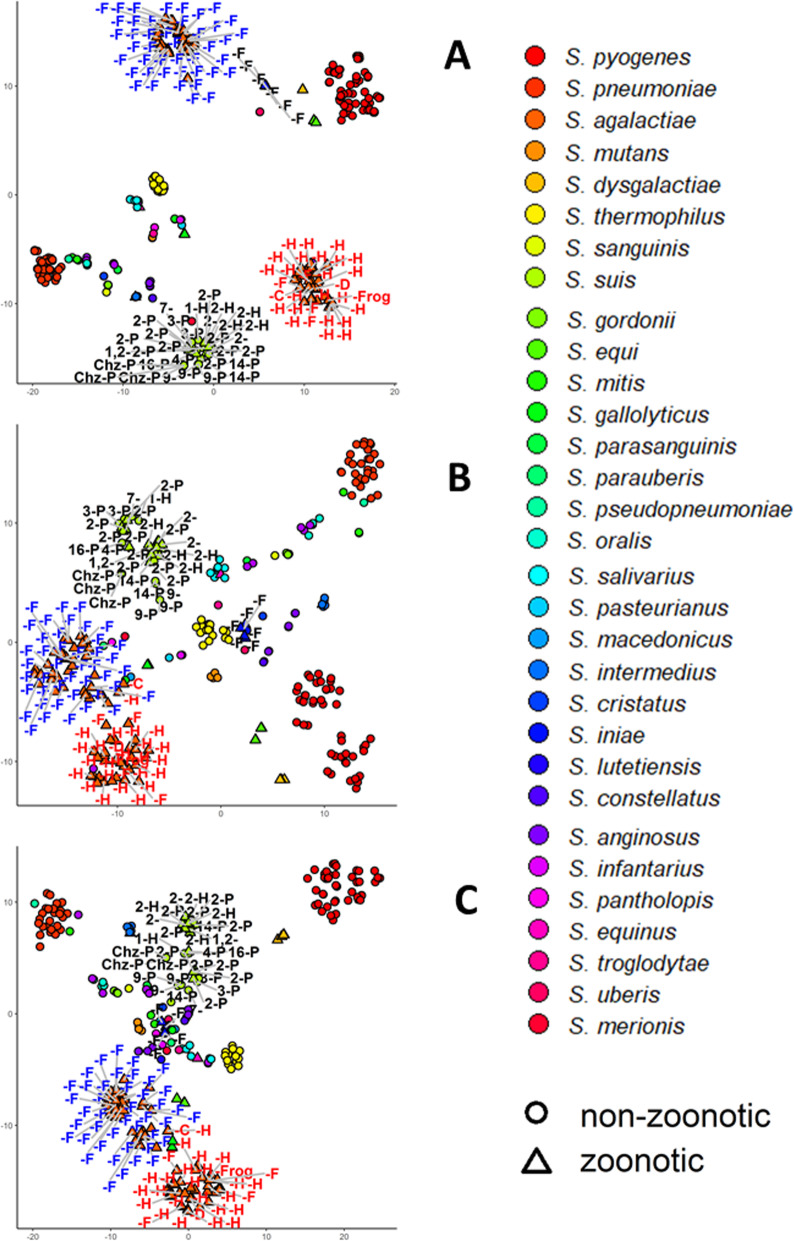
t-SNE plots of Streptococcus strains based on proteins all proteins (**a**), proteins filtered on ‘biological adhesion’ (**b**) and ‘pathogenesis’ (**c**). t-SNE is a technique for dimensional reduction and visualization, so that similar objects appear as nearby objects in the two-dimensional plots here presented. *S. suis* serotypes are shown in the label, genomes from species mentioned in literature as having zoonotic capabilities are marked with a triangle and the isolation host is marked in the label with D = dog, F = fish, H = human, P = pig, T = toad. Genomes predicted in this study to be part of the zoonotic potential cluster are coloured red while strains in the cluster predicted not to have zoonotic potential are coloured blue. Fraction of variance explained by each PC is indicated in the axis

Like *S. suis, S. agalactiae* forms two clusters when clustering on GO biological functional groups of proteins. Based on their isolation host, we can see that a cluster contains strains that are zoonotic while the other contains strains that are non-zoonotic. These two groups of *S. agalactiae* strains are better separated when using t-*t*-SNE plots based on all proteins and proteins involved in biological adhesion and pathogenesis (Fig. 7a-c) suggesting the existence of few proteins that are present in every genome in each group.

### Identification of proteins that confer zoonotic potential

**Fig. 8:**
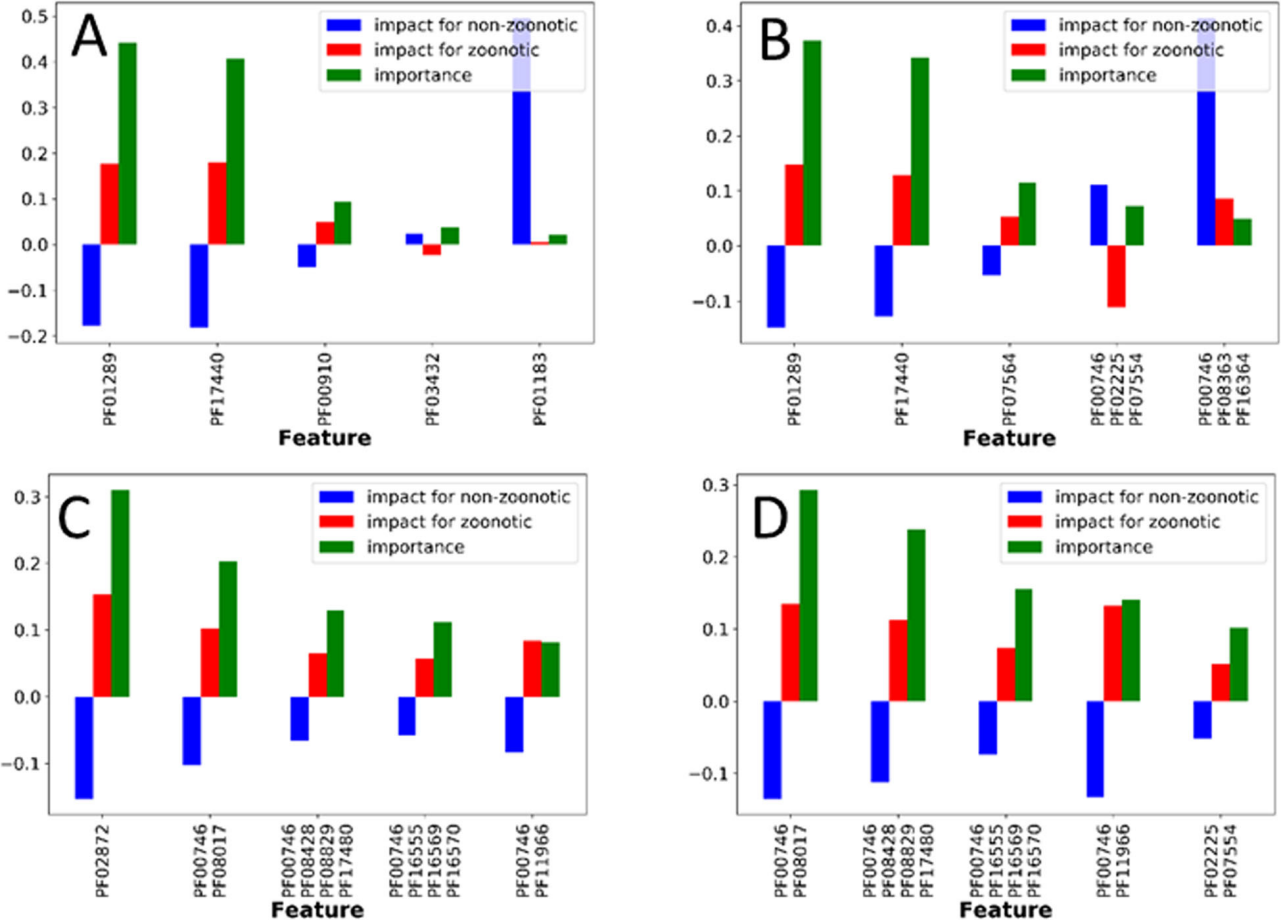
Protein feature contribution to predict the class ‘non-zoonotic’ and ‘zoonotic’ as well as the overall importance of the protein feature for classification. **a** The five most important ‘modification of morphology or physiology of other organism’ proteins used to classify *S. suis*. **b** The five most important ‘pathogenesis’ proteins used to classify *S. suis*. **c** The five most important ‘biological adhesion’ proteins used to classify *S. agalactiae*. **d** The five most important ‘pathogenesis’ proteins used to classify *S. agalactiae*

We used Random Forest, a machine learning approach, to investigate the association between genome content and phenotype using 75% of the data for training and 25% of the data for validation. Specifically, presence/absence of proteins filtered on association to GO Biological functions involved in pathogenesis to predict zoonotic potential *S. suis* and *S. agalactiae*, and we investigated which proteins are responsible for the zoonotic potential in these two species. We used functional groups of proteins that were shown to separate zoonotic and non-zoonotic strains for *S. suis* (Fig. 6b-c) and for *S. agalactiae* (Fig. 7b-c) to train a Random Forest classifiers. We investigated their overall importance for prediction as well as their contribution to predicting the class non-zoonotic, and the class zoonotic potential as shown in Fig. 8a-d. Where, the ‘Impact’ measure indicates the relevance of a protein of the prediction of given class. The ‘importance’ shows the proteins overall importance for the random forest classifier. Random Forest classifiers as well as the optimal hyper parameters can be found in (see Additional file 14).

The protein domain content of the five most important proteins for *S. suis* classification based on ‘modification of morphology or physiology of other organism’ proteins are: 1) PF01289 a thiol-activated cytolysin, 2) PF17440 thiol-activated cytolysin beta sandwich domain, 3) PF00910 replication initiation protein involved in viral RNA duplication 3) PF00078;PF08388;PF13655 group II intron reverse transcriptase/maturase, 4) PF03432 a relaxase involved in transfer of plasmids, 5) PF00665 Prokaryotic N-terminal methylation motif often found in pilins and other proteins involved in secretion (Fig. 8a).The most important proteins for *S. suis* classification based on ‘pathogenesis’ proteins are 1) PF01289 thiol-activated cytolysin, 2) PF17440 a thiol-activated cytolysin beta sandwich domain, 3) PF07564 hypothetical protein containing a domain of unknown function, 4) PF00092; PF00746 chemotaxin protein 5) PF00746;PF08363;PF16364 a glucan binding protein (Fig. 8b).

The *S. suis* classifiers based on ‘modification of morphology or physiology of other organism’ proteins as well the classifier based on ‘pathogenesis’ proteins, predict *S. suis* zoonotic potential with 100% accuracy solely based on the presence of either PF01289, a thiol-activated cytolysin or PF17440, a thiol-activated cytolysin beta sandwich Fig. 8a-b).

The most important features for *S. agalactiae* classification based on ‘biological adhesion’ proteins are 1) PF02872 a 5′-nucleotidase-C 2) PF00746;PF08017 Fibrinogen binding protein A, 3) PF00746; PF8428; PF08829; PF174802 surface protein Rib and 4) PF00746;PF16555; PF16569; PF16570 pilus complex 5) PF00746;PF11966 a cell wall anchored linked to a ySIRK signal domain (Fig. 8c). The most important features for *S. agalactiae* classification based on ´pathogenesis’ proteins are 1) PF00746;PF08017 Fibrinogen binding protein, 2) PF00746;PF8428;PF08829;PF17480 2) surface protein Rib and 3) PF00746;PF16555;PF16569;PF16570 pilus complex 4) PF00746;PF11966 a cell wall anchored linked to a ySIRK signal domain 5) PF02225;PF07554, a serine protease (Fig. 8d).

For *S. agalactiae* classification, 5′-nucleotidase-C can predict training data with 100% accuracy and test data with 94% accuracy.

### Virulence factors of necrotising fasciitis

We compared *Staphylococcus aureus* and *Streptococcus pyogenes* since both are a major cause of (monomicrobial) necrotizing fasciitis. Both *S. aureus* [23–25] and *S. pyogenes* [26, 27] fully lyse red blood cells, induce toxic shock syndrome as well as bind and invade epithelial host cells. Based on their GO term association *S. aureus* has 21 proteins associated to pathogenesis that occur in nearly all *S. aureus* genomes and only rarely in any other *Staphylococcus* specie (Table 3). Some of these pathogenesis proteins can directly be linked to pathogenesis proteins reported in literature for *S. aureus* [31] and for *S. pyogenes* [32]. An exact match with proteins reported in literature is however not always possible due to differences in annotation. When considering virulence factors that are not unique to *S. aureus* or *S. pyogenes* the number of virulence factors is about 1.5 times as many as reported in literature [33].

**Table 3:** Proteins associated to *S. aureus* Pathogenesis (GO: GO:0009405). Domains that are shared between *S. aureus* and *S. pyogenes* are underlined. Proteins that are shared have are written in bold

Protein	Description
PF00746;PF07501; PF17041	LPXTG cell wall anchor; G5 domain, suggested adhesion, in peptide that cleaves IgA; E domain, rod like structure
PF00746; PF17210	LPXTG cell wall anchor, SdrD B-like domain, involved in adhesion to nose squamous cells [28]
PF13545	Crp-like helix-turn-helix domain, possibly cAMP interaction
PF05543	Staphopain peptidase C47, secreted cysteine protease
PF14731	Staphopain proregion
PF03373	Octapeptide repeat, part of SpA virulence factor frequently used to type *S. aureus* strains [29]
PF07968	Haemolysin, part of the Leukocidin/Hemolysin toxin family
PF09199	Staphylococcal superantigen-like OB-fold domain, interact with IgA, inhibits the end stage of complement activation and IgA binding to Fc-α-R [30]
PF02216	SpAB protein domain, immunoglobin binding domain
**PF02876**	Staphylococcal/Streptococcal toxin, beta-grasp domain
PF11621	C3 binding domain 4 of IgG-bind protein SB
**PF01123**	Enterotoxin type B, supertoxin, involved in food poisoning, causing the immune system to release a large number of cytokines that lead to significant inflammation
PF03642	MAP domain, major histocompatibility complex class II analog
PF00746;PF01476	LPXTG cell wall anchor motif; LysM domain found in many receptors, peptidoglycan-binding protein [12]
**PF00746;PF02986**	LPXTG cell wall anchor motif; Fibronectin binding repeat, enables uptake by host cell
PF00746;PF05031	LPXTG cell wall anchor motif; Iron Transport-associated domain, heme and/or hemoprotein-binding
PF07564	Domain of Unknown Function (DUF1542), several proteins containing this domain are involved in antibiotic resistance and/or cell adhesion
PF09023	Staphostatin B inhibits the cysteine protease Staphopain B
PF02821	Streptokinase (SK) is a thrombolytic medication and enzyme, breaks down blood cloths
PF01468	GA module, GA modules may promote bacterial growth and virulence in mammalian hosts by scavenging albumin-bound nutrients and camouflaging the bacteria
PF07554;PF07564;PF08428	FIVAR domain, likely binds fibronectin or more specifically N-acetyl glucosamine, occurs in proteins involved in methicillin resistance; Domain of Unknown Function (DUF1542); Rib/alpha-like repeat. Occurs in some Rib, a thought to confer protective immunity. Occurs in some *Streptococcus* surface proteins. Extracellular matrix-binding protein Ebh

We looked at shared proteins as well as functional alternatives to find the molecular basis for necrotising fasciitis and we used the PFAM description of protein domains as well as description of proteins based on the locus tags associated to these proteins. We found that both *S. aureus* and *S. pyogenes* contain proteins involved in fibronectin binding, wound invasion, haemolysis, cell adhesion, IgA and IgG binding, multiple (super-)toxins as well as proteins involved in resisting phagocytosis and invading host cells (Table 3, Fig. 5). For example, PF01123 and PF02876 toxin β-grasp domain together form Enterotoxin type 2 which is important for causing the toxic shock [4, 34]. Enterotoxin type 2 antibodies are currently in clinical trials tested and have shown potential in treating necrotizing fasciitis [35].

*S. pyogenes* has 10 proteins that present in all *S. pyogenes* species and only occur separately in a few other *Streptococcus* strains (Table 4, Fig. 5). Five of these proteins are associated to the ability of *S. pyogenes* to bind to and break down fibrin in blood cloths [27]. Other proteins include toxin and enterotoxin, involved in over-activation of the immune response [38, 39], fibrin binding proteins, involved in adhesion and intracellular access of host cells, as well as proteases, involved in resistance to phagocytosis [40]. *S. pyogenes* has fewer proteins that are unique to this species compared to *S. aureus* since many other *Streptococcus* strains produce some of the pathogenic proteins present in *S. pyogenes* [41].

**Table 4:** Proteins associated to *S. pyogenes* Pathogenesis (GO: GO:0009405). Domains that are shared between *S. aureus* and *S. pyogenes* are underlined. Proteins that are shared are written in bold

Protein	Description
PF02821	Streptokinase, breaks down blood cloths
PF01640	Peptidase C10 family
**PF01123**	Enterotoxin type B, super antigen involved in food poisoning
**PF02876**	Staphylococcal/Streptococcal toxin, beta-grasp domain
PF03734	L,D-transpeptidase catalytic domain, peptidoglycan binding
PF00746; PF01391	LPXTG cell wall anchor motif; Collagen helix, rod like structure, coagulation-fibrinolytic binding in blood, Scl1 adhesin specifically recognizes the wound microenvironment [36]
PF00746; PF02370	LPXTG cell wall anchor motif; M protein repeat, binds IgA, major virulence factor involved in host cell invasion and resistance to phagocytosis [37]
PF00746; PF08017	LPXTG cell wall anchor motif; Fibronogen binding protein, members of this family include the fibrinogen receptor, FbsA which mediates platelet aggregation
**PF00746; PF02986**	LPXTG cell wall anchor motif; Fibronectin binding repeat, mediate adherence to host cells, enable the colonisation of wound tissue and blood clots
PF00092; PF00746; PF02986	Von Willebrand factor type A domain domains participate in numerous biological events (e.g. cell adhesion, migration, homing, pattern formation, and signal transduction); LPXTG cell wall anchor motif; Fibronectin binding repeat

Only three proteins associated to pathogenesis are shared between *S. aureus* and *S. pyogenes,* Enterotoxin B C-terminal domain (PF02876), Enterotoxin B N-terminal beta-grasp domain (PF01123) and Fibronectin binding protein (PF00746;PF02986)*.* Both fibronectin binding protein A (FnbpA) and B (FnbpB) are expressed during infection conditions and were shown to be complexly regulated by a large number of regulators such as sigma factors and two component systems by *Mader* et al. [42]. Among these proteins identified in our study are potential biomarkers. *FnbpA* was found not to be essential in KO studies [43], but was found to be essential in a rapid shotgun antisense RNA method to identify essential genes in *S. aureus* [44].. No essentiality information is available for FnbpB. *S. aureus* fibronectin binding protein A (FnbpA) is called fibronectin binding protein X (SfbX) in *S. pyogenes*. For *S. aureus,* FnbpA was found to be essential for entry in the host cells [45]. FnbpA has functional homologs in other species such as *S. epidermidis*, however all homologs lack the C-terminal multiple fibronectin binding repeats variants present in FnbpA, of which at least one high affinity binding repeat is needed for host cell uptake [16, 34, 46]. A SfbX knockout mutants was shown to be only minimally affect *S. pyogenes* ability to infect epithelial host cells [47]. Enterotoxin type B, is a super toxin involved in over-activation of the immune response and interferes with phagocytosis by supressing the generation of myeloid-derived suppressor cells [48–50].

## Discussion

The *Staphylococcus* and *Streptococcus* genera were compared on their genomic properties. Both genera have a similar ratio of their pan and core genome size. It should be considered that this analysis has been done with all fully assembled genomes data that were available at the time of the study. In our study we do not separate between pathogenic and non-pathogenic species since there are several ways to infect humans and animals. Instead, we use the underlying annotation of proteins marked as being involved in GO functions associated to pathogenesis to investigate patterns in pathogenesis. The choice not to define before-hand if species are pathogenic is deliberate since we recognize there are many forms of pathogenesis which depend on both species as well as the infection site as we discuss in the sections “Streptococcus suis pathogenesis zoonotic potential” and in the section “*Streptococcus agalactiae* zoonotic potential”. We do however recognize the selected population affect our results as can be seen for in the ratio of their pan and core genome size found in this study. The alpha value of 1.12 found in our study for *Streptococcus* is higher than the 0.87 values reported by Koehorst et al. [12]. This difference can be explained by the number of genomes analysed which was 314 in our study opposed to 60 in the study by Koehorst et al. Additionally, we allowed a maximum of one genome per species to be selected in our sampling approach to avoid population bias introduced by species with many sequenced genomes such as *S. aureus* which was not the case in the analysis of by Koehorst et al. [12].

Similar to what was found for *Pseudomonas* [9], gene expression variability of essential genes was found to be less than the expression variability of non-essential genes in both *S. aureus* and *S. pyogenes*.

Combination of experimentally determined essentiality and GEM based essentiality prediction were shown to be associated to a higher protein persistence than each of them individually. These results are to be expected since In vitro essentiality measurements are often only available for one condition, while GEM can easily be used to predict essentiality over multiple media conditions. Our GEM analysis predicted 153 *Staphylococcus aureus* genes to be essential in 90% of the minimal medium combination tested, while 163 genes were found to be essential for growth on rich medium and minimal medium. For *Streptococcus*, 196 genes were found to be essential in 90% of the minimal medium combinations tested, while no genes were found to be essential on rich medium. The *Streptococcus* model contains exchange reactions for all nutrients necessary for growth, meaning only the biomass reaction was found to be essential. Since we know from experimental results that there are several essential genes in *S. pyogenes*, we chose our method of testing all minimal medium compounds to best balance false positive and false negative results while keeping a unified method for our GEM essentiality analysis in both *S. aureus* and *S. pyogenes.* Similar to what has been experimentally observed and was shown by previous published GEM simulations [8], our simulations show that *Staphylococcus* can use amino acids as alternative carbon source for survival in the host [51]. A possible limitation of this approach is that GEM predictions can only be made for metabolic (and their associated) proteins. Although many highly persistent genes tend to be essential, not all are highly persistent. This indicated alternatives in essentiality exist [52]. Similarly, many non-essential genes do have a high persistence, indicating they might be essential for *Staphylococcus* or *Streptococcus* specific functions such as survival and growth in non-lab conditions such as those found in the host.

Differences in pathogenesis, essentiality as well as other properties such as drug resistance, arise from different selection pressures for individual species within genera [53]. For example, similar to what was found in this study, a recent study shows that although streptococcal virulence factors have no clear patterns among species groups, some virulence factors were shown to be congruous with the evolution of species groups [13]. Core genes together with accessory genes form a complex network that comprise the molecular basis of virulence in *Staphylococcus* and *Streptococcus* [54, 55]. Within some individual species, strong selective pressure exist as was shown for *S. aureus* MRSA resistant species where there is an interplay of two strong evolutionary selective pressures: 1) the host type and 2) the antibiotics used in treatment which varies between humans, pets and livestock [56, 57].

We compared the similarity and differences between *Staphylococcus* and *Streptococcus* based on the clustering of species in GO functional trees. Some notable differences where observed between *Staphylococcus* and *Streptoccus*. Many ‘modification of host morphology’ proteins in *Staphylococcus* are also associated to the GO term ‘pathogenesis’ while many ‘biological adhesion’ proteins in *Streptococcus* are associated to the GO term ‘pathogenesis’. These results could indicate that modification of host morphology is important for the pathology of *Staphylococcus* strains while biological adhesion is more important for the pathology of *Streptococcus.*

Next, we looked at which pathogenesis proteins separate *Staphylococcus* from *Streptococcus* species. Analysis of the presence and absence of proteins associated to ‘pathogenesis’ reveals that *Staphylococcus sciuri* GCA:002072755 and *Staphylococcus haemolytic us* GCA:001611955 only contain one pathogenesis protein (PF04647) that is not present in any *Streptococcus* strain. These results could indicate that horizontal gene transfer of pathogenic proteins occurred between *Staphylococcus* and *Streptococcus* or that they only carry pathogenesis proteins derived from a common ancestor.

Additionally, some pathogenic proteins only occur in one or a few genomes, indicating horizontal gene transfer from species outside the *Staphylococcus* and *Streptococcus* genus. Horizontal gene transfer is known to be a driving factor in the development of pathogenesis in *Staphylococcus* and *Streptococcus* [2, 6, 58–60]. For example, fibronectin binding domain PF02986 has been acquired by *Staphylococcus* and *Streptococcus* from an animal host, further spread among different *Streptococci* and *Staphylococci* through horizontal gene transfer, and further evolved through domain shuffling [41, 61].

Clustering of *Streptococcus* and *Staphylococcus* species based on different GO functional groups revealed sub cluster to be present for *S. suis* based on GO functional groups *‘modification of host morphorlogy’* and *‘pathogenesis’,* and revealed a sub cluster to be present for *S. agalactiae* based on GO functional groups *‘biological adhesion’* and *‘pathogenesis’.* What is more, these sub cluster coincides with the potential to infect multiple hosts*.* It is known that predominantly *S. suis* serotype 2 strains are associated to zoonotic potential [62, 63]. However, as we could see in Fig. 6a-c, serotype information is not able to separate zoonotic and non-zoonotic *S. suis* strains.

Based on their isolation host we can see that the first group are *S. suis* strains are zoonotic, while the second group are non-zoonotic strains. Furthermore, human infections with strains for all serotypes in the first cluster have been reported [64, 65]: these results show that these functional groups of proteins can be used to predict the *S. suis* zoonotic potential. Interestingly, the zoonotic group of *S. suis* strains clusters with the human and dog oral commensal *S. intermedius* which can cause meningitis through brain abscesses as well as liver abscesses and in some rare cases endocarditis [66]. Since *S. suis* and *S. intermedius* are distantly related, this clustering is specific for proteins with functions in modification of host morphology and pathogenesis.

The similarity of phenotypes such as causing meningitis and tropism for brain and liver, suggests these traits may be caused by ‘modification of morphology or physiology of other organism’ and ‘pathogenesis’ proteins, and suggest a causal relationship between the proteins associated to these GO terms and the observed phenotype.

Investigation of proteins required to predict *S. suis* zoonotic potential using a random forest classifier revealed PF01289 thiol-activated cytolysin, 2) PF17440 a thiol-activated cytolysin beta sandwich domain to be the two most important factors associated to zoonotic potential. In support of these findings, it was found that a *S. suis* cytolysin knockout mutant made the strain non-haemolytic and non-cytotoxic for cultured macrophage-like cells [67] while increased secretion of thiol activated cytolysins was shown to directly cause epithelial cell damage in humans, allowing *S. suis* to spread into deeper tissues [68]. Based on these studies it appears these two cytolysins are involved in formation of a pore-forming complex in cholesterol containing host membranes, which explains their importance for conferring zoonotic potential.

It has been suggested that *S. agalactiae* may have jumped from animals to humans in a certain moment of the evolution although it is still debatable if this zoonotic potential remains nowadays [69]. Here, we show that based on their genomic content *S. agalactiae* can be separated in two groups, one that is zoonotic and infects humans, fish and dog, and one group that only infects fish. This separation can be made based on all proteins, indicating zoonotic and non-zoonotic species are likely to have separated some time ago. In the *t*-SNE plots of biological adhesion and pathogenesis a third group can be seen of strains that infects mainly fish but also cow and human. This third cluster contains *S. agalactiae* strains that infect human most likely originate from this cluster and have further adapted to their human host by acquisition of proteins involved in biological adhesion and pathogenesis. The strains in this group appear to retain zoonotic potential since the cluster contains isolates from humans, fish and dog.

Investigation of proteins required to predict *S. agalactiae* zoonotic potential using a random forest classifier revealed multiple proteins to be important for classifying species as zoonotic. *S. agalactiae* 5′-nucleotidase-C is present in two proteins: Trifunctional nucleotide phospho-esterase protein YfkN precursor and Endonuclease YhcR precursor. Secreted nucleases play a role in evasion of the human innate immune response via destruction of extracellular traps and interference with phagocytosis signals [70]. Fibrinogen binding protein A allows *S. agalactiae* to attach to fibrinogen and to aggregate platelets [71]. Rib protein contains a Rib domain that confers protective immunity and an alpha C and alpha N protein domains involved in invasion and translocation along human epithelial cells according to their PFAM description. The pilus complex contains Pillin D1, Pillin B and Pillin D3 domains and contributes to the initial attachment and invasion of lung and cervical epithelial cells [71]. PF02225;PF07554 CspA serine protease breaks down three chemokines that attract and activate neutrophils [72].

In summary, all proteins important for classification of zoonotic potential appear to be causal to the zoonotic potential phenotype. Of these proteins, nucleosidases YfkN, YhcR and fibrinogen binding protein appear to be the most important factors for *S. agalactiae* zoonotic potential.

## Conclusions

In this study we dissected *Staphylococcus* and *Streptococcus* pathogenesis through the systematic and integrated analysis of genomic, functional, metabolic, and expression data. Both genera were found to have a closed pangenome and lower expression variation for essential and highly persistent genes than for non-essential and low persistent genes.

The study of functional groups of proteins in the pangenome of *Staphylococcus* and *Streptococcus* involved in pathogenesis, indicates that domain shuffling and horizontal gene transfer have played an important role in the development and acquisition of pathogenesis proteins of *Staphylococcus* and *Streptococcus* species.

The analysis of bacterial clusters based on functional groups of proteins involved in pathogenesis shows that clustering of strains correlates with phenotypes such as zoonotic potential.

Comparison between *S. aureus* and *S. pyogenes* indicate three proteins, Enterotoxin B C-terminal domain, Enterotoxin B N-terminal beta-grasp domain together with several functionally equivalent proteins allow *Staphylococcus aureus* and *Streptococcus pyogenes* to cause necrotizing fasciitis.

We have also shown that prediction of the phenotype zoonotic potential only requires information about a few proteins, suggesting a direct causal relationship with zoonotic potential.

These findings will enable further research in each of the areas addressed, whereas the approaches and methods herein deployed provide a solid basis towards large-scale prediction of phenotypes based on genomic information.

## Methods

### Genome retrieval and annotation

All available completely assembled genomes of 235 Staphylococcus and 315 Streptococcus were downloaded as EMBL files from EBI-ENA using the Python EnaBrowserTool [73]. Lists of these genomes accession number, name and taxon ID can be found in supplementary material [Additional file 1 and 2]. Genome EMBL files were converted to RDF and de novo annotation was performed storing the results in a graph file per genome using SAPP, a Semantic Annotation Platform with Provenance [74] and the GBOL ontology [75]. Gene calling was performed using Prodigal with codon Table 5 [76]. Annotation was performed using InterProScan version 5.25 [77]. Protein domains were identified by InterProScan by their Pfam identifier [78]. The GNU “parallel” package was used to perform all of the above steps in parallel [74].

The graph files were loaded in GraphDB Free version 8.4.1 in order to query the annotated genomes. Additionally, taxonomic information from UniProt was downloaded in RDF format and loaded in GraphDB. The GraphDB SPARQL endpoint was queried using the Python SPARQLWrapper [79] package and the R Curl package [80] to retrieve information and store them as matrixes given in supplementary material [see Additional file 3 and 4]. These files were used for all subsequent analyses.

### Estimation of the size of the pan- and core genome

Proteins were compared based on their Pfam domain content. We defined protein domain content as the alphabetical order of all unique domains associated with a given protein. A matrix was built to collect information on the presence or absence of proteins in each genome. Two sampling approaches were used: 1) genomes were randomly selected from all genomes in the analysed genera and 2) a maximum of one genome per species was selected to avoid bias introduced by species with many sequenced genomes. One up to the total number of genomes were sampled and analysed using the micropan R package [24] to investigate the effect of the number of genomes on the estimation of the size of the pan- and core genome. Additionally, these samples we used to estimate the sizes of the pan- and core genome using a binomial mixture model using the micropan BinomixEstimate function with 5000 permutations and a core detect probability of 1. The process was repeated 10 times to estimate the variance of the estimated size of the pan- and core genomes. The Heaps’ function was used to fit a Heaps’ regression model; α > 1 indicates convergence of the size of the pan-genome and that it is closed.

### Variability of gene expression and its association to persistence

Gene variability was calculated based on 156 *S. aureus* RNA samples from 44 conditions ranging from laboratory to conditions mimicking infection, measured by Tiling arrays [42]. These 44 conditions can be categorized in four groups:

1) rich medium (TSB), 2) minimal medium (CDM), 3) cell culture media (RPMI, pMEM) 4) in human plasma (plasma), 5) growth with human bronchial epithelial cell line S9 and the human monocyte cell line THP-1.

Samples were taken at different time points and for infection simulations oxygen availability was limited at later time points. For a complete description of the conditions we refer to S1 Data in the original paper by U. Mader et al. [42]. For every gene we considered its expression profile over all samples and a variability value was calculated as the ratio between the standard deviation and the mean expression value using the same approach as in Koehorst et al. [9].

### Protein persistence and essentiality

We defined the persistence of a gene as

$$ Persistence=\frac{N\ (orth)}{N} $$

where *N (orth)* is the number of genomes carrying a given orthologue and *N* is the number of genomes searched [81]. Orthologue genes were identified as genes with identical protein domain content. Locus tags associated to the genes were inferred from the original annotation and used to integrated genome wide gene essentiality data from transposon mutagenesis studies for *Staphylococcus* strains S0385 grown on whole porcine blood [82], NCTC8325 Newman grown on BHI broth [43] and JE2 grown on Handke mannitol medium [83] and *Streptococcus* strains *S. pyogenes* M1T1 strain 5448 and M49 strain NZ131 grown in rich Todd-Hewitt Yeast (THY) medium [84].

### GEM-based predictions of essentiality

Gene essentiality analysis based on genome scale modelling was performed using the genome-scale, constraint-based metabolic model (GEM) of *S. aureus* NTCTC 8325 [8] and the GEM model of *S. pyogenes* M49 [85]. First, a minimal medium was determined using the ‘cobrapy minimal_medium function’. All carbon, nitrogen, sulphur and phosphor sources from the medium that could support growth were detected by substituting the default carbon, nitrogen, sulphur and phosphor sources. All combinations of minimal media containing these carbon, nitrogen, sulphur and phosphor sources were generated.

Gene essentiality for all combinations of minimal media containing these carbon, nitrogen, sulphur and phosphor were tested by performing single gene deletions followed by flux balance analysis optimizing for growth. If a gene knock-out reduced predicted growth for the media compositions below 1% the gene was considered conditionally essential. Genes predicted to be conditionally essential in at least 90% of the in-silico media compositions were marked as essential.

All optimizations were performed using the Gurobi optimizer 8.1 [86] with COBRApy 0.13.4 [87] and Python 3.6.

### Functional analysis

Genome information was retrieved from associated literature and from the Biosample database [88], including serotype information and zoonotic potential and isolation-host. Zoonotic classification was derived from literature for *S. inae* [89–92]*, S. agalactiae* [69]*, S. dysgalactiae, S. equi* [93–95] and at the serotype level for *S. suis* [96–98]. For all zoonotic *Streptococcus* species data about the isolation host was retrieved from the Biosamples databases [99] or literature [63–65, 100–107]. Additional Gene Ontology (GO) annotation from the GODM (GO Domain Miner) database [33] was added to proteins based on their domain content, increasing the number of GO terms by approximately 10-fold compared to GO term annotation retrieved from the InterPro database. Literature was used to select 17 GO terms in the Biological process ontology with known or suspected association to pathogenesis [108–112] (Table 5).

The presence/absence matrix of proteins was filtered on proteins annotated with any of the 17 GO terms (Table 5) or their descendent GO terms using the R GO.db package [113]. The filtered matrix was used to calculate the Euclidean distance between genomes. Hierarchical complete-linkage clustering was used to generate dendrograms. These GO-specific dendrograms were compared to a reference dendrogram based on all proteins.

**Table 5:** Gene Ontology (GO) terms used to select proteins based on their domain content for functional trees, PCA and t-SNA analysis. GO terms that are direct children of the ‘Biological process’ GO term are marked with an asterisk (*)

GO ID	Description
GO:0008150	Biological process
GO:0008152	*Metabolic process
GO:0017144	Drug metabolic process
GO:0042493	Response to drug
GO:0023052	*Signalling
GO:0065007	*Biological regulation
GO:0022610	*Biological adhesion
GO:0044406	Adhesion of symbiont to host
GO:0051704	Multi-organism process
GO:0044419	Inter species interaction between organisms
GO:0042710	Biofilm formation
GO:0098743	Cell aggregation
GO:0044403	Symbiont process
GO:0009372	Quorum sensing
GO:0035821	Modification of morphology or physiology of other organism
GO:0009405	Pathogenesis

Because these dendrograms are based on annotation of proteins for a specific function, we will refer to them as ‘functional trees’. Euclidean distances of genomes in the functional trees and the reference tree were calculated and scaled to values between 0 and 1 using the R scale function using the minimum value for centring, and (min – max) for scaling. Scaled values were used to calculate similarity scores for the position of genomes in each functional tree compared to the reference. These similarity scores were calculated as the Pearson correlation between the scaled Euclidean distances of genomes in the functional tree and the scaled Euclidean distance in the reference tree. Interactive heatmap were generated showing the presence and absence of proteins per genome, while showing the similarity in the side column to highlight differences compared to the reference tree. These interactive graphs were generated using the dendextend and heatmaply packages [114, 115]. Similarity scores for functional trees were calculated using the dendextend cor_cophenetic function.

Matrix manipulations, Principal Component Analysis (PCA), t-Distributed Stochastic Neighbour Embedding (t-SNE) and graphs were performed using R 3.6.1 [116], the prcomp command, and the Rtsne 0.15 [117] and ggplot2 2.3.2.1 [118] packages. t-SNE was performed with default parameters.

### Random Forest classification

Proteins belonging to GO categories ‘pathogenesis’, ‘modification of morphology or physiology of other organisms’ and ‘biological adhesions’, were used to train Random Forests classifiers for *S. suis* and *S. agalactiae* strains to predict whether they belong to the class ‘zoonotic potential’ on ‘non-zoonotic potential’. This classification was based on the clustering of in PCA and t-SNE plots which revealed the presence of a zoonotic and a non-zoonotic group of strains. Data was split in 75% training data and 25% validation data.

Data was loaded using Python 3.6, pandas 0.24.2. Skicit-learn 0.20.3 used to load data and train Random Forest classifiers. Treeinterpreter 0.1.0 was used to interpret feature (protein) importance for classification in general and feature contribution to predict specific classes. Grid search for 300 combinations of parameters was performed optimizing the parameters n_estimators, max_features, max_depth, min_samples_split, and the min_samples. Iterative rounds of feature reduction, that is removal of the protein which least contribute to the classification, followed by hyper parameter optimization, was used to find the minimal set of features (proteins) needed to classify both training and test data. Feature importance and contribution were plotted using matplotlib 3.0.3.

## Supplementary Information

**Additional file 1.** List of Staphylococcus genomes

**Additional file 2.** List of Streptococcus genomes

**Additional file 3.** Staphylococcus All proteins

**Additional file 4.** Streptococcus All proteins

**Additional file 5.** Estimated Pan and Core genome size, Heaps Analysis

**Additional file 6.** Staphylococcus Functional trees

**Additional file 7.** Staphylococcus PCA

**Additional file 8.** Staphylococcus t-SNE

**Additional file 9.** Streptococcus Functional trees

**Additional file 10.** Streptococcus PCA

**Additional file 11.** Streptococcus t-SNE

**Additional file 12.** Staphylococcus & Streptococcus combined PCA

**Additional file 13.** Staphylococcus & Streptococcus combined t-SNE

**Additional file 14.** Optimal hyper parameters

## Data Availability

The authors declare that all data supporting the findings of this study are available within the article and its supplementary information.

## Abbreviations

Exp: Experimentally
GEM: Genome scale metabolic model
GO: Genome Ontology
IgA: Immunoglobulin A
IgG: Immunoglobulin G
t-SNE: T-distributed Stochastic Neighbour Embedding
