# Correction to: Phenotype and multi-omics comparison of *Staphylococcus* and *Streptococcus* uncovers pathogenic traits and predicts zoonotic potential

**DOI:** 10.1186/s12864-021-07707-x

**Published:** 2021-05-25

**Authors:** Niels A. Zondervan, Vitor A. P. Martins dos Santos, Maria Suarez-Diez, Edoardo Saccenti

**Affiliations:** 1 Laboratory of Systems and Synthetic Biology, Wageningen University & Research, Stippeneng 4, 6708 WE Wageningen, Netherlands; 2 LifeGlimmer GmBH, Markelstraße 38, 12163 Berlin, Germany

**Correction to: BMC Genomics 22, 102 (2021)**

**https://doi.org/10.1186/s12864-021-07388-6**

Following publication of the original article [1], it was reported that there was a missing reference in the Methods section and should have been reference [2] at the end of the following sentence:

The GNU “parallel” package was used to perform all of the above steps in parallel [2].

This reference has been inserted in the original article, the subsequent references have been renumbered, and the original article has been corrected.
