# Supplementary material for: Phenotype and multi-omics comparison of Staphylococcus and Streptococcus uncovers pathogenic traits and predicts zoonotic potential
Source: BMC Genomics. 2021 Feb 4;22:102. doi: 10.1186/s12864-021-07388-6 (PMC7860044; doi:10.1186/s12864-021-07388-6)
Supplement: Supplementary file 5 — Additional file 5. Estimated Pan and Core genome size, Heaps Analysis [file 12864_2021_7388_MOESM5_ESM.pdf]

Heaps regression model – closedness of the pan genome

Heaps regressions model to estimate the closedness of the pan genome. The plots show the alpha value, an α >1 is an indication the pan genome being closed. A closed pan genome means sequencing additional genomes is expected to only add little additional information (new proteins).

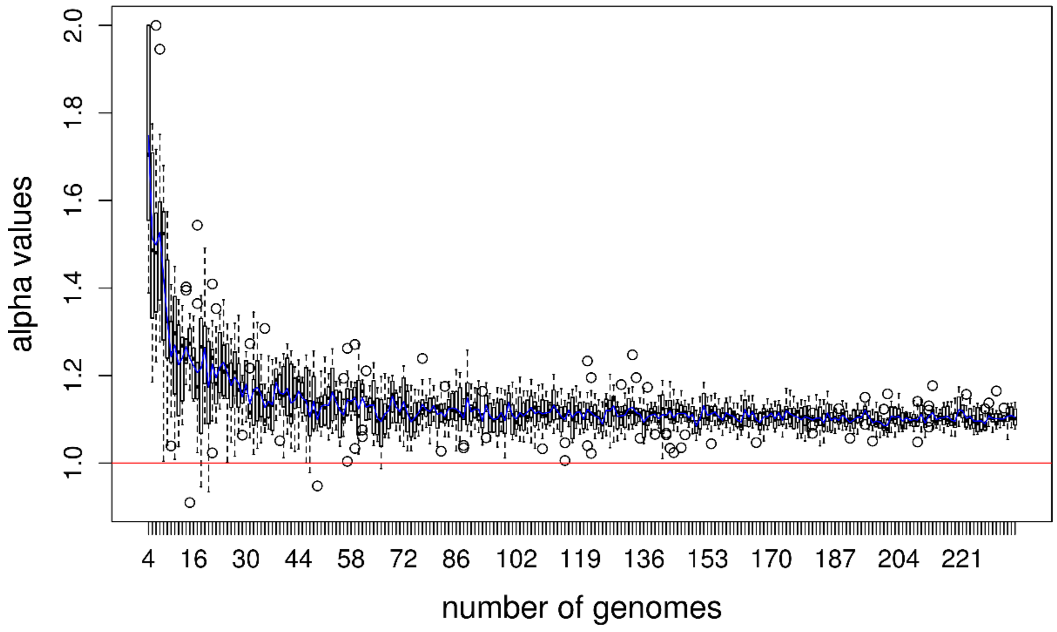

Figure 1 Heaps regression model of 1 up to the total number of Staphylococcus

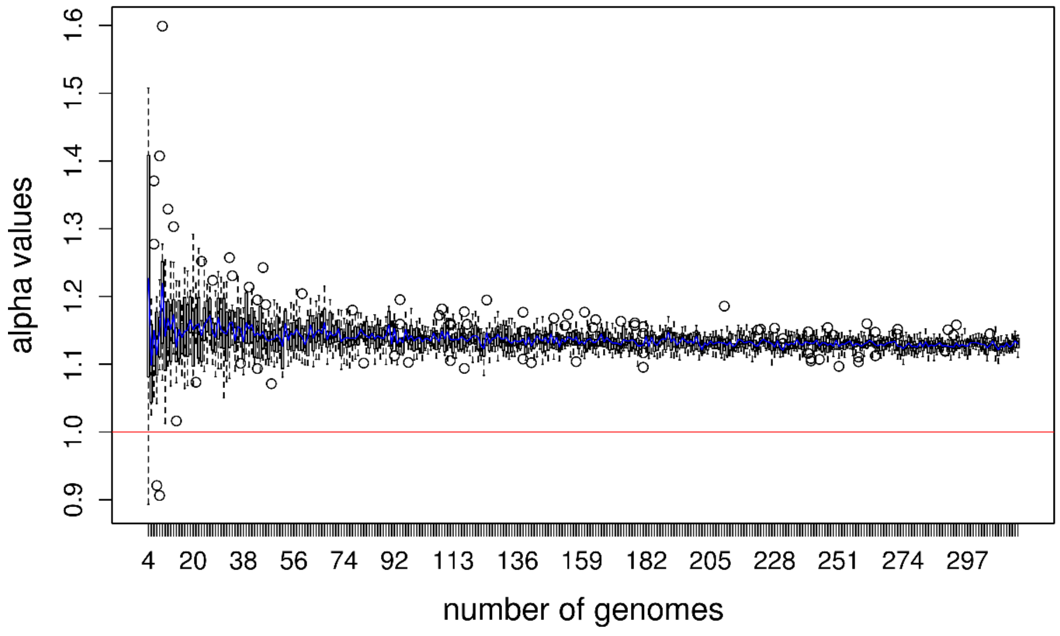

Figure 2 Heaps regression model of 1 up to the total number of Streptococcus *genomes*

The initial peak in the Figure 1 indicates there might be a subpopulation of highly similar genomes (*S. aureus* genomes) among the *Staphylococcus* genomes. This hypothesis was tested by repeating the same analysis only allowing one genome per species to be drawn. As can be seen in Figure 3 and Figure 4, the initial high alpha value in the Heaps regression model for *Staphylococcus* disappears when only allowing one genome per species, confirming the hypothesis.

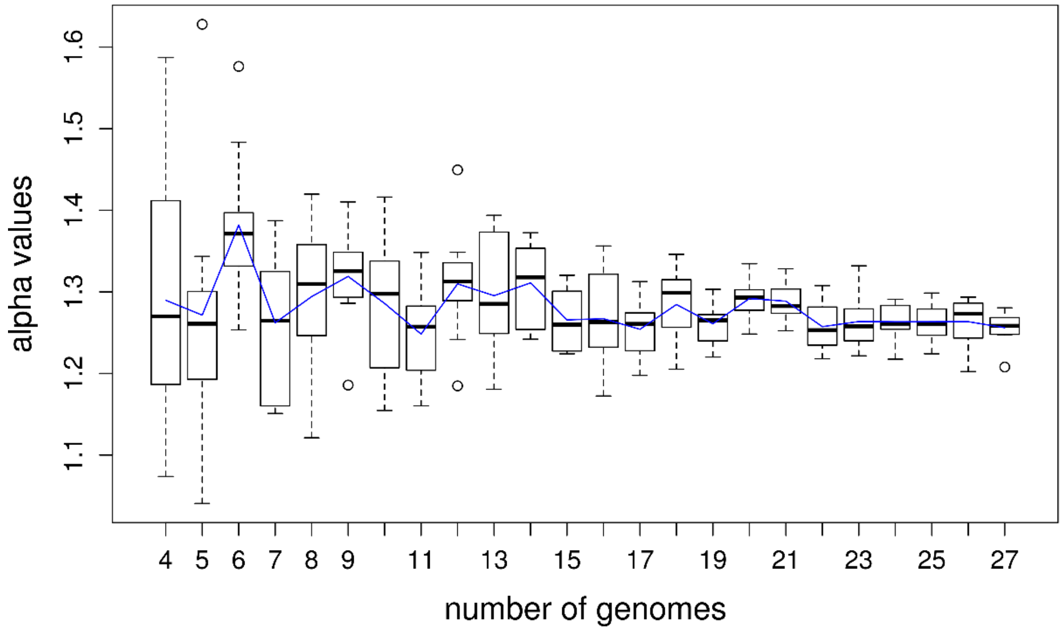

Figure 3 Heaps regression model of 1 up to the total number of Streptococcus genomes allowing a maximum of 1 sampled genome per species

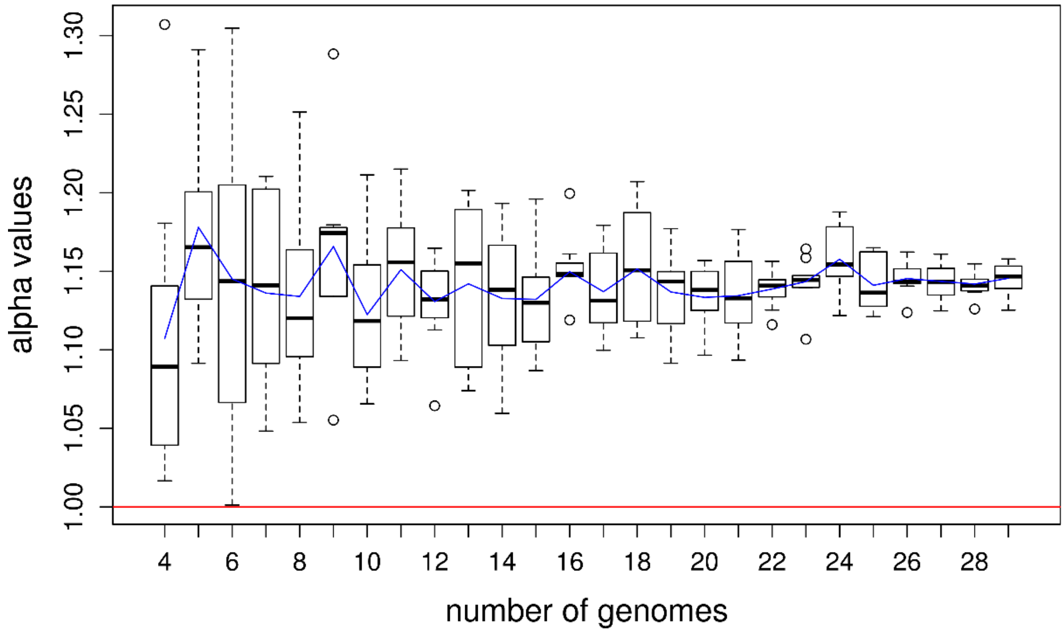

Figure 4 Heaps regression model of 1 up to the total number of Streptococcus genomes allowing a maximum of 1 sampled genome per species
