# Supplementary material for: Phenotype and multi-omics comparison of Staphylococcus and Streptococcus uncovers pathogenic traits and predicts zoonotic potential
Source: BMC Genomics. 2021 Feb 4;22:102. doi: 10.1186/s12864-021-07388-6 (PMC7860044; doi:10.1186/s12864-021-07388-6)

Staphylococcus All architectures

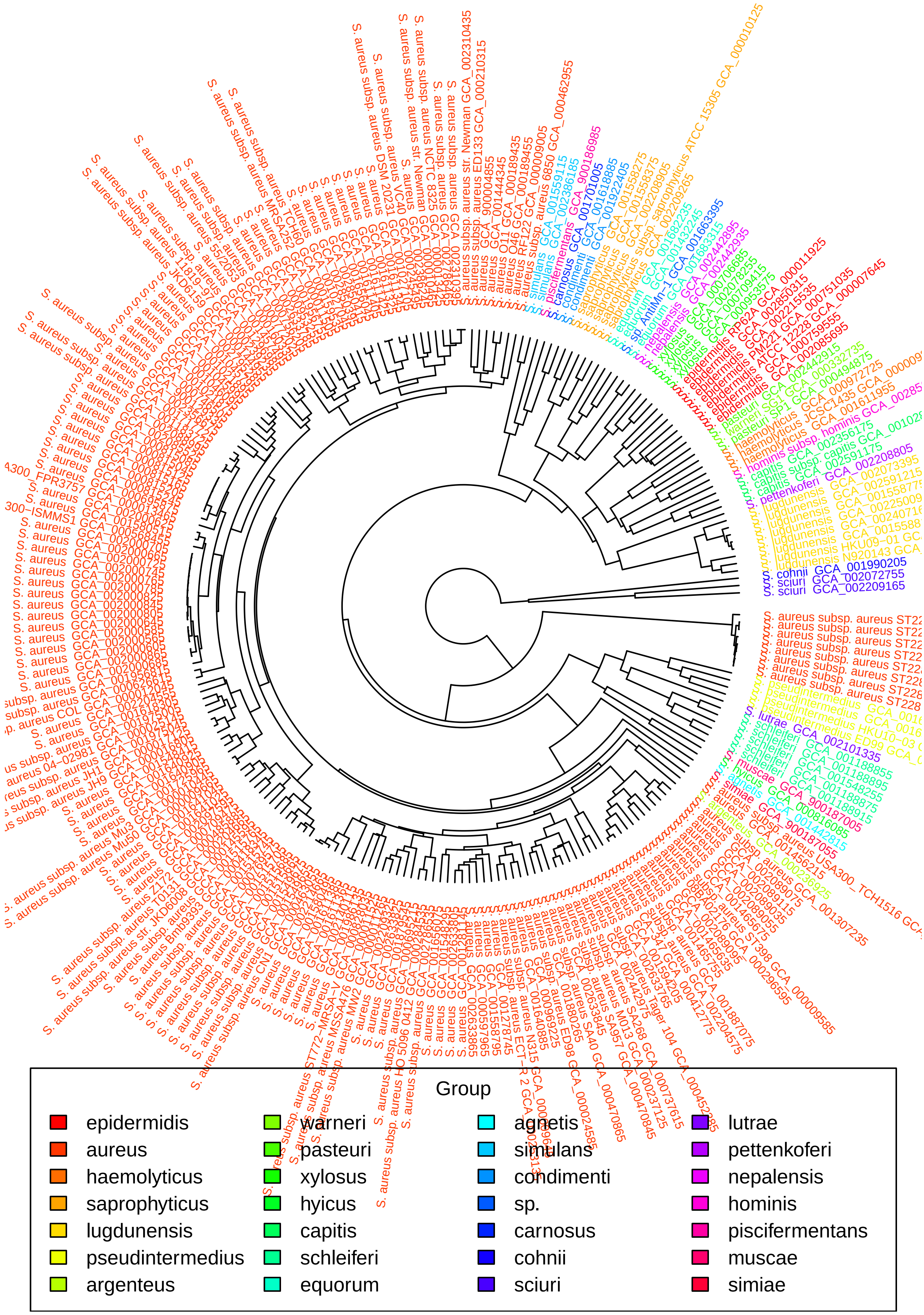

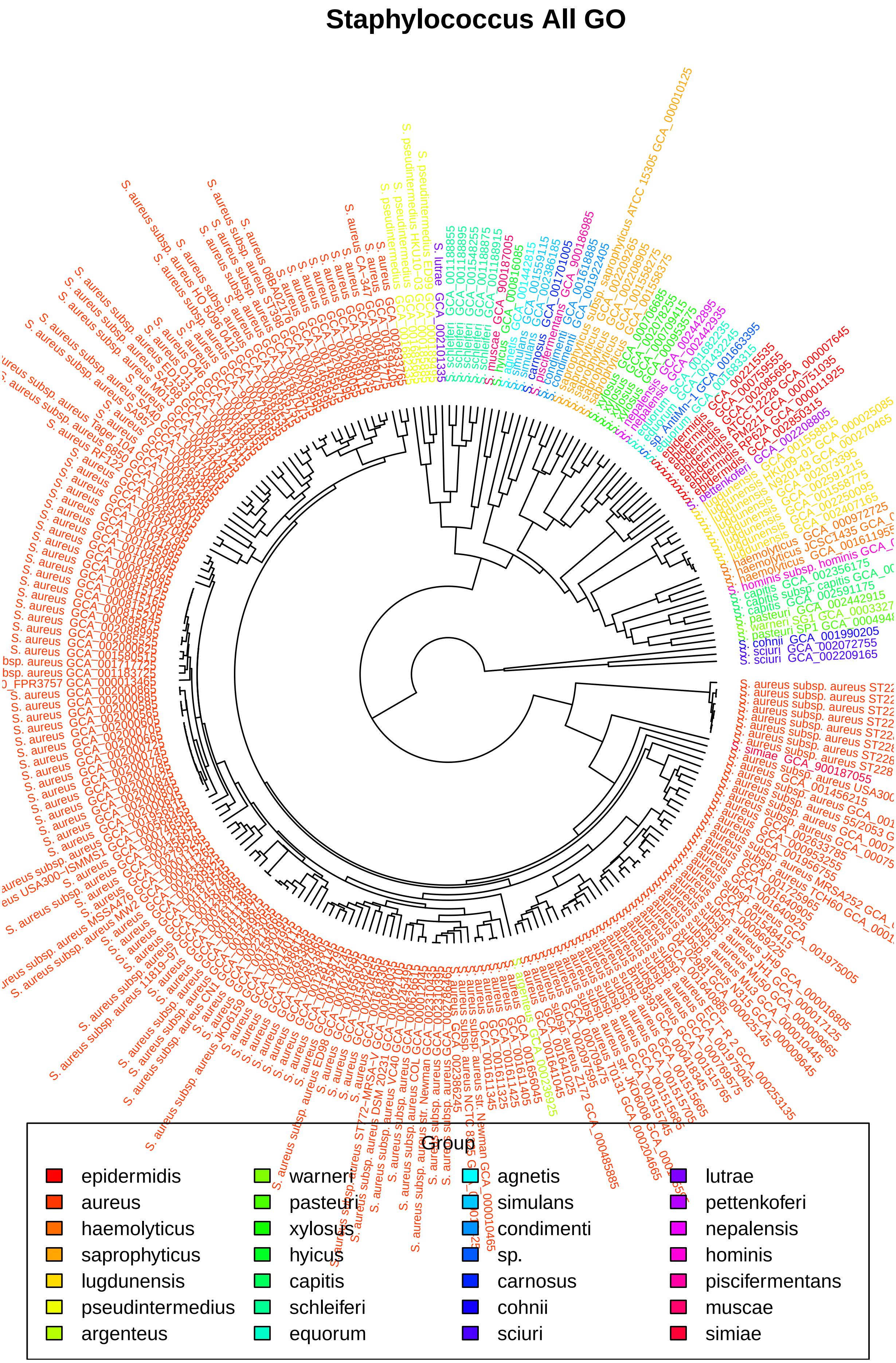
Staphylococcus All GO
Group
epidermidis
aureus
haemolyticus
saprophyticus
lugdunensis
pseudintermedius
argenteus
warneri
pasteuri
xylosus
hyicus
capitis
schleiferi
equorum
agnetis
simulans
condimenti
sp.
carnosus
cohnii
sciuri
lutrae
pettenkoferi
nepalensis
hominis
piscifermentans
muscae
simiae

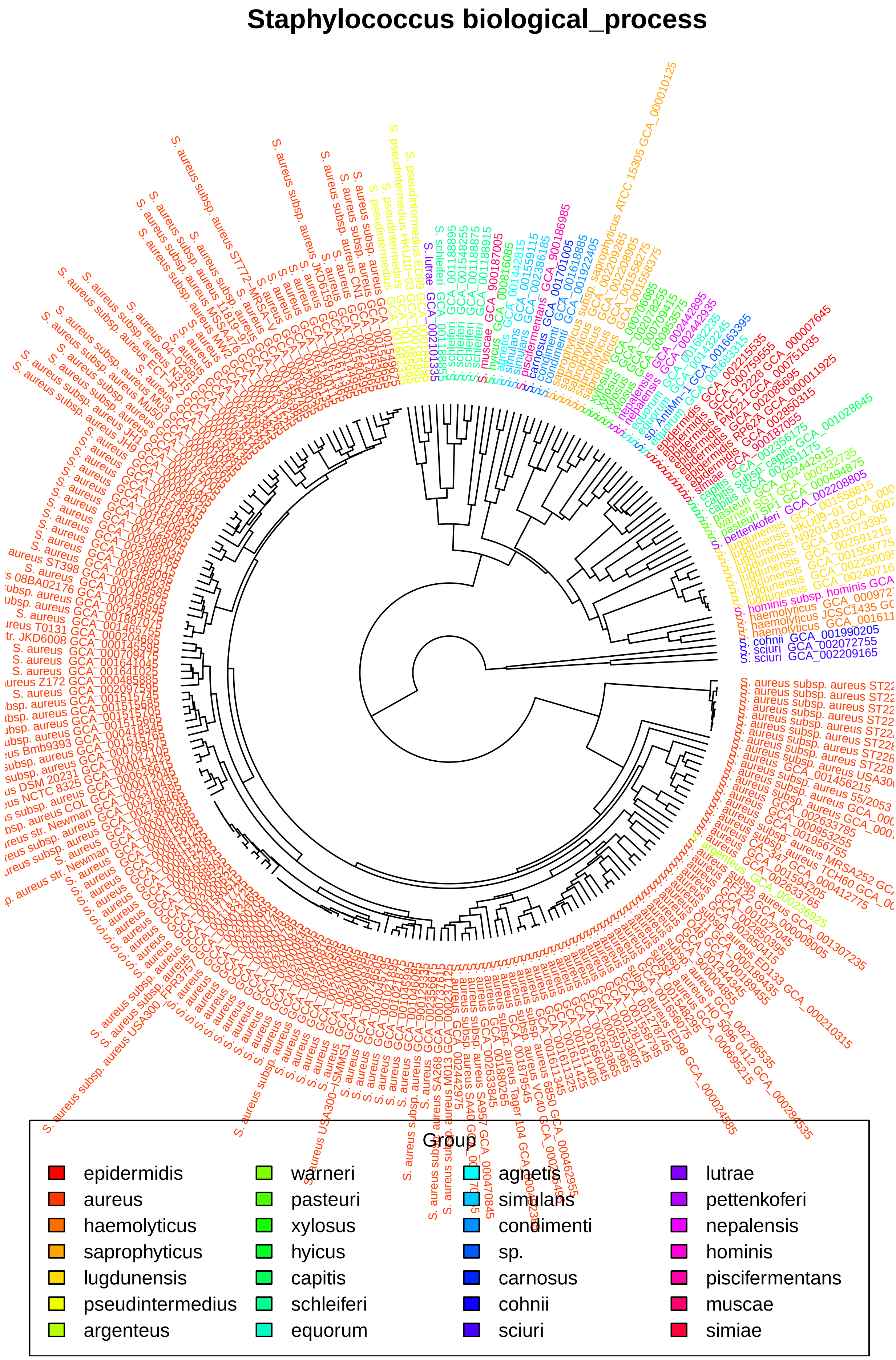
Staphylococcus biological_process
Group
epidermidis
aureus
haemolyticus
saprophyticus
lugdunensis
pseudintermedius
argenteus
warneri
pasteuri
xylosus
hyicus
capitis
schleiferi
equorum
agnetis
simulans
condimenti
sp.
carnosus
cohnii
sciuri
lutrae
pettenkoferi
nepalensis
hominis
piscifermentans
muscae
simiae

Staphylococcus metabolic_process

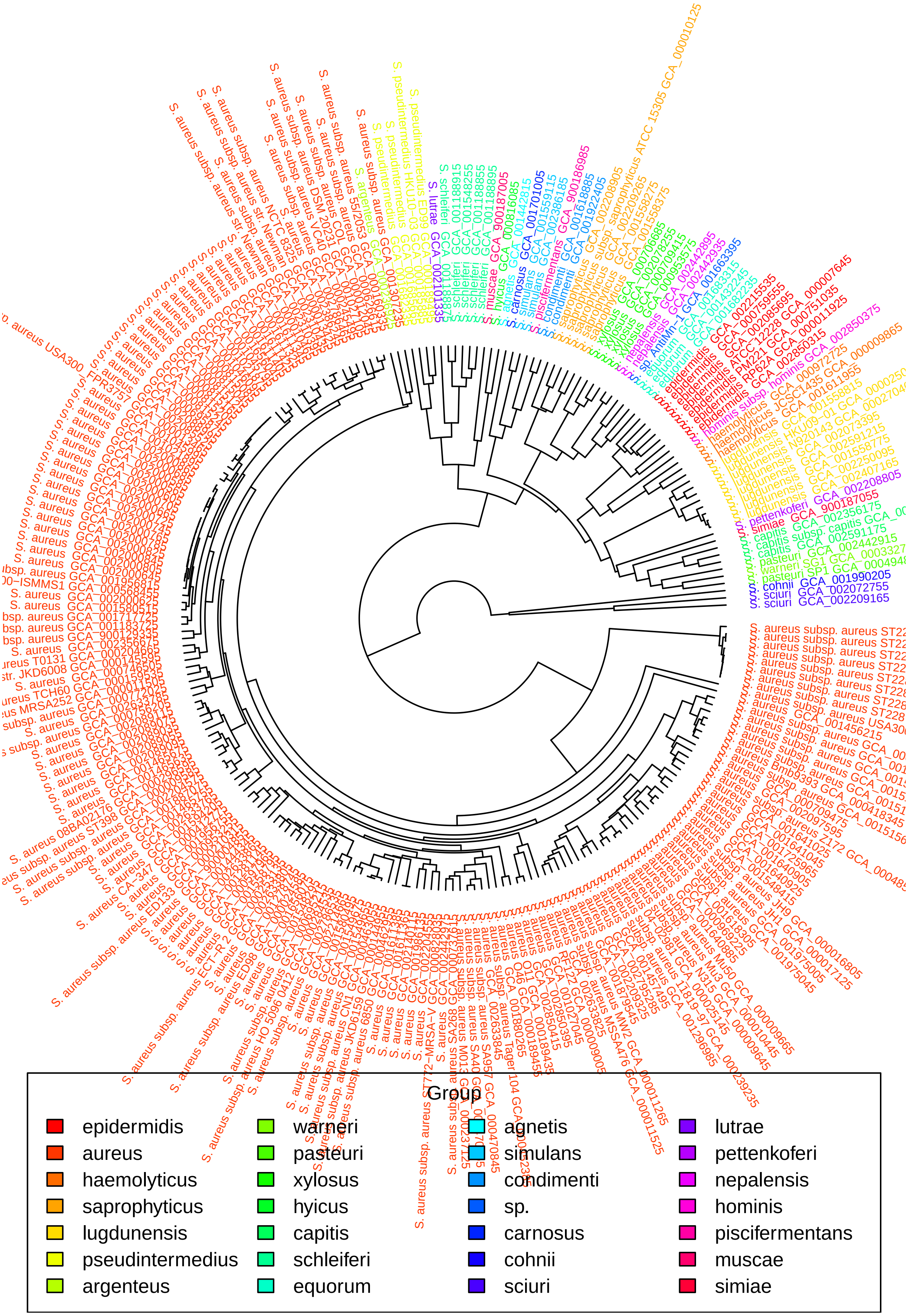

Staphylococcus −−drug_metabolic_process

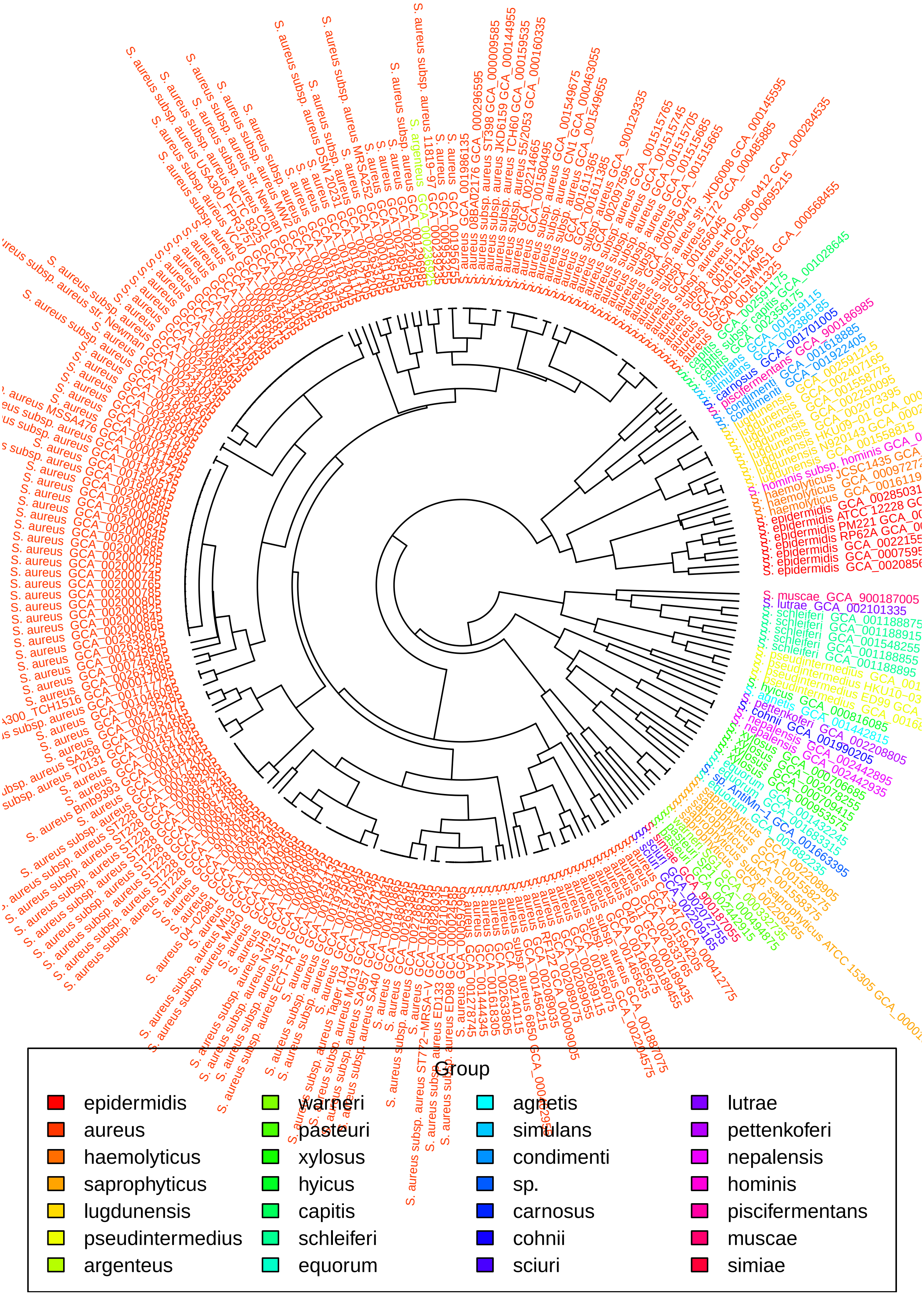

Staphylococcus response_to_drug

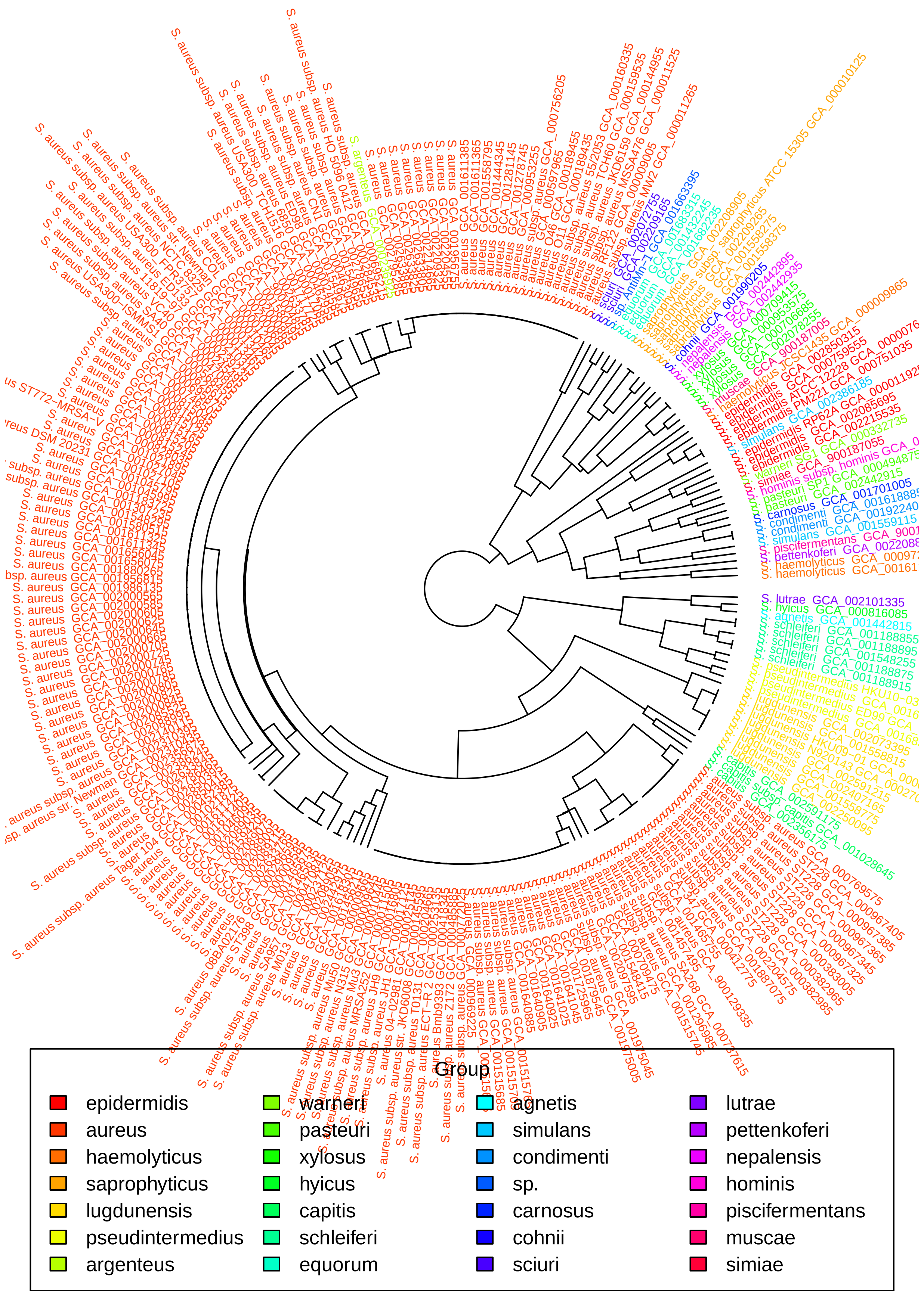

Staphylococcus signaling

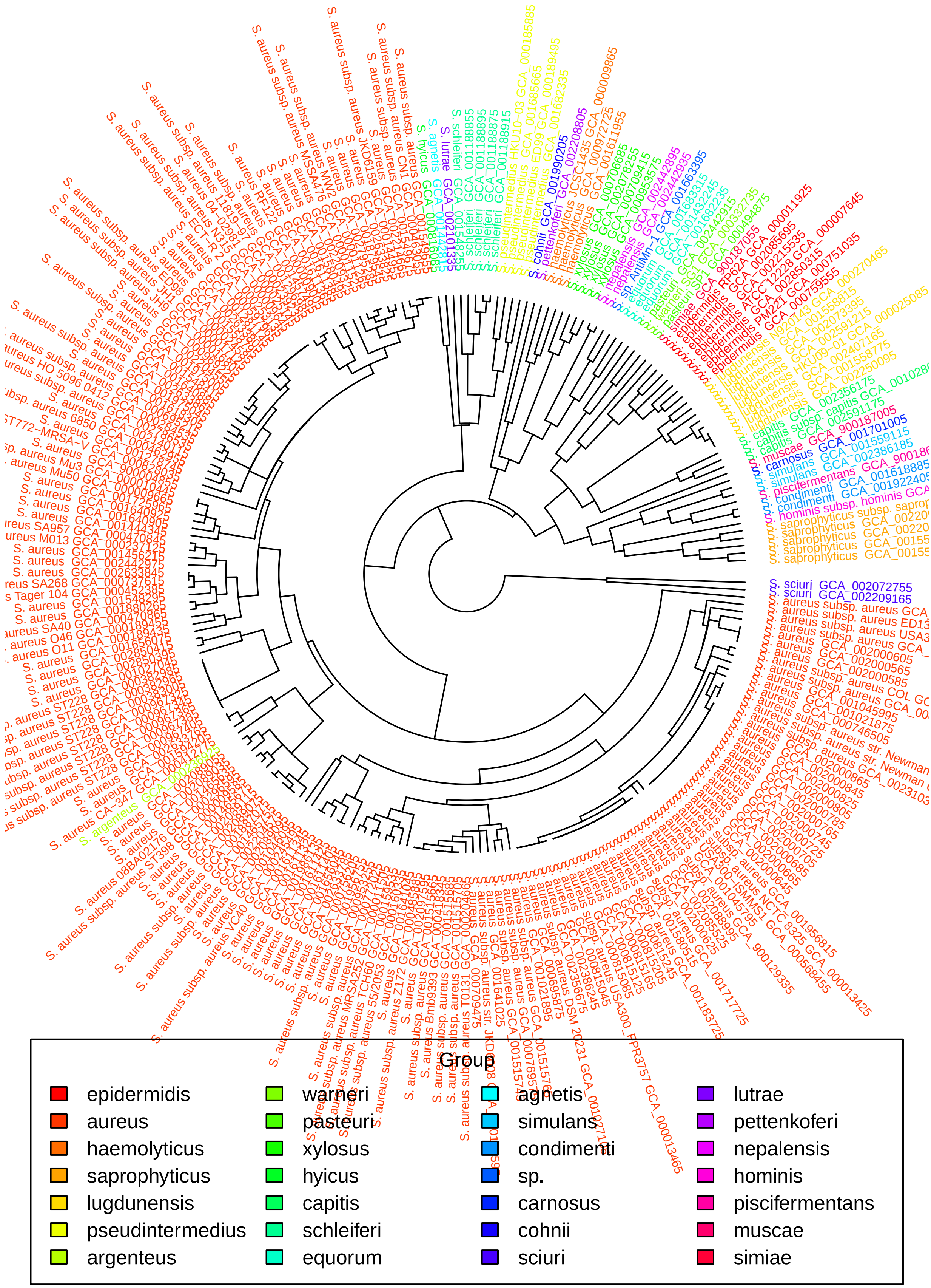

Staphylococcus biological_regulation

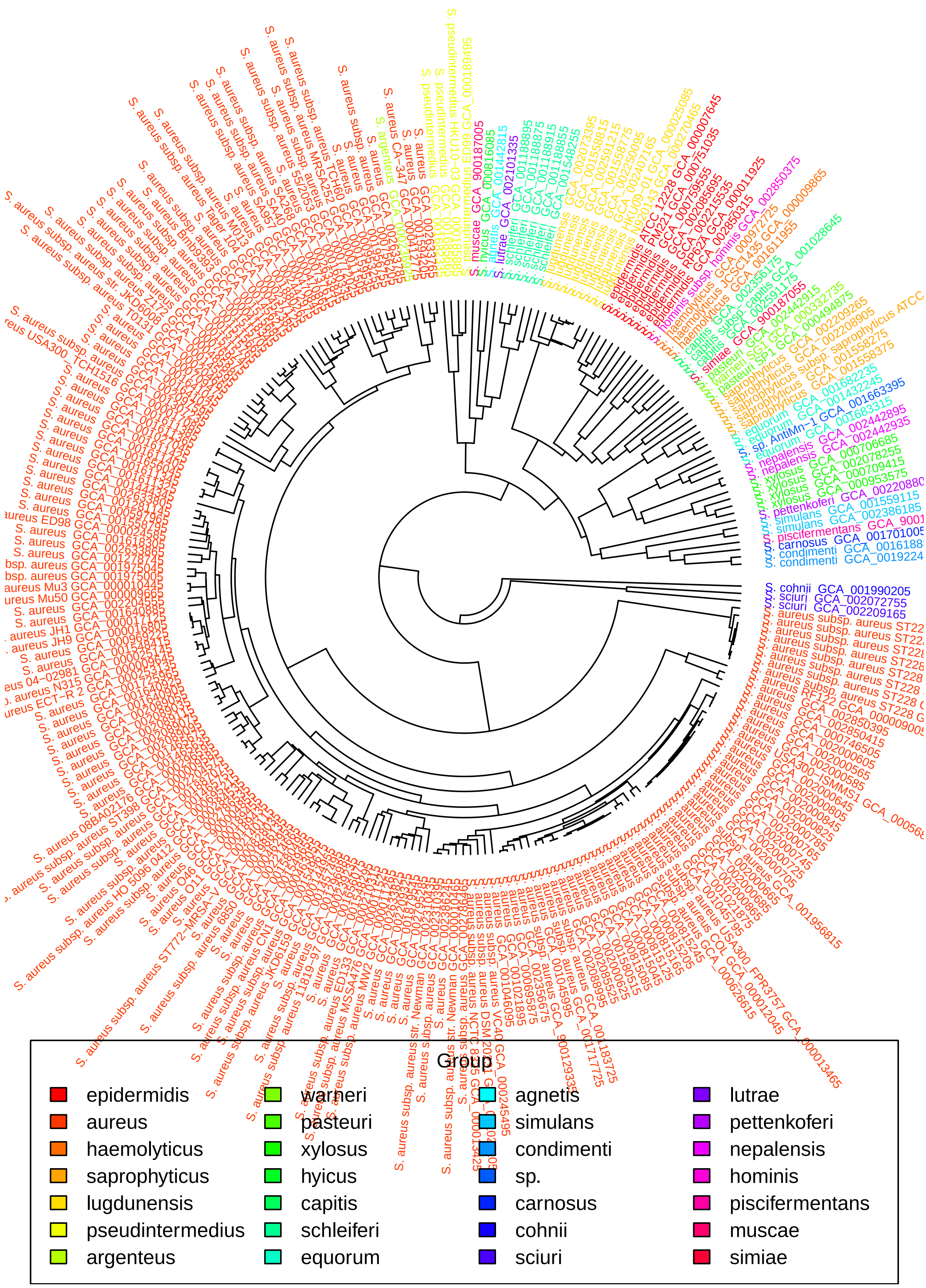

Staphylococcus −biological_adhesion

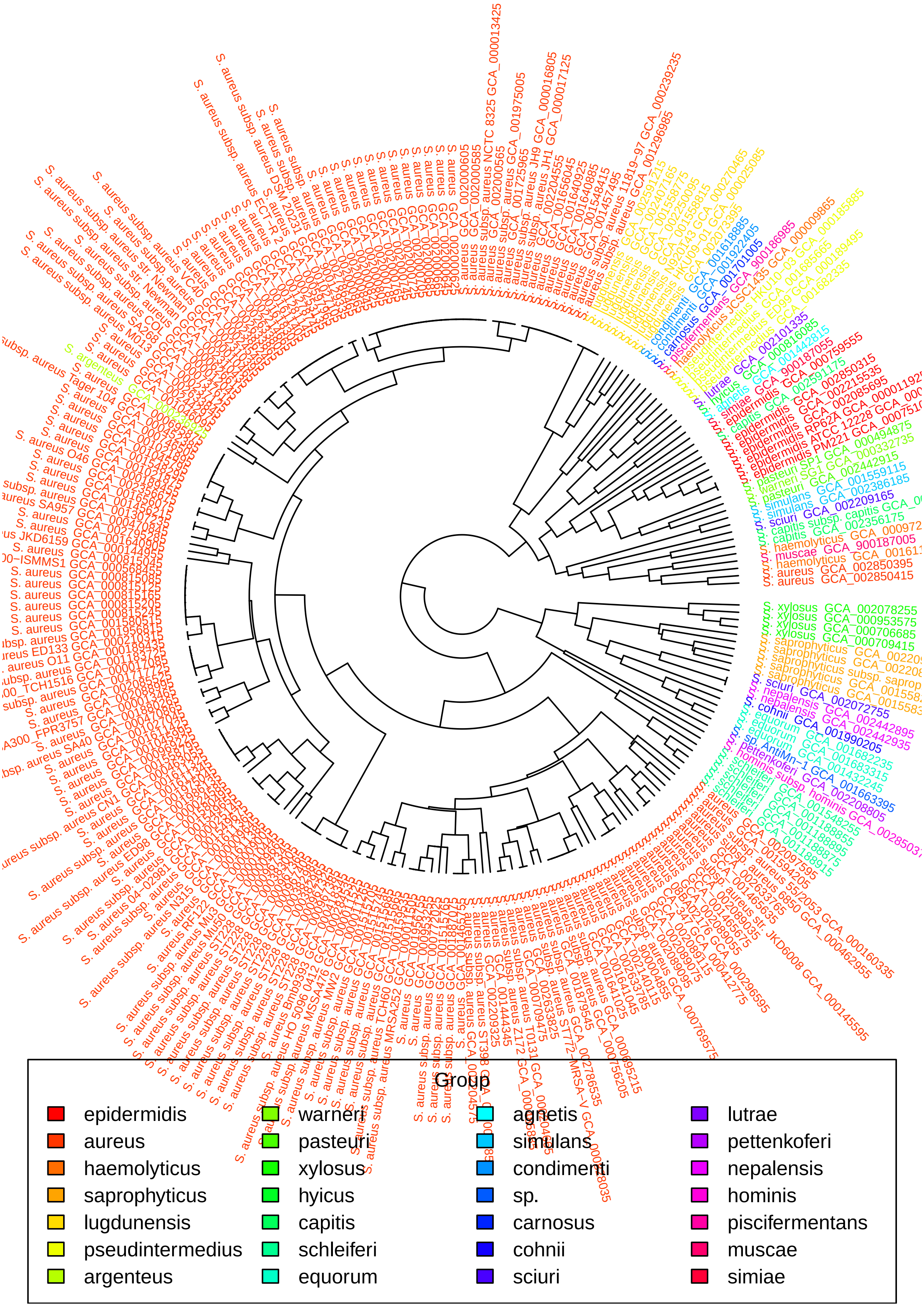

Staphylococcus --adhesion_of_symbiont_to_host

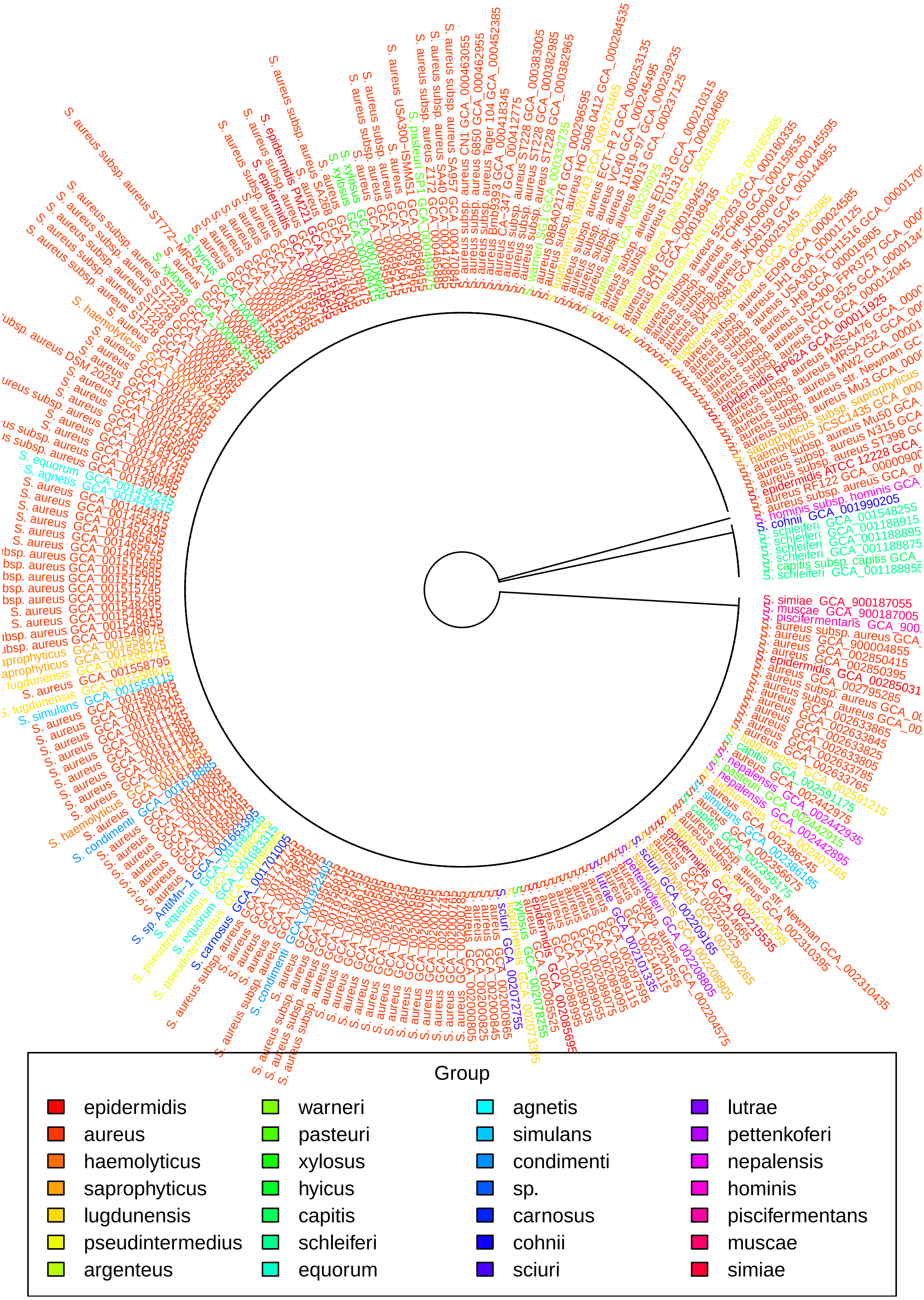

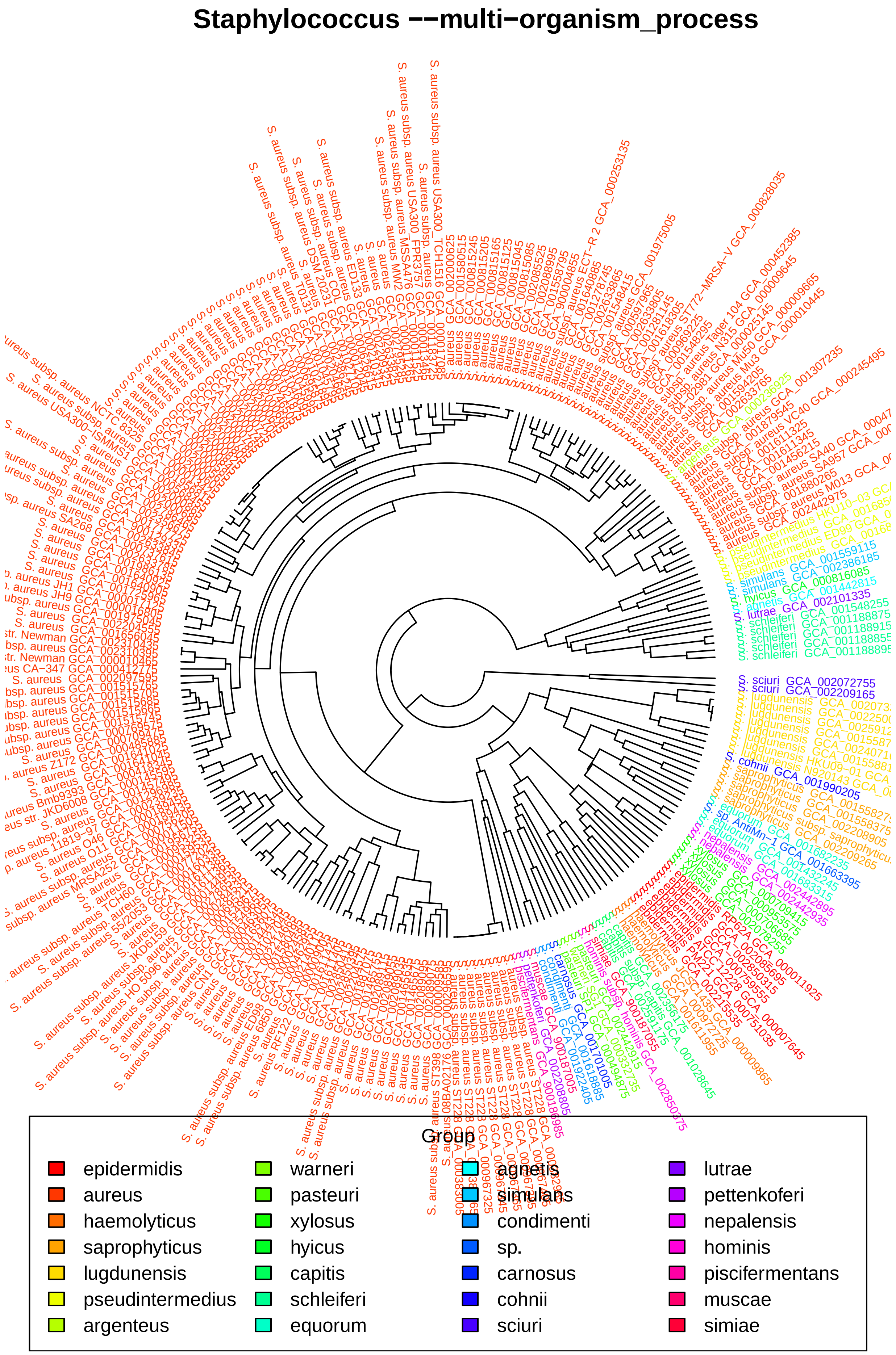
Staphylococcus --multi-organism_process
Group
epidermidis
aureus
haemolyticus
saprophyticus
lugdunensis
pseudintermedius
argenteus
warneri
pasteuri
xylosus
hyicus
capitis
schleiferi
equorum
agnetis
simulans
condimenti
sp.
carnosus
cohnii
sciuri
lutrae
pettenkoferi
nepalensis
hominis
piscifermentans
muscae
simiae

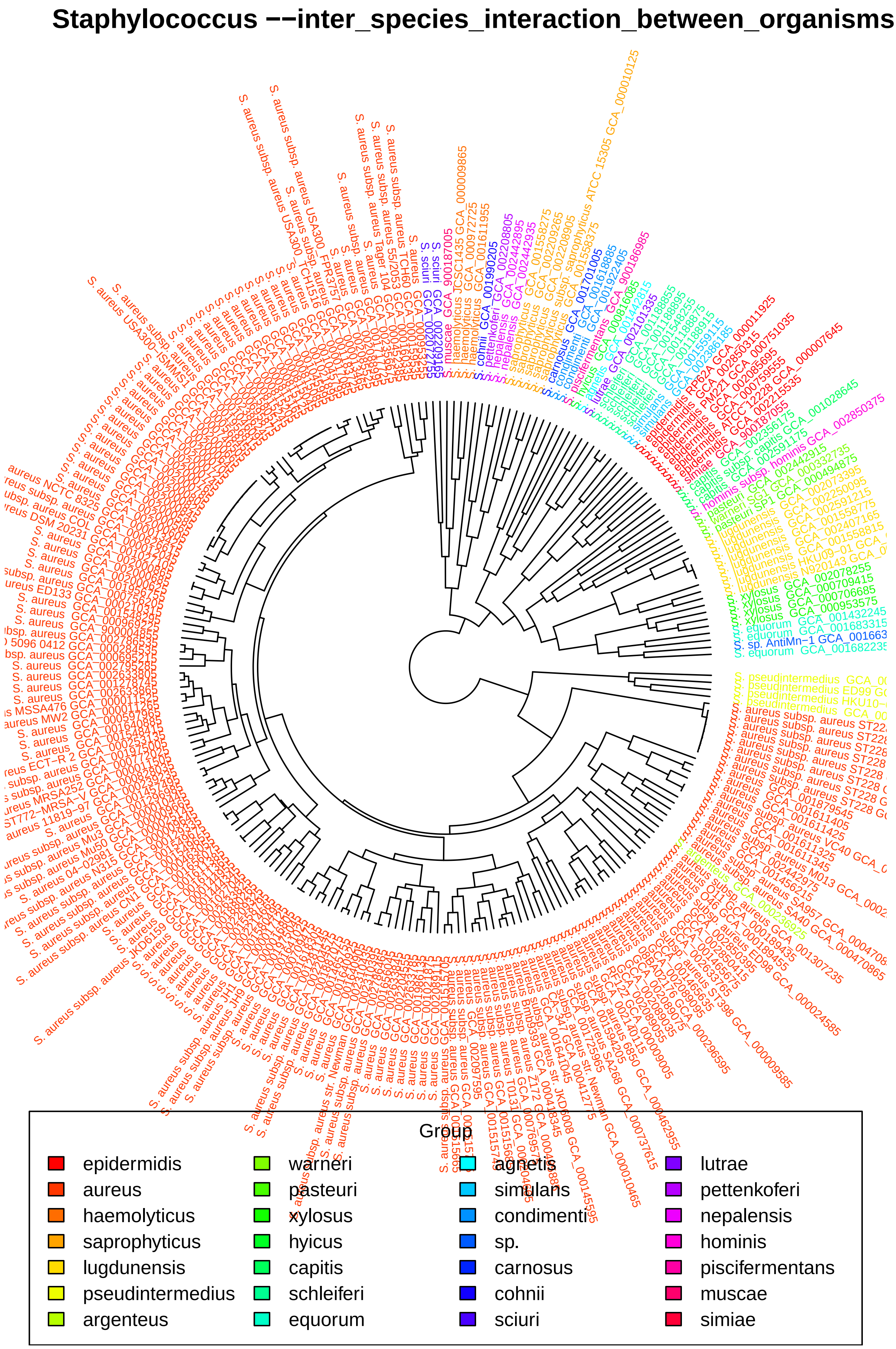

Staphylococcus --inter_species_interaction_between_organisms
Group
epidermidis
aureus
haemolyticus
saprophyticus
lugdunensis
pseudintermedius
argenteus
warneri
pasteuri
xylosus
hyicus
capitis
schleiferi
equorum
agnetis
simulans
condimenti
sp.
carnosus
cohnii
sciuri
lutrae
pettenkoferi
nepalensis
hominis
piscifermentans
muscae
simiae

Staphylococcus −−biofilm_formation

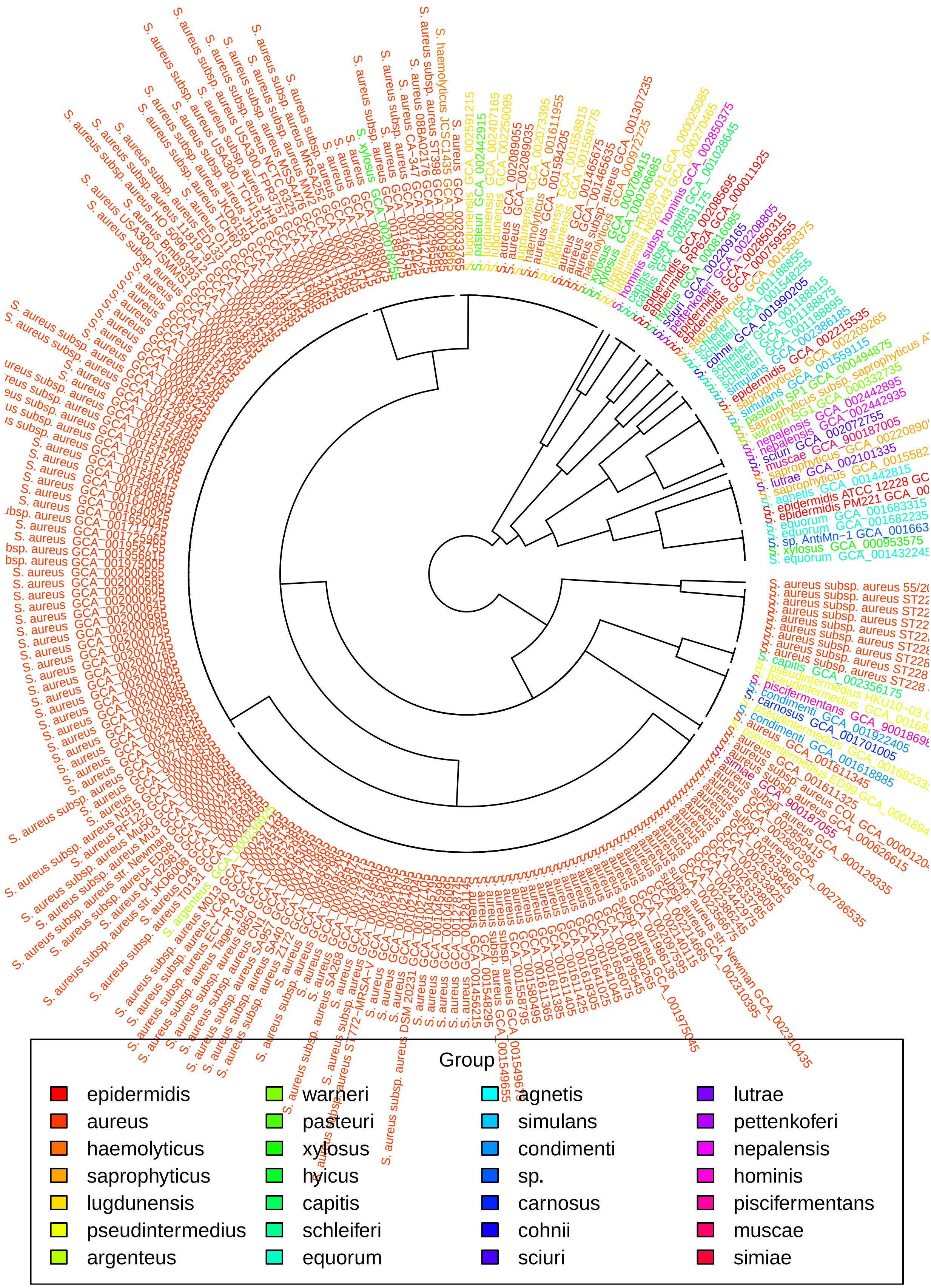

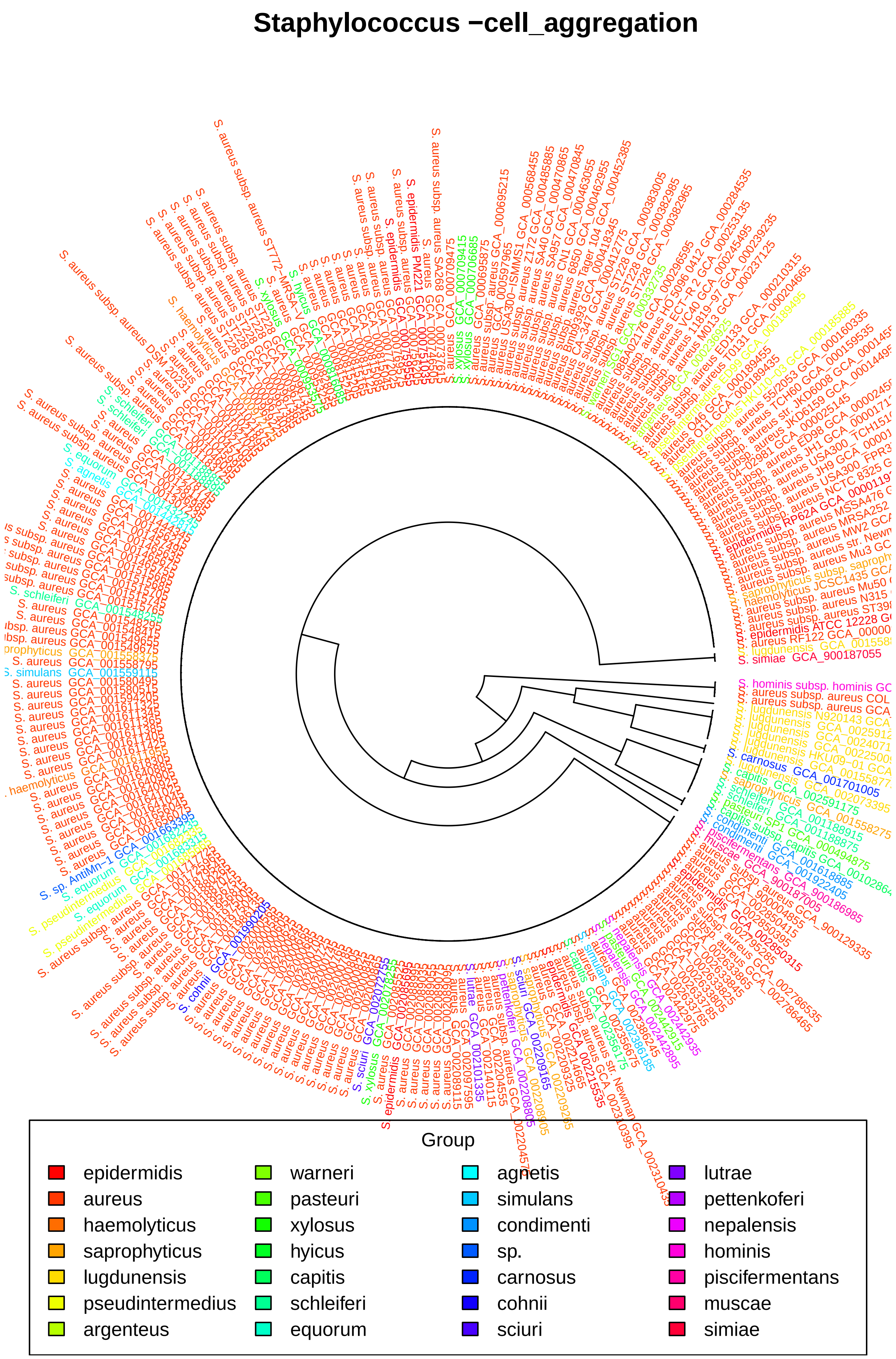
Staphylococcus −cell_aggregation
Group
epidermidis
aureus
haemolyticus
saprophyticus
lugdunensis
pseudintermedius
argenteus
warneri
pasteuri
xylosus
hyicus
capitis
schleiferi
equorum
agnetis
simulans
condimenti
sp.
carnosus
cohnii
sciuri
lutrae
pettenkoferi
nepalensis
hominis
piscifermentans
muscae
simiae

Staphylococcus −−symbiont_process

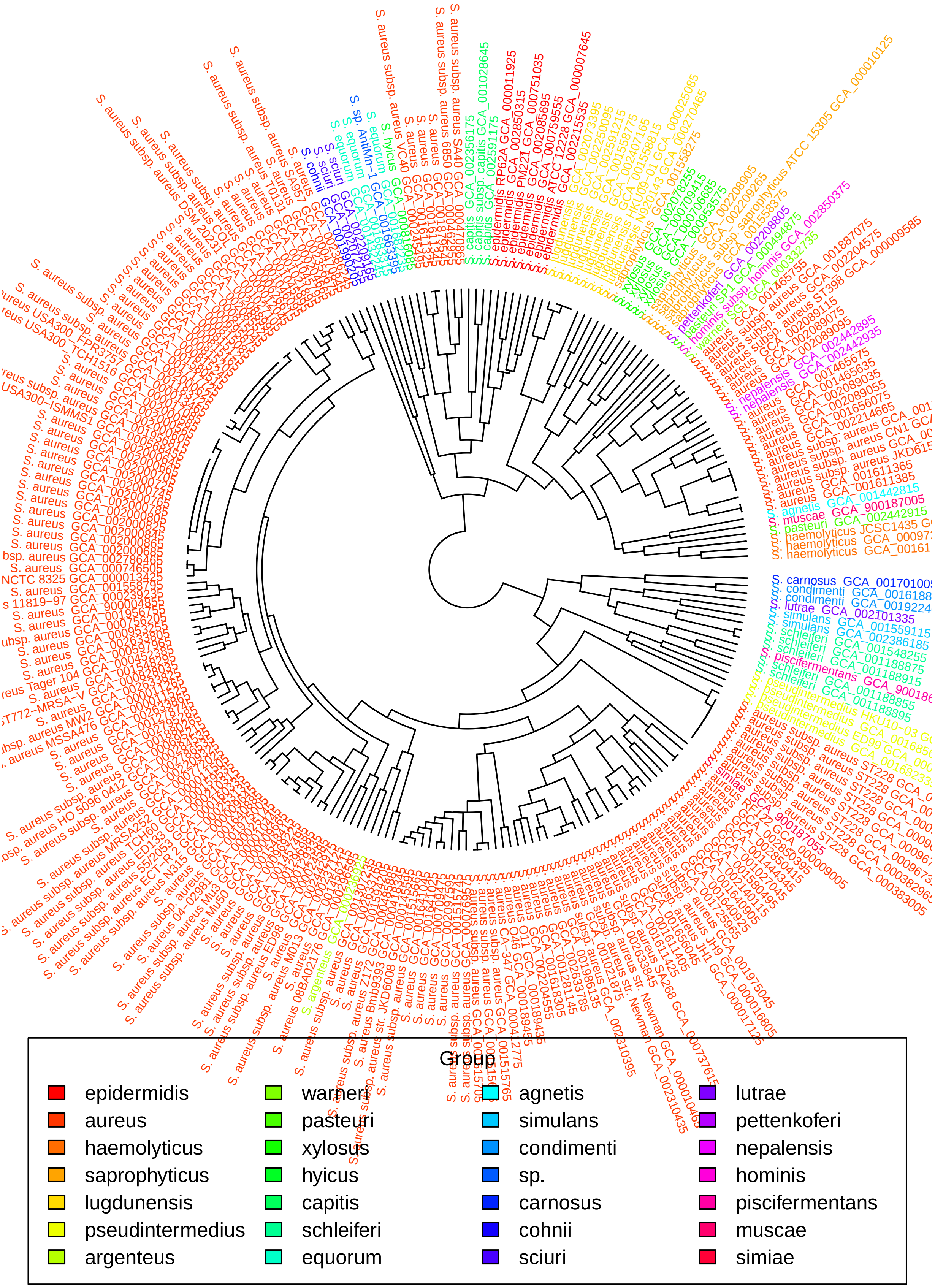

Staphylococcus -----quorum_sensing

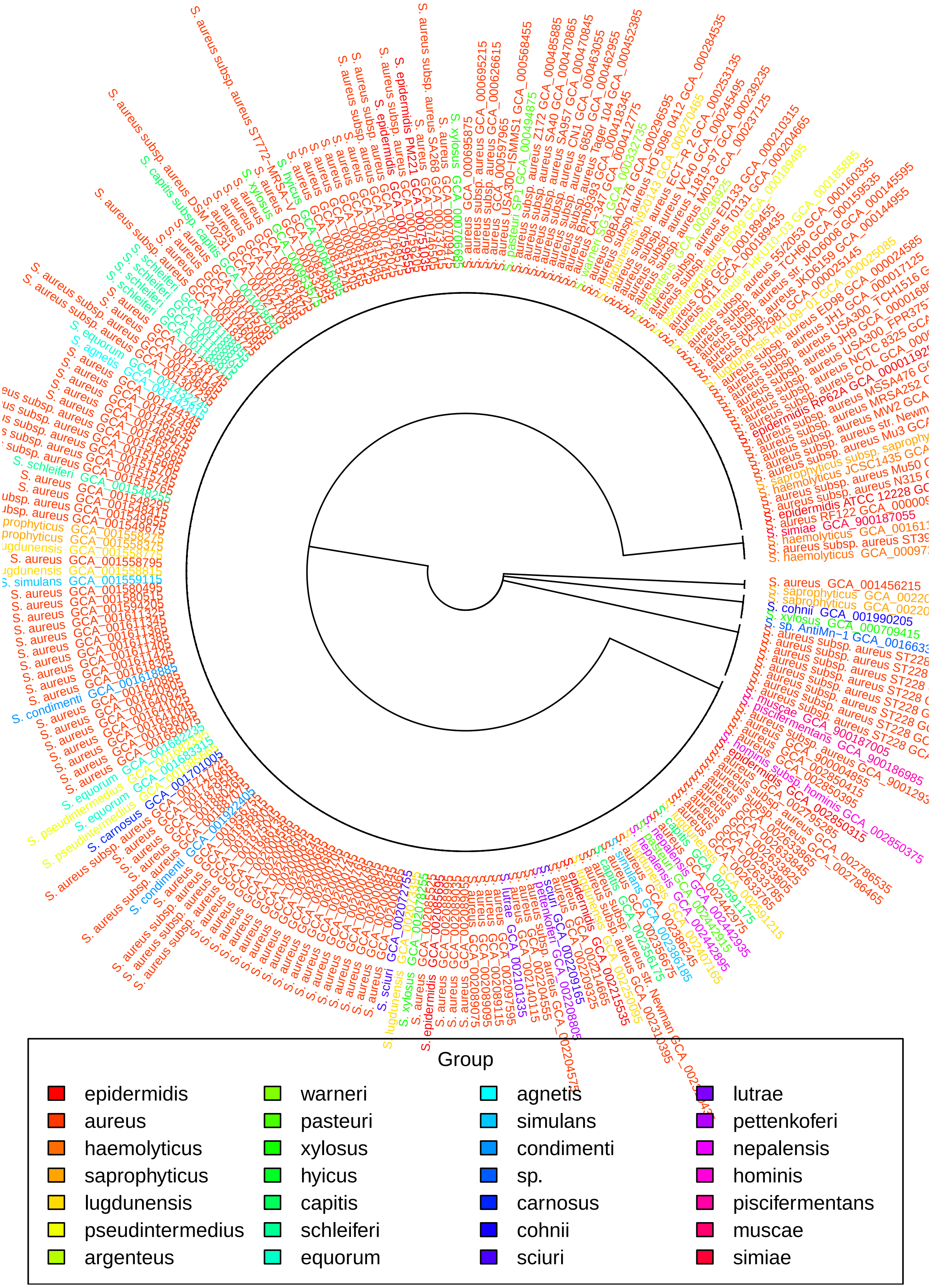

›hylococcus –––modification_of _morphology_or_physiology_of_other_o

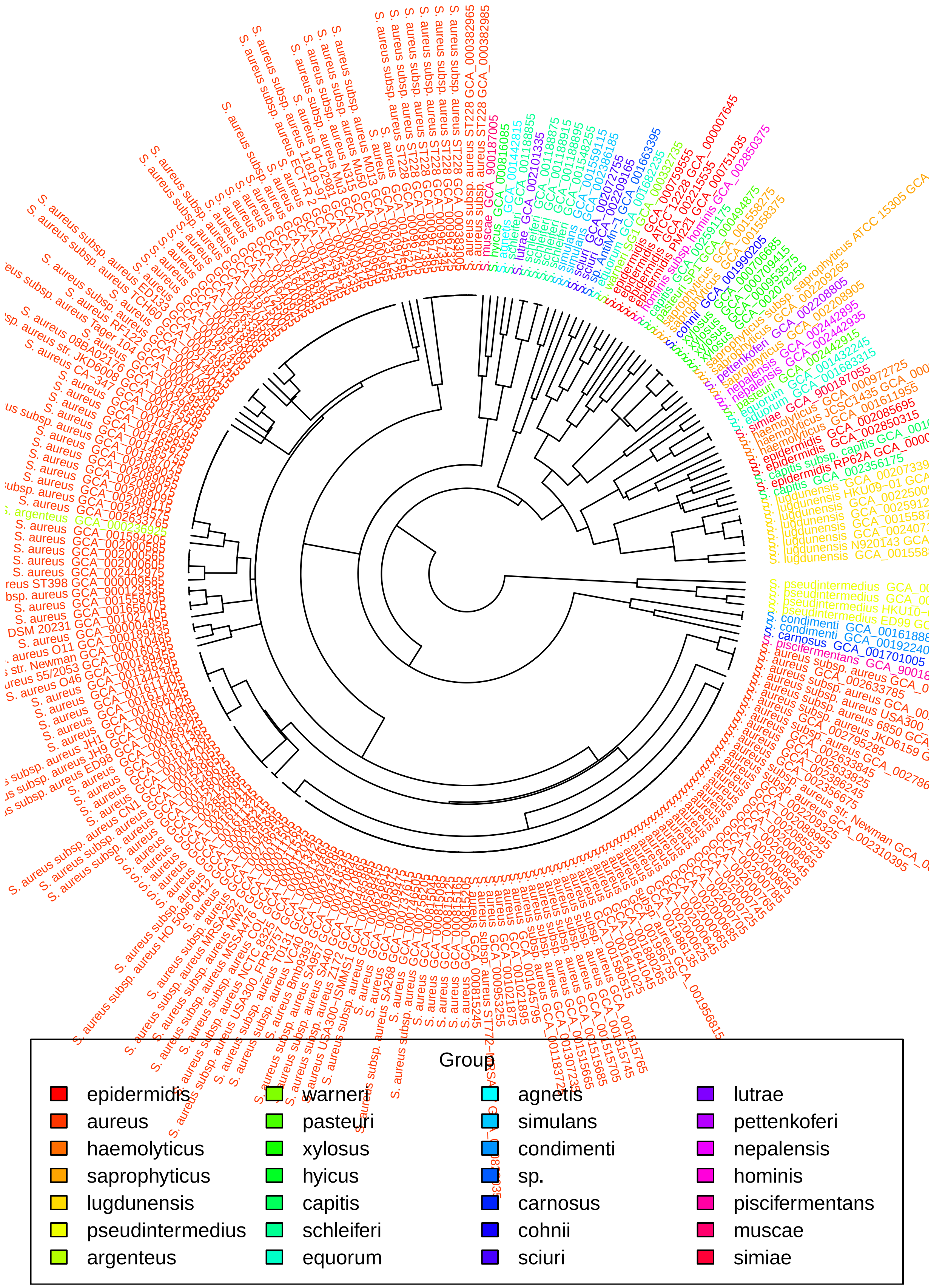

Staphylococcus ---pathogenesis

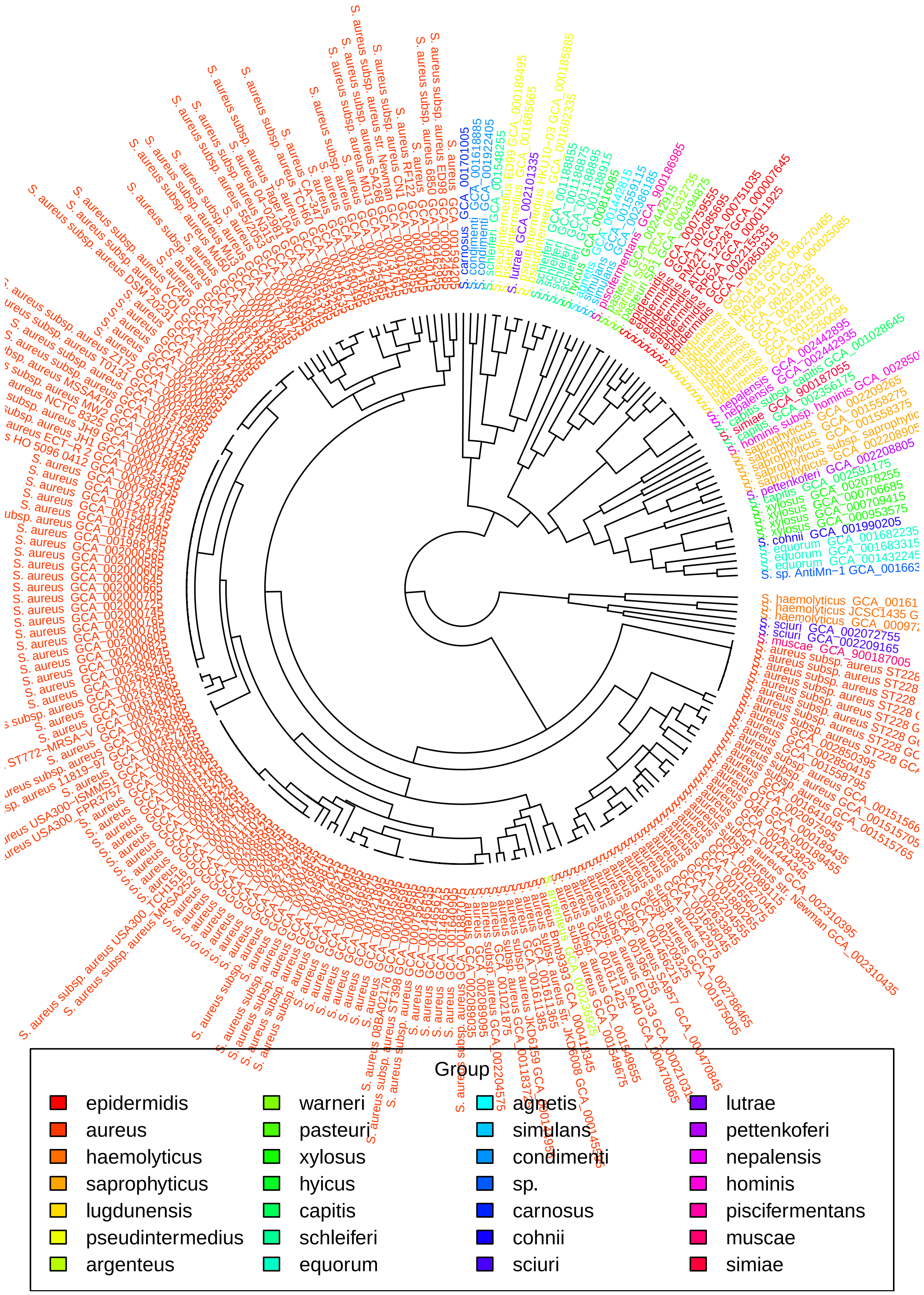

Supplement: Supplementary file 6 — Additional file 6. Staphylococcus Functional trees [file 12864_2021_7388_MOESM6_ESM.pdf]
