# Supplementary material for: Phenotype and multi-omics comparison of Staphylococcus and Streptococcus uncovers pathogenic traits and predicts zoonotic potential
Source: BMC Genomics. 2021 Feb 4;22:102. doi: 10.1186/s12864-021-07388-6 (PMC7860044; doi:10.1186/s12864-021-07388-6)

All proteins

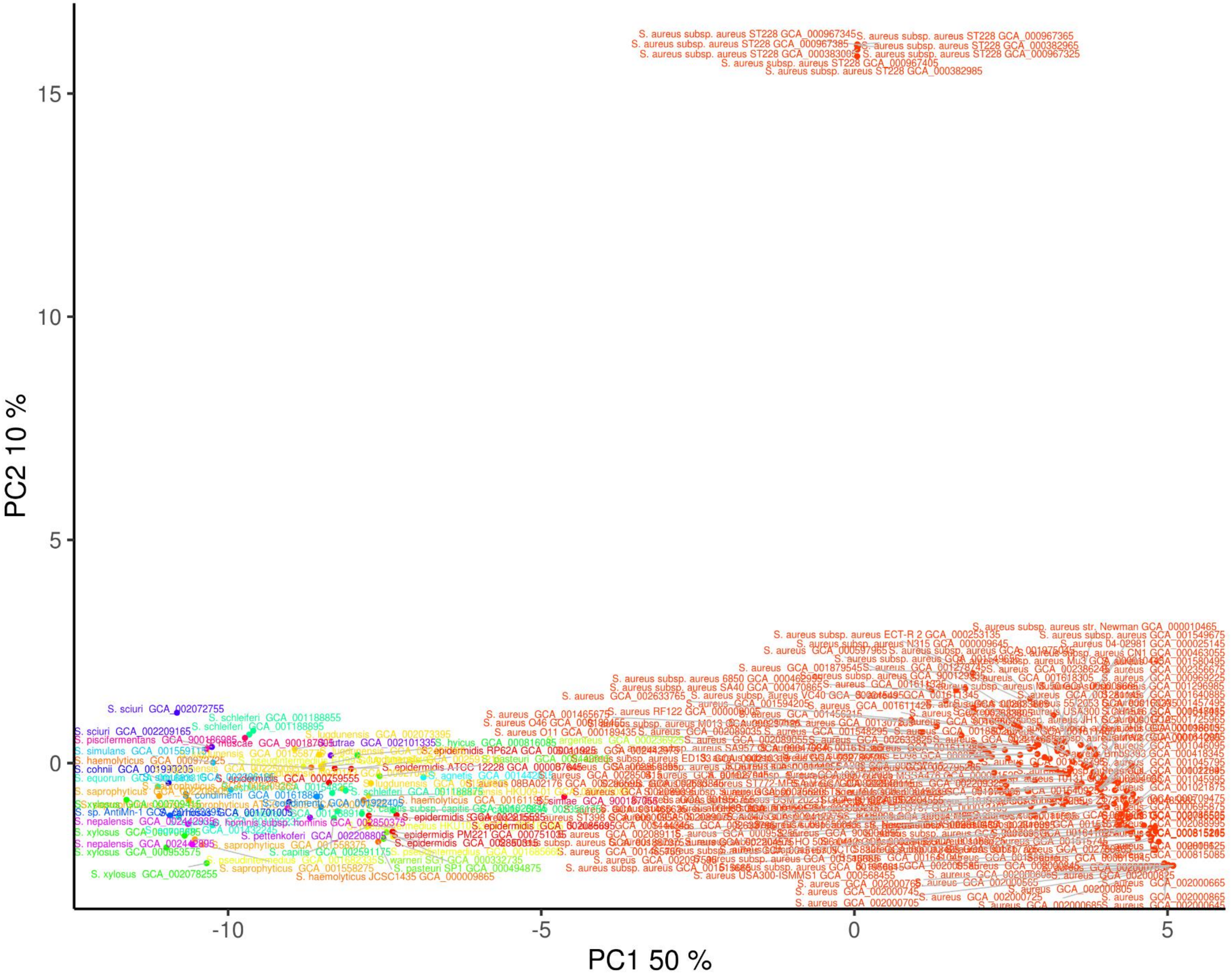

All proteins with GO annotation

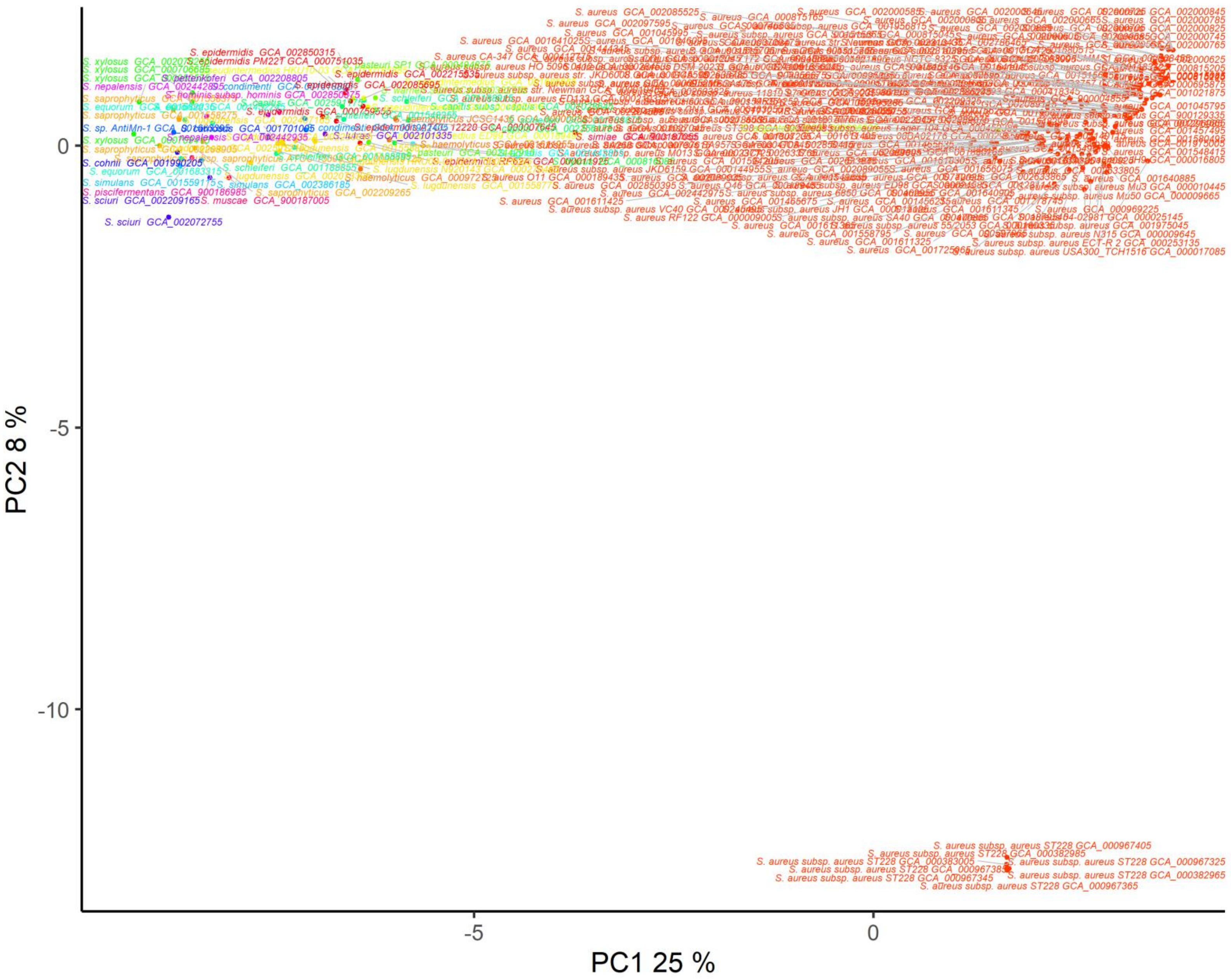

GO:0008150 Biological process

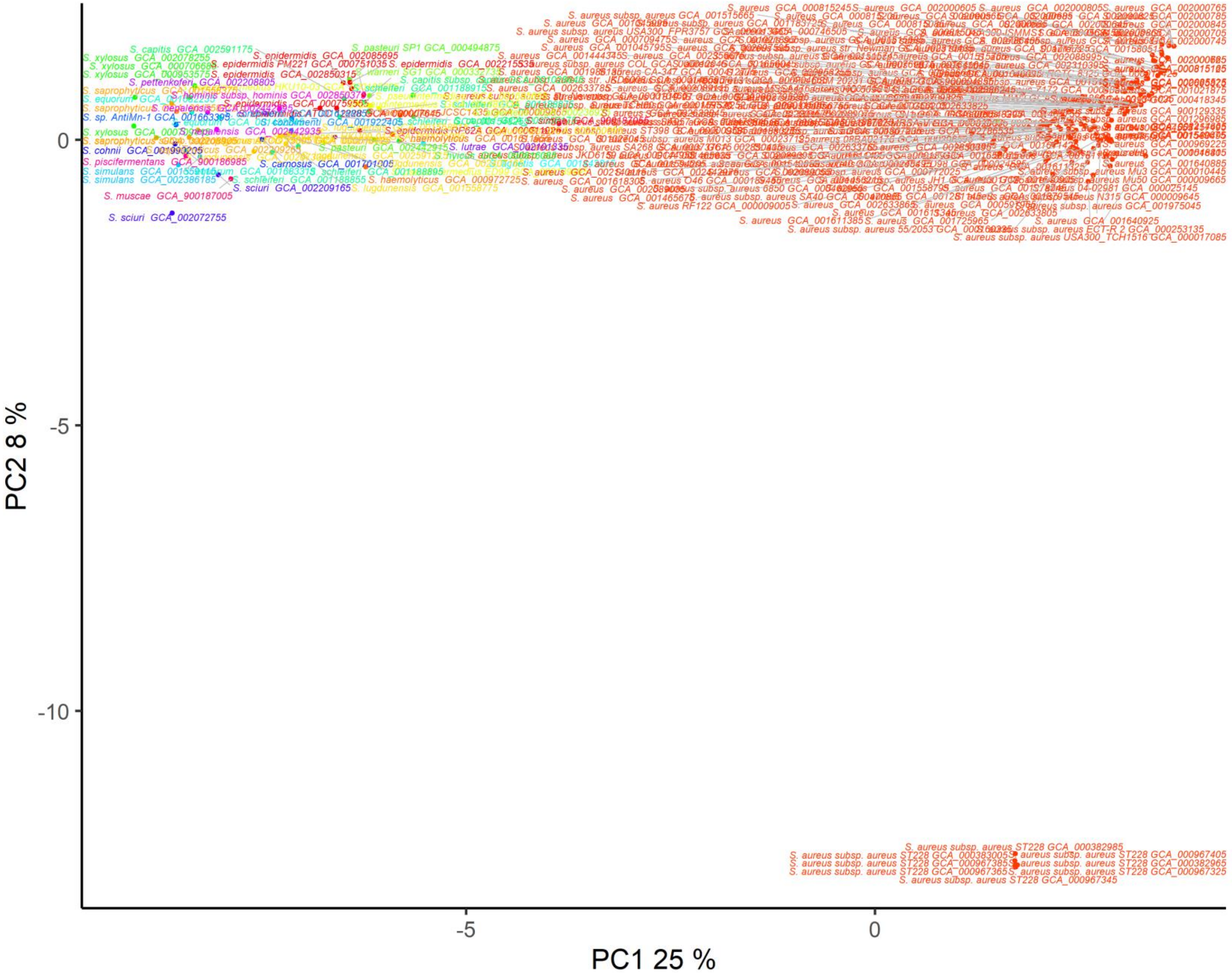

GO:0008152 *Metabolic process

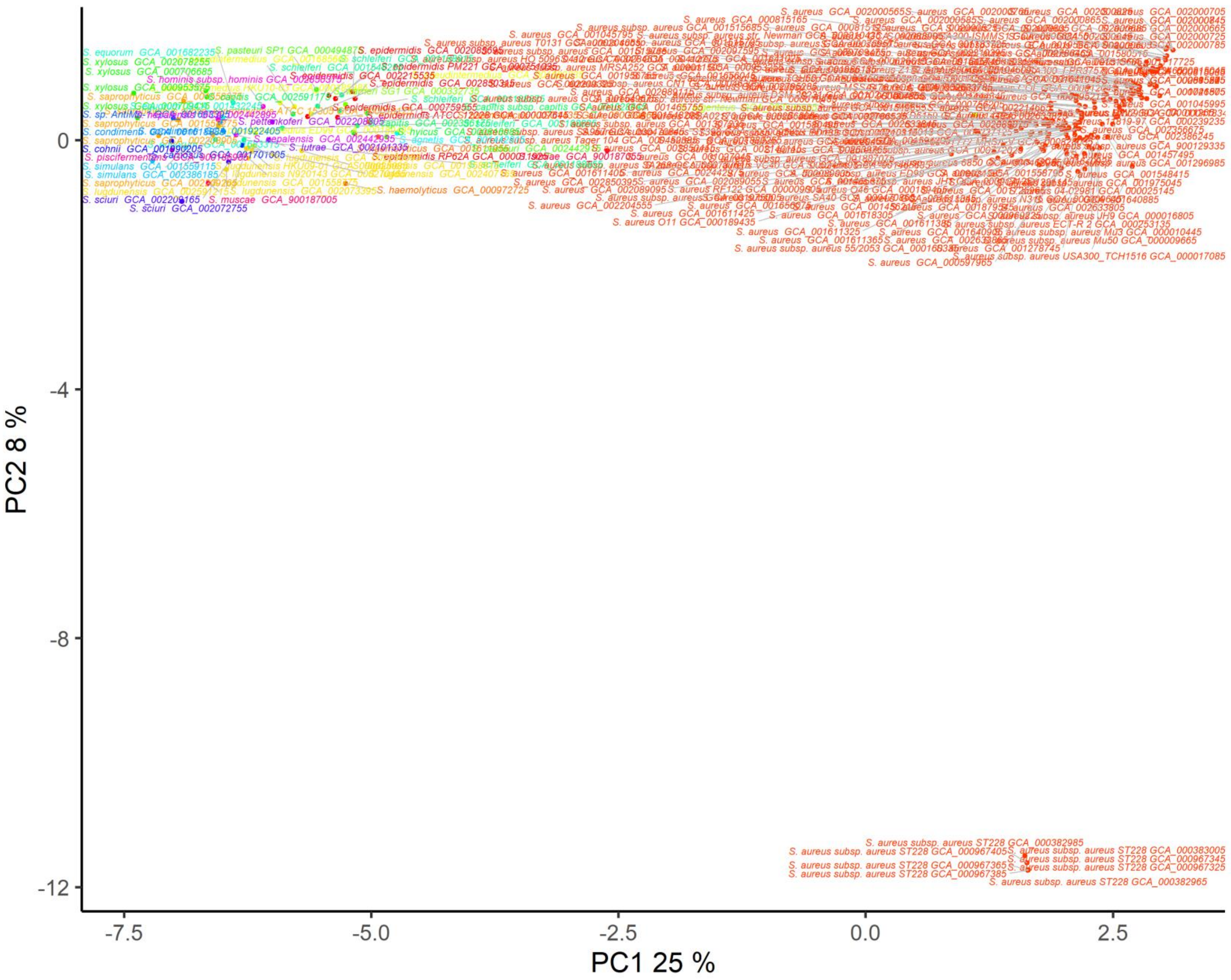

GO:0017144 Drug metabolic process

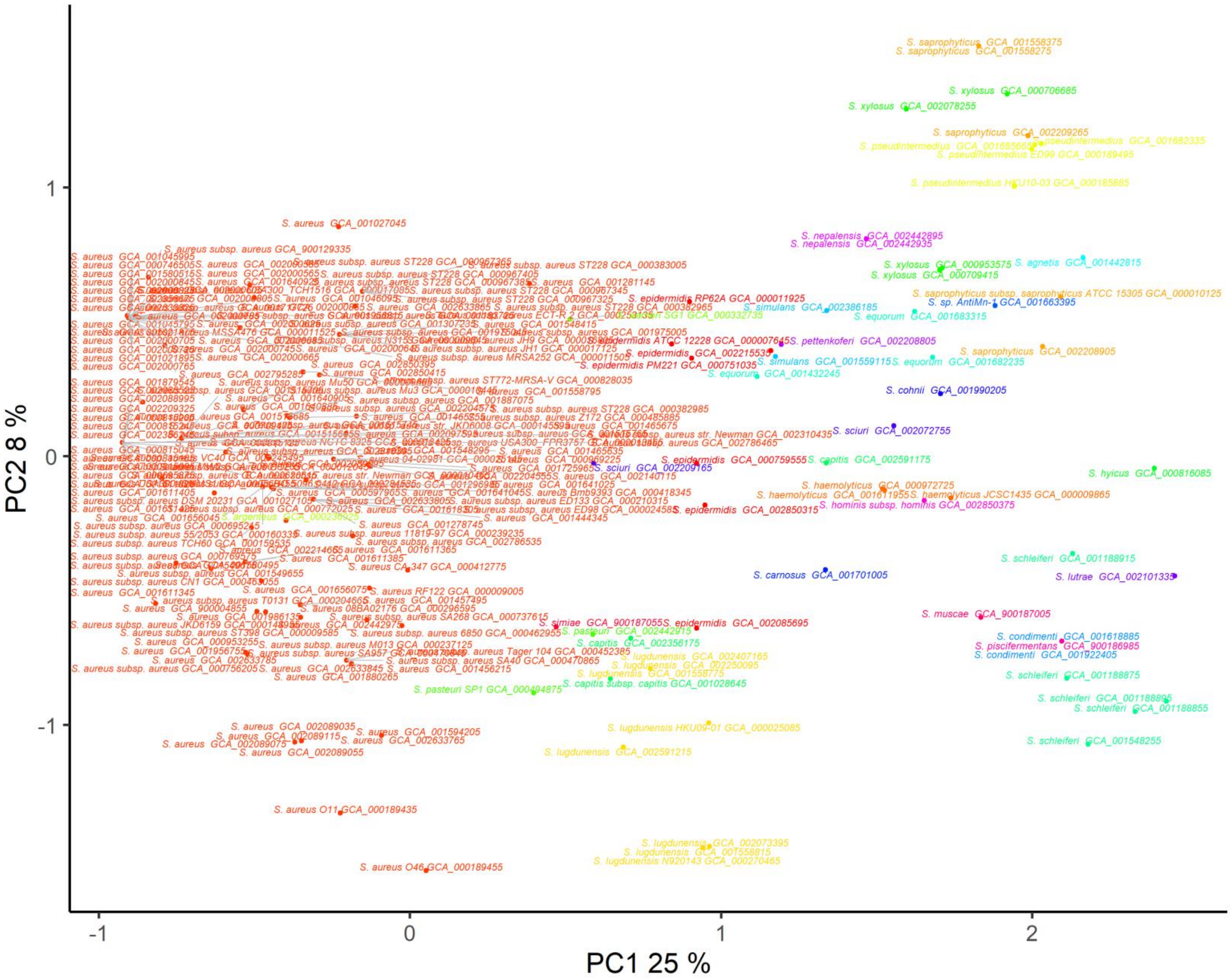

GO:0042493 Response to drug

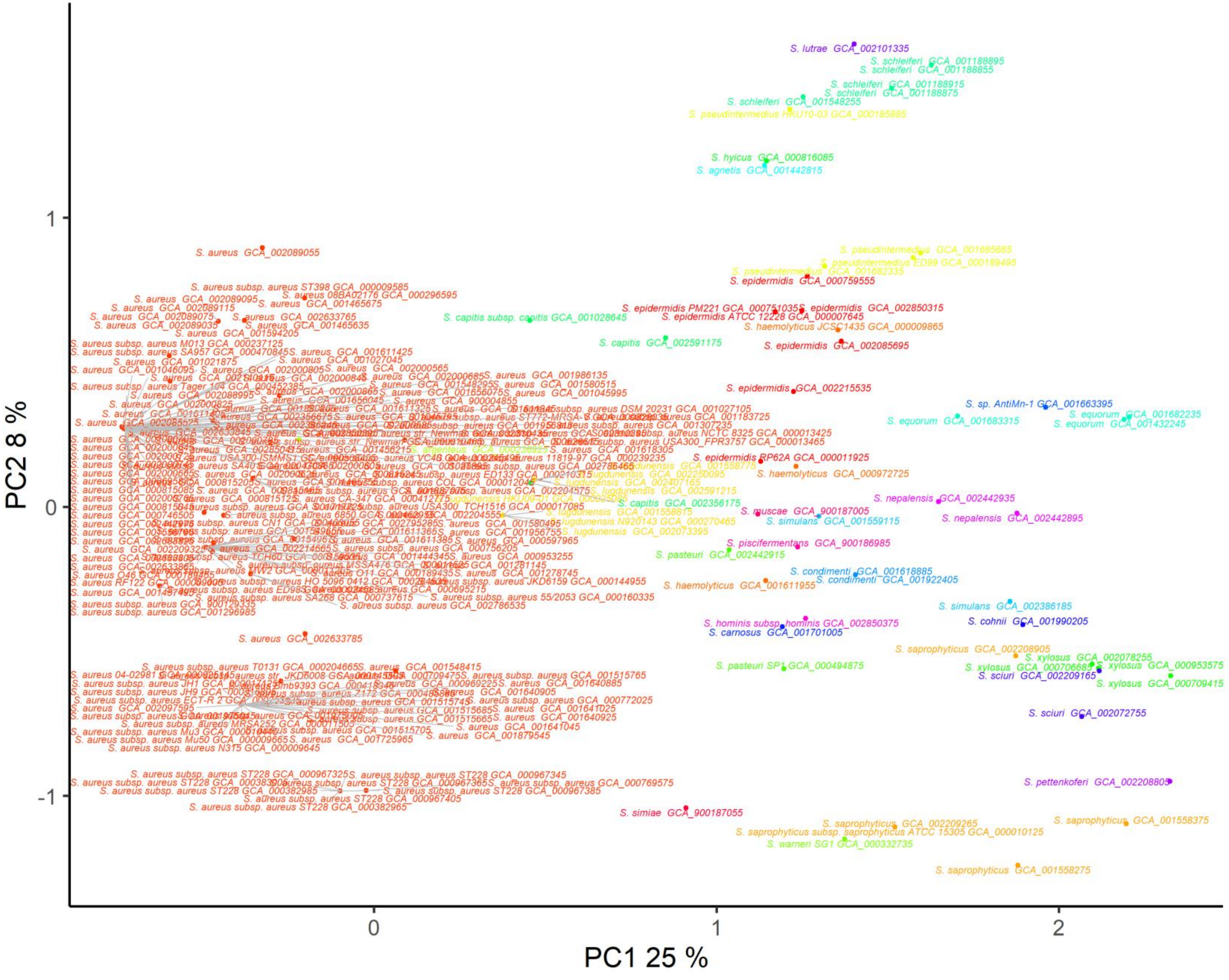

GO:0023052 *Signalling

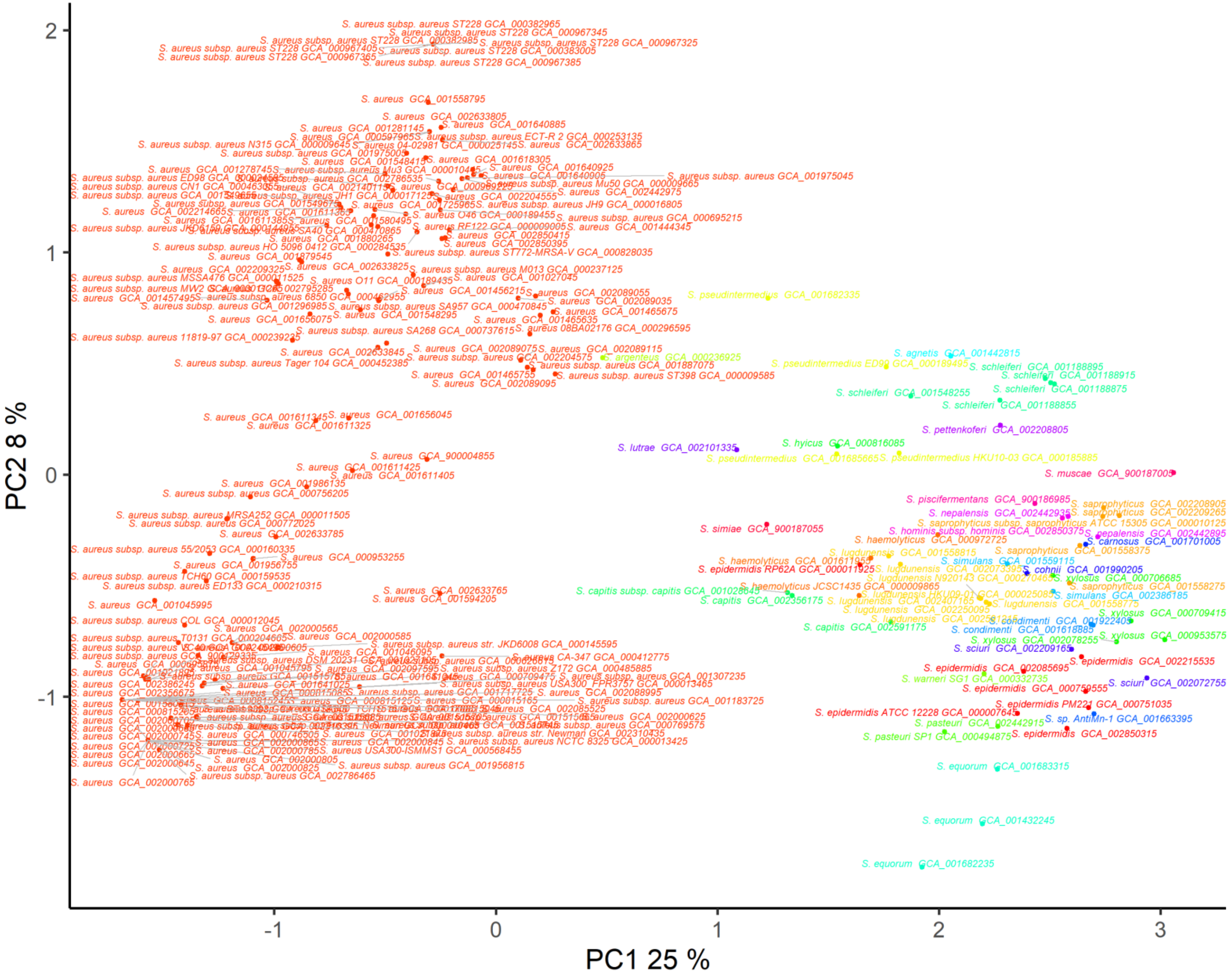

GO:0065007 *Biological regulation

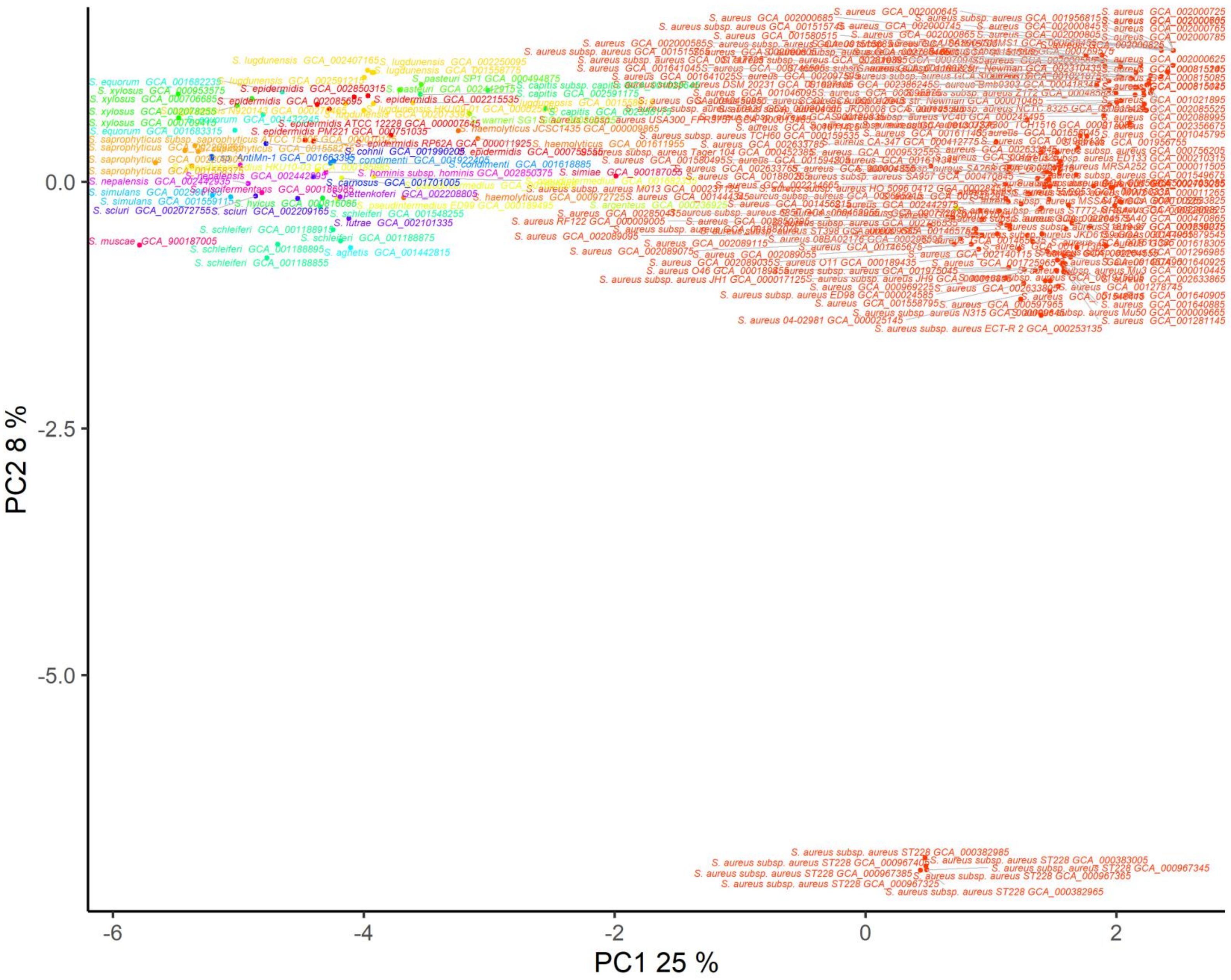

GO:0022610 *Biological adhesion

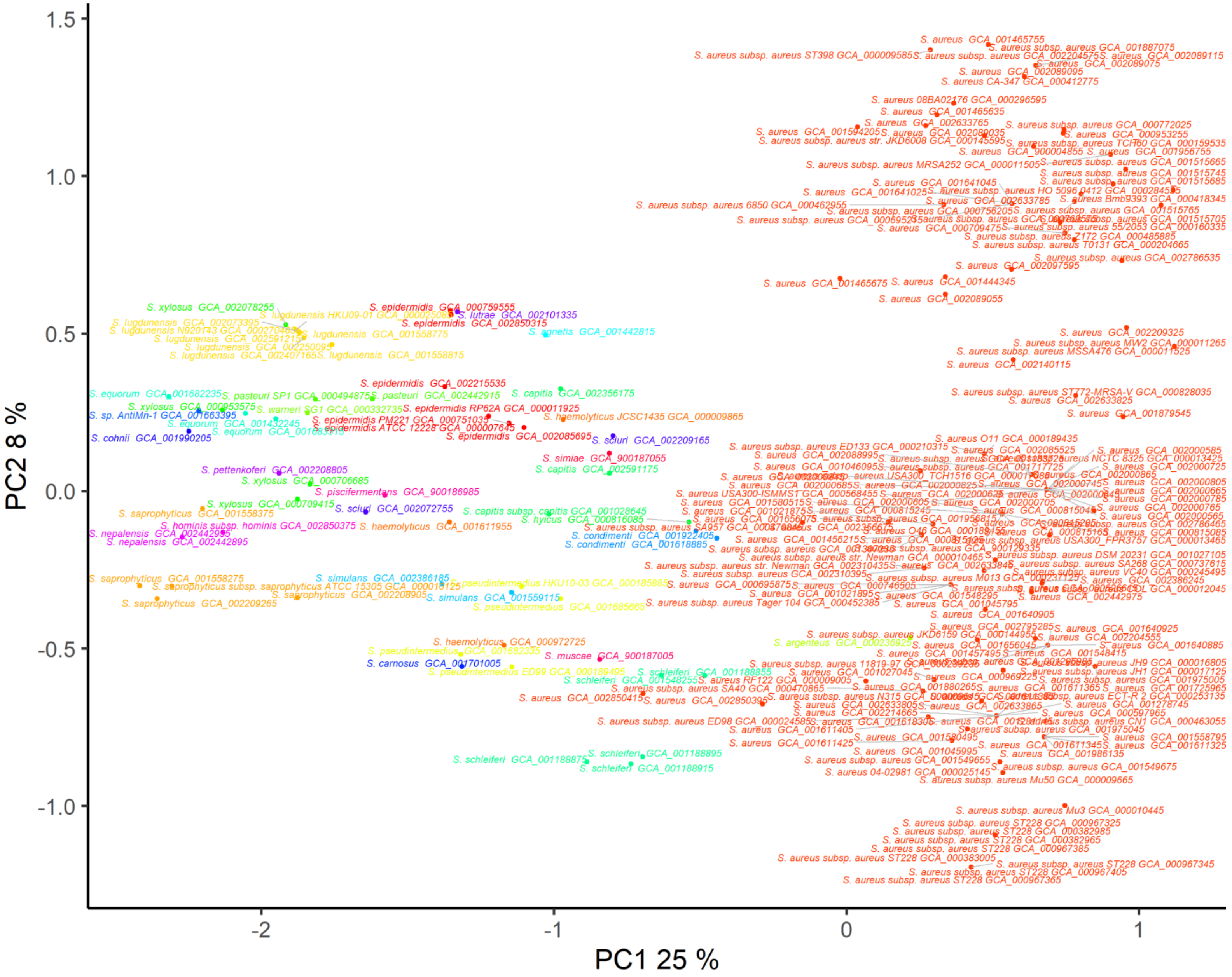

GO:0044419 Inter species interaction between organisms

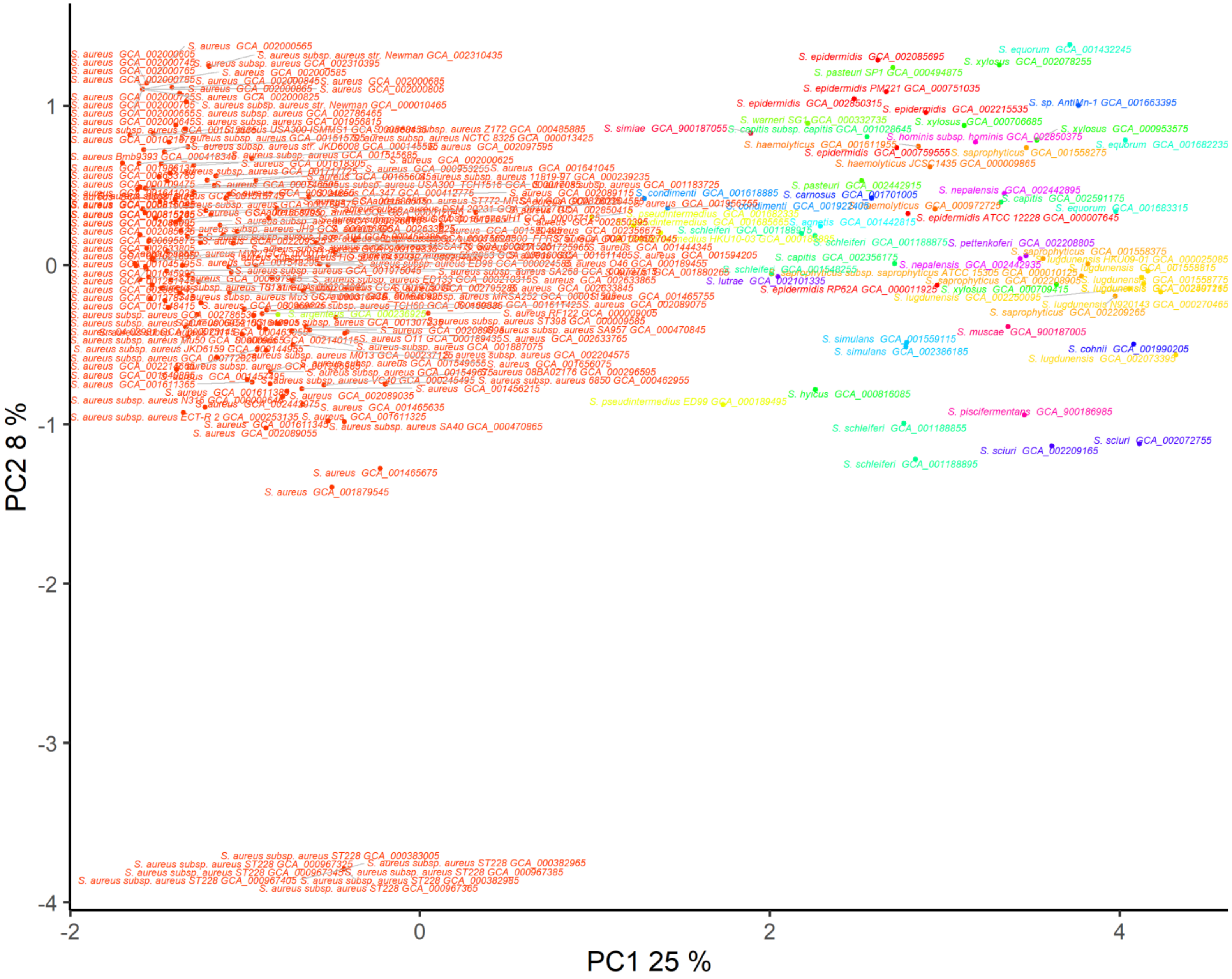

GO:0042710 Biofilm formation

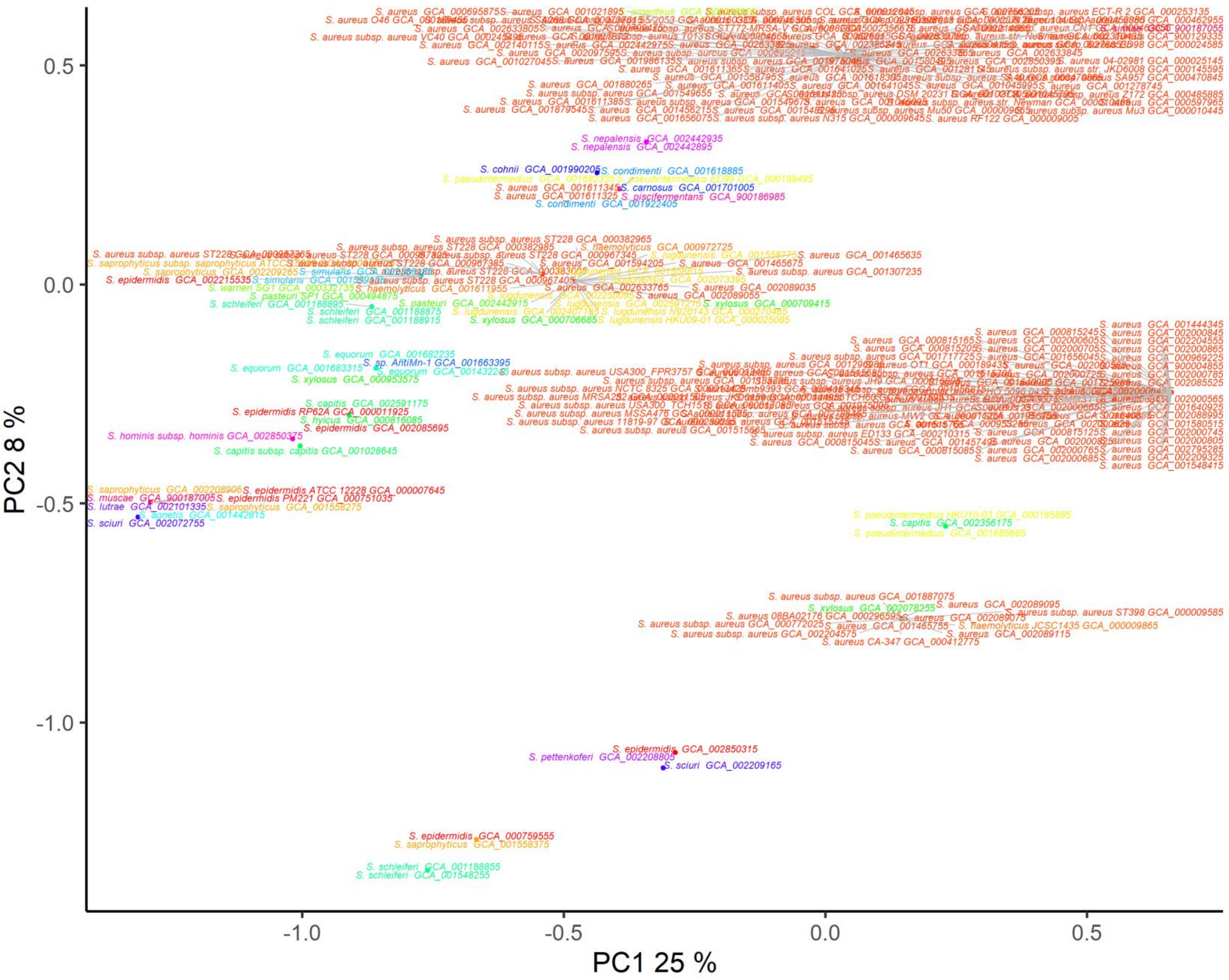

GO:0098743 Cell aggregation

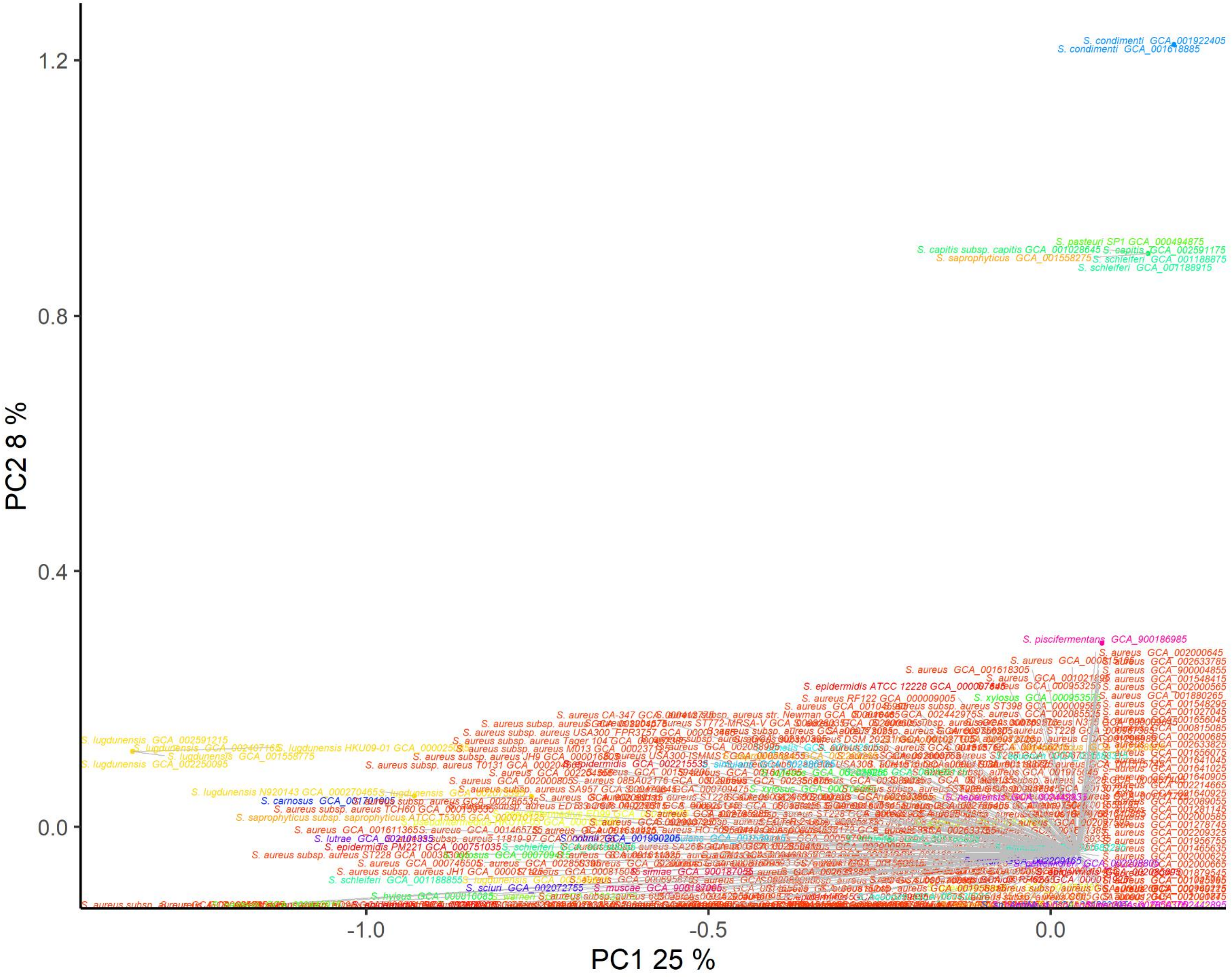

GO:0044403 Symbiont process

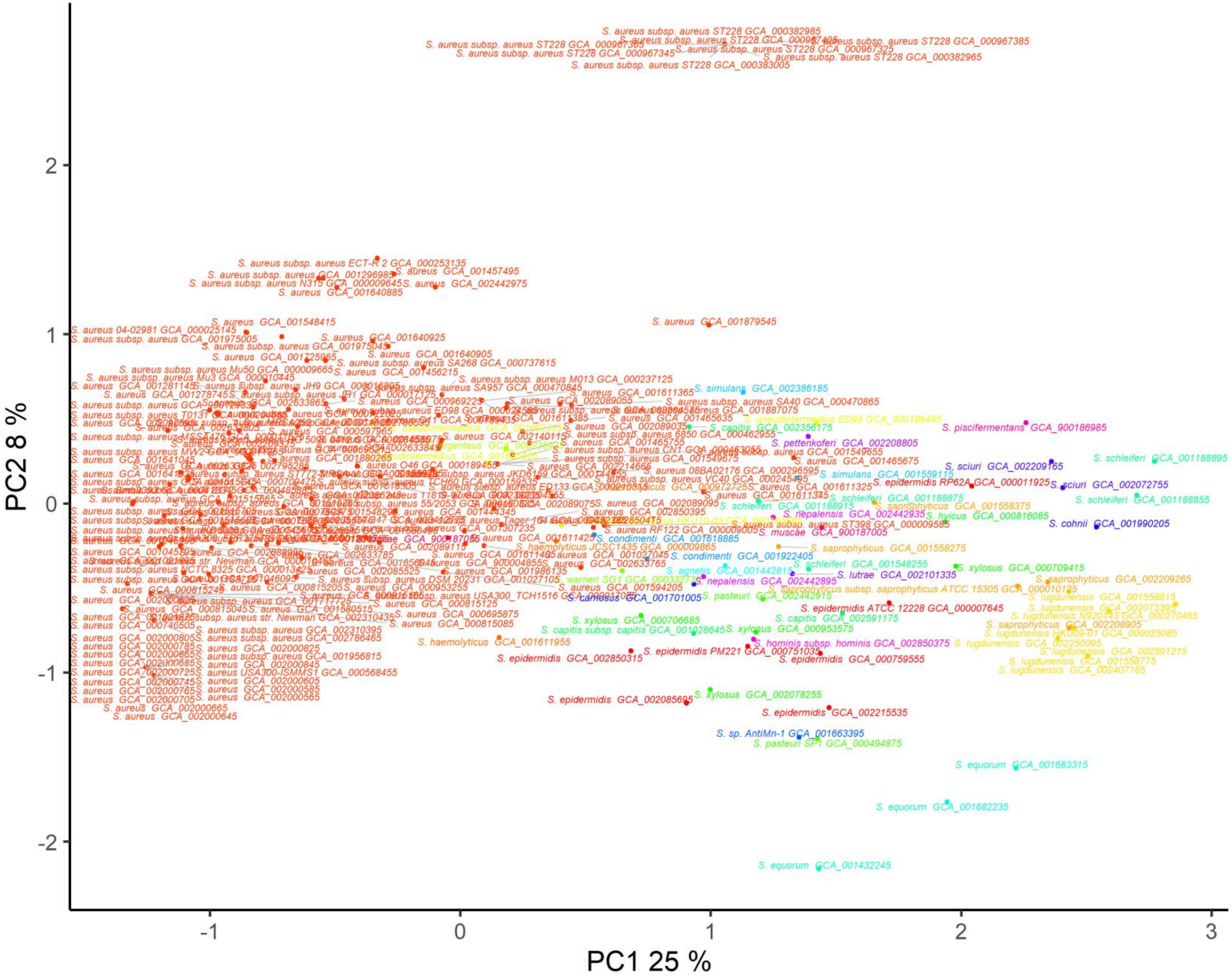

GO:0009372 Quorum sensing

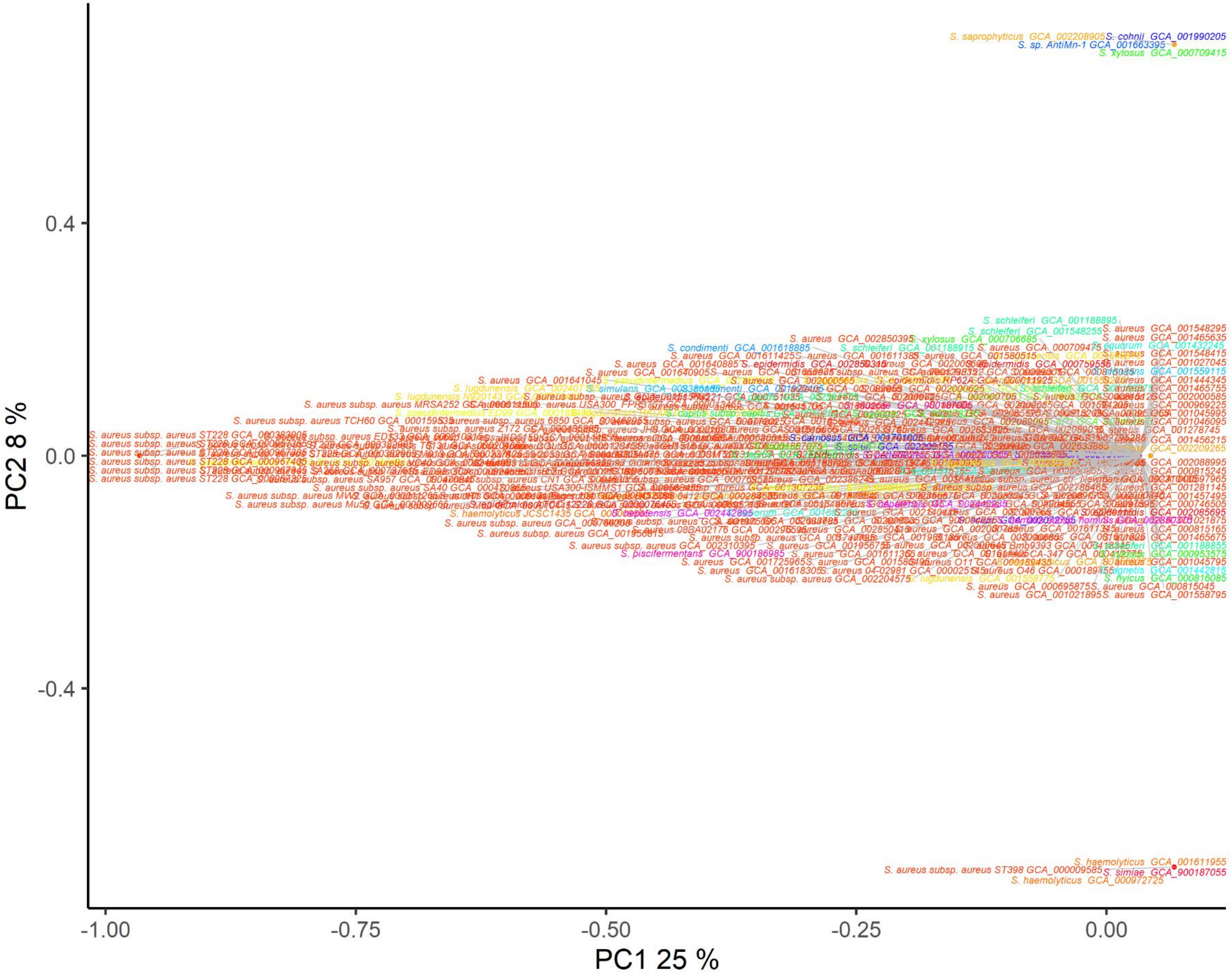

GO:0035821 Modification of morphology or physiology of other organism

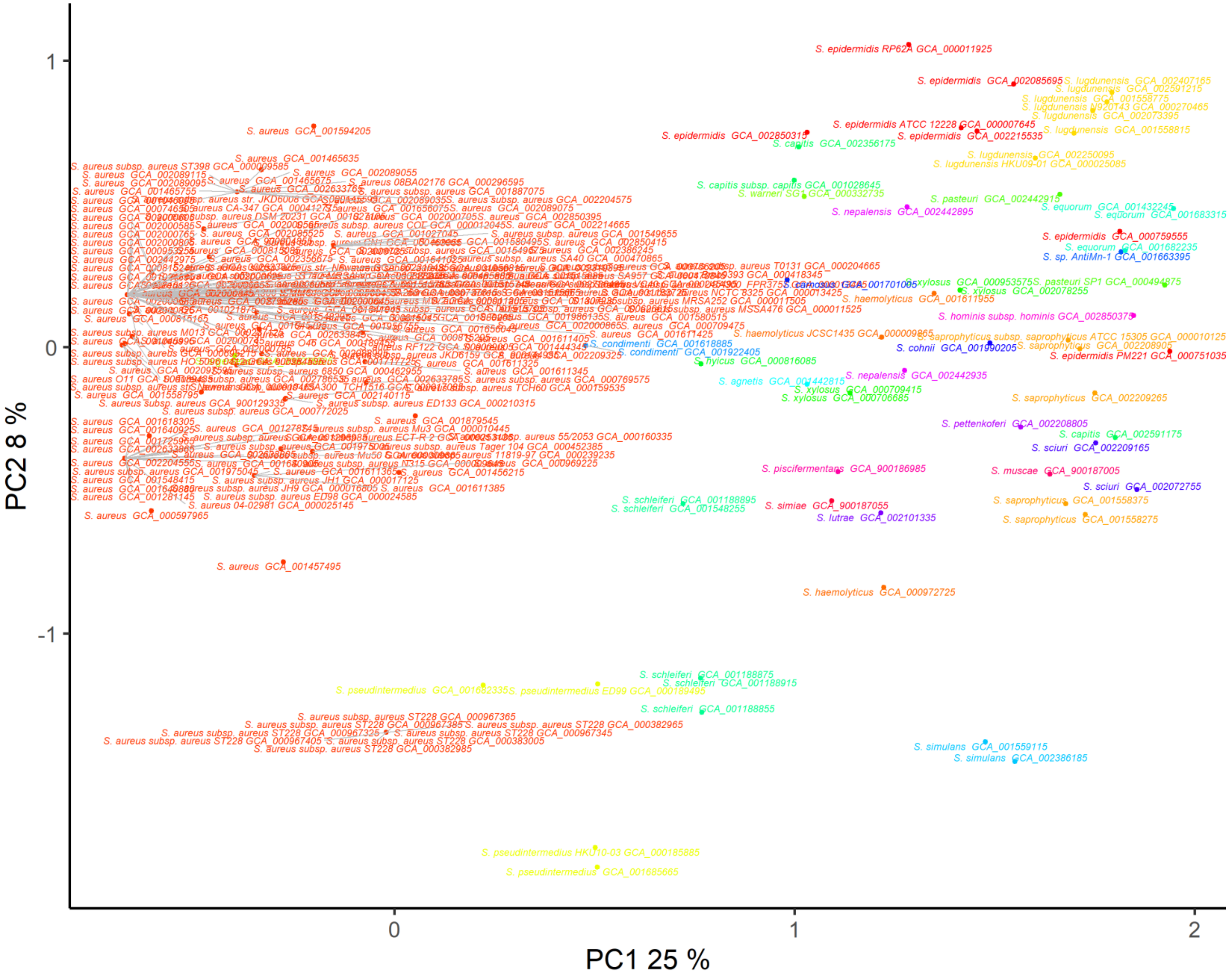

GO:0009405 Pathogenesis

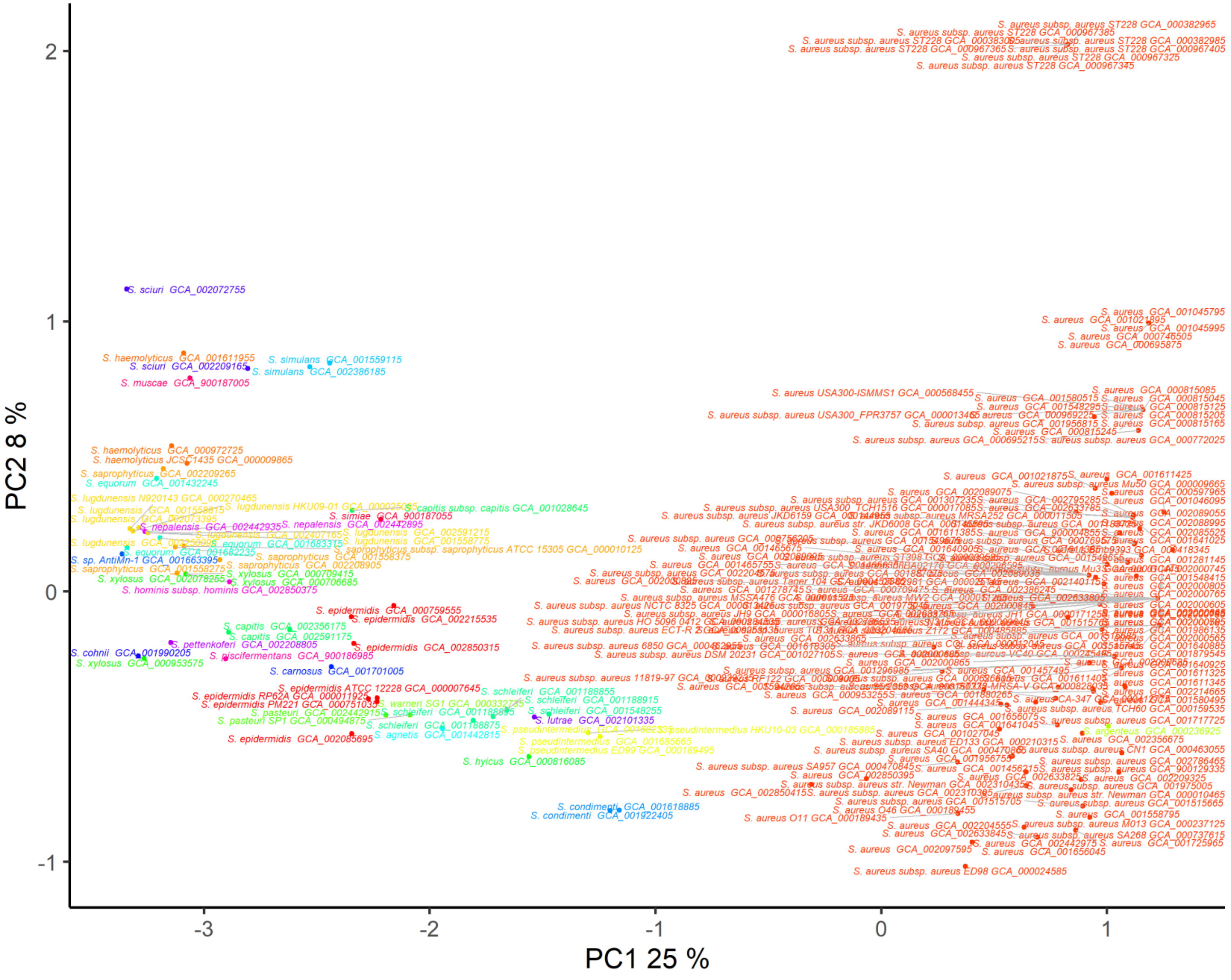

Supplement: Supplementary file 7 — Additional file 7. Staphylococcus PCA [file 12864_2021_7388_MOESM7_ESM.pdf]
