# Supplementary material for: Phenotype and multi-omics comparison of Staphylococcus and Streptococcus uncovers pathogenic traits and predicts zoonotic potential
Source: BMC Genomics. 2021 Feb 4;22:102. doi: 10.1186/s12864-021-07388-6 (PMC7860044; doi:10.1186/s12864-021-07388-6)
Supplement: Supplementary file 9 — Additional file 9. Streptococcus Functional trees [file 12864_2021_7388_MOESM9_ESM.pdf]

Streptococcus All architectures

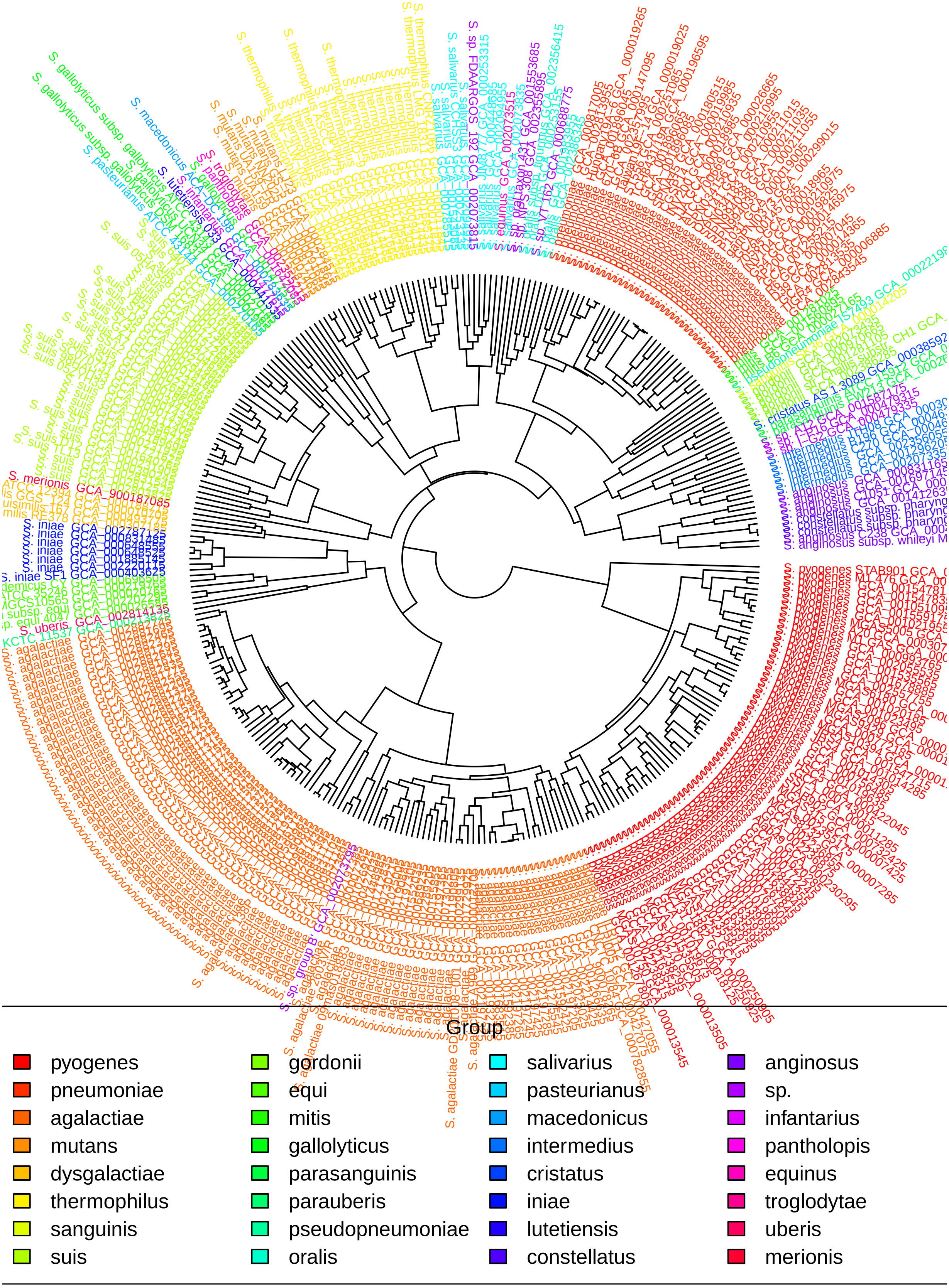

Streptococcus All GO

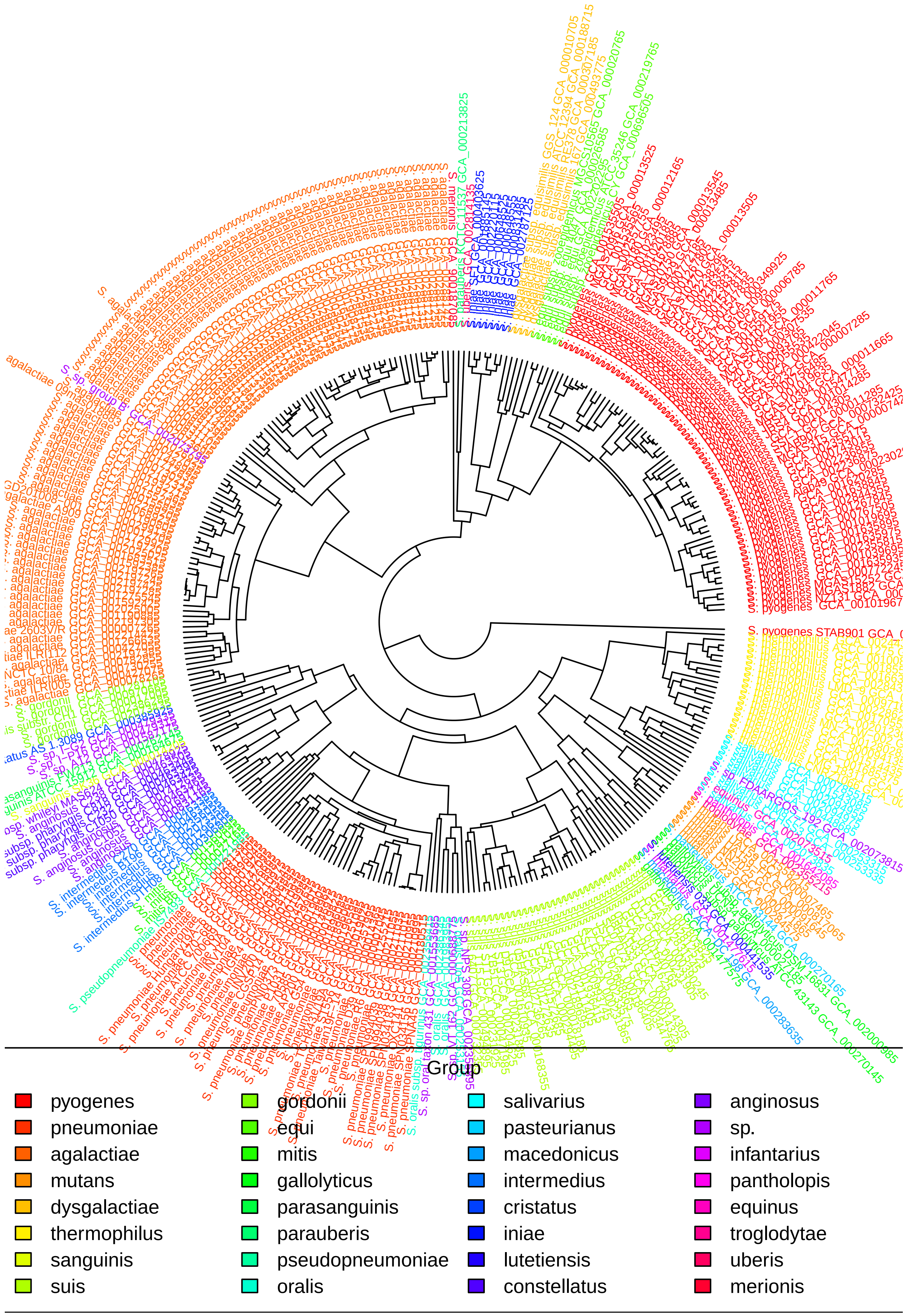

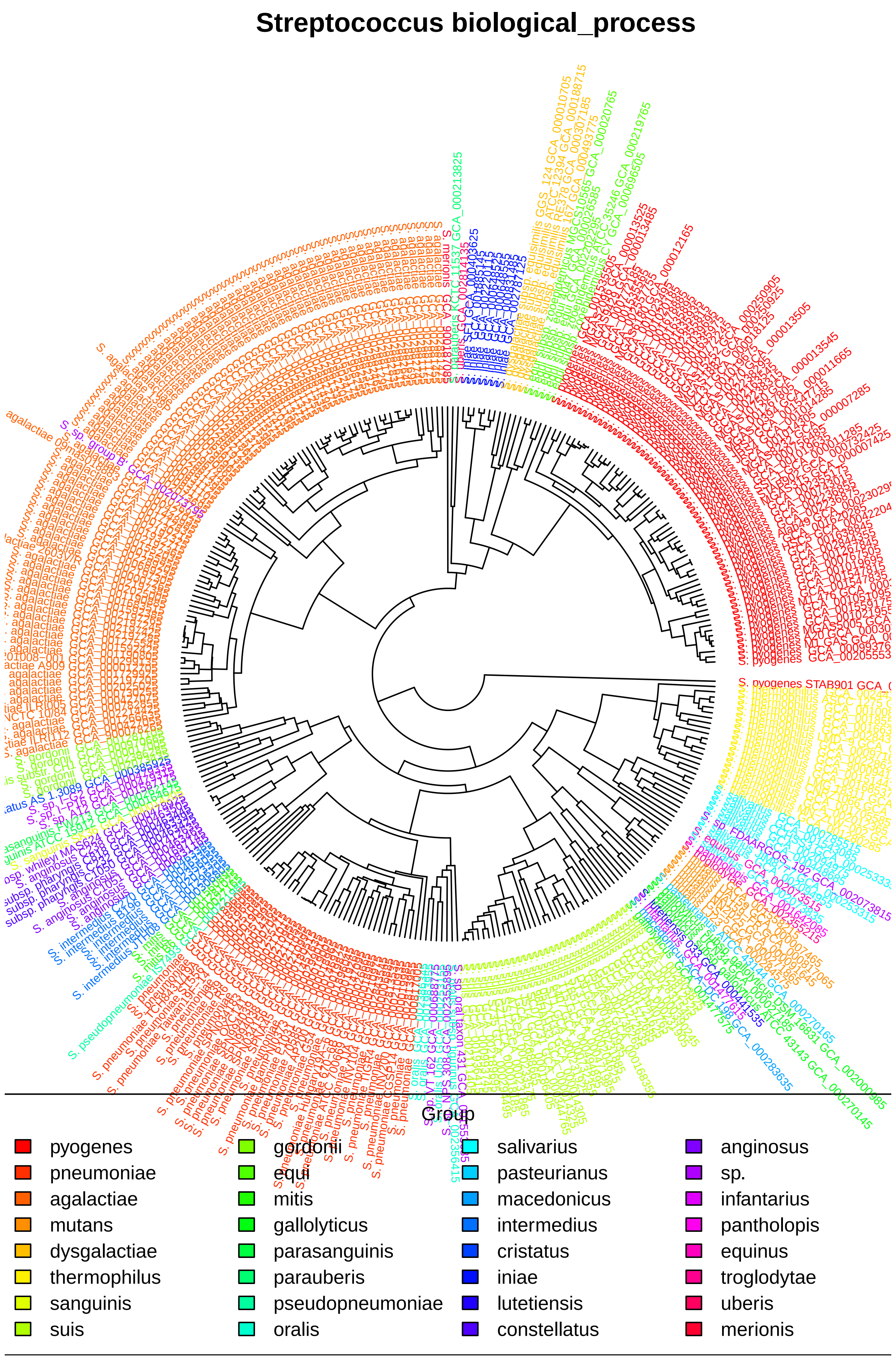
Streptococcus biological_process
Group
pyogenes
pneumoniae
agalactiae
mutans
dysgalactiae
thermophilus
sanguinis
suis
gordonii
equi
mitis
gallolyticus
parasanguinis
parauberis
pseudopneumoniae
oralis
salivarius
pasteurianus
macedonicus
intermedius
cristatus
iniae
lutetiensis
constellatus
anginosus
sp.
infantarius
pantholopis
equinus
troglodytae
uberis
merionis

Streptococcus metabolic_process

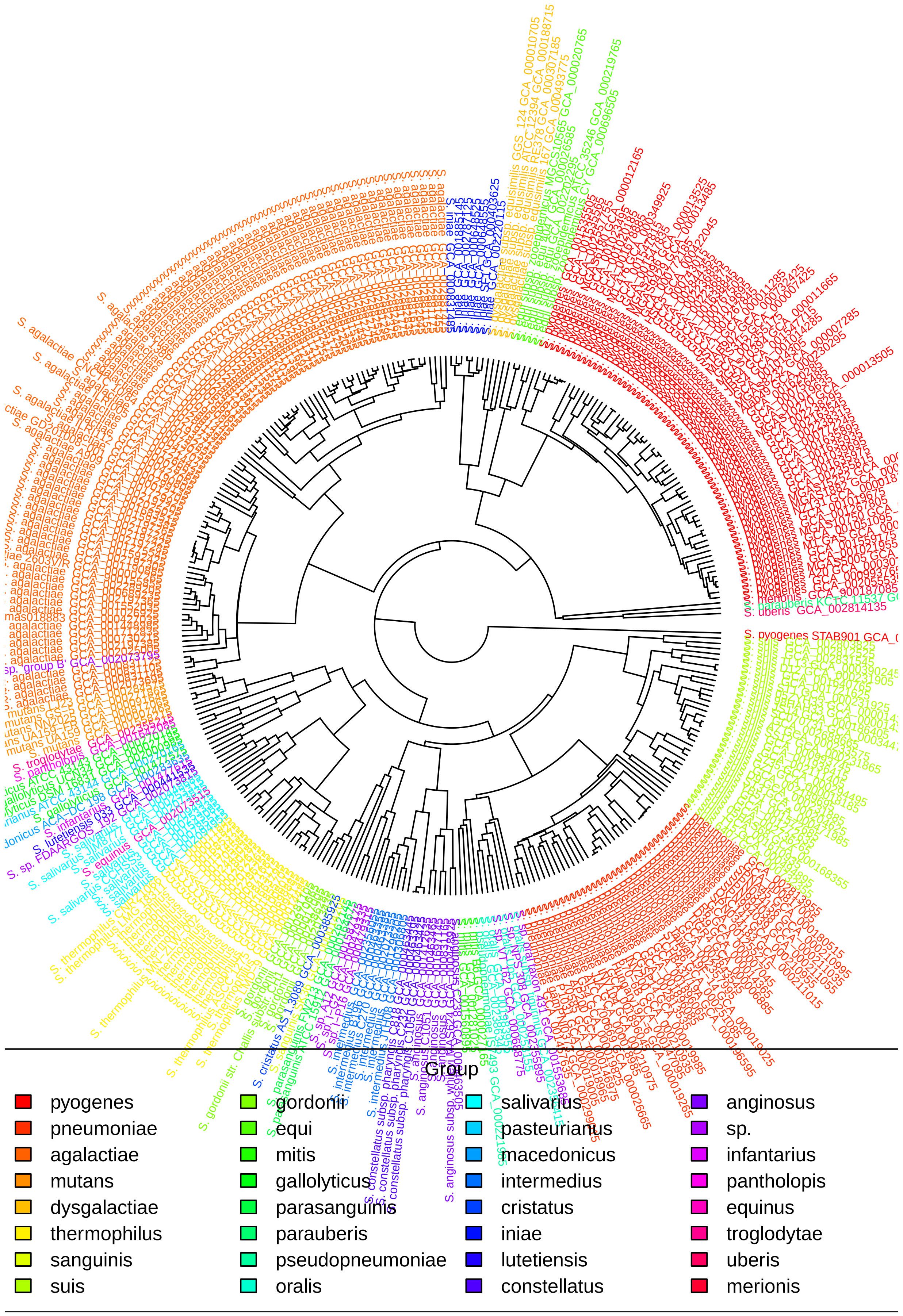

Streptococcus −−drug_metabolic_process

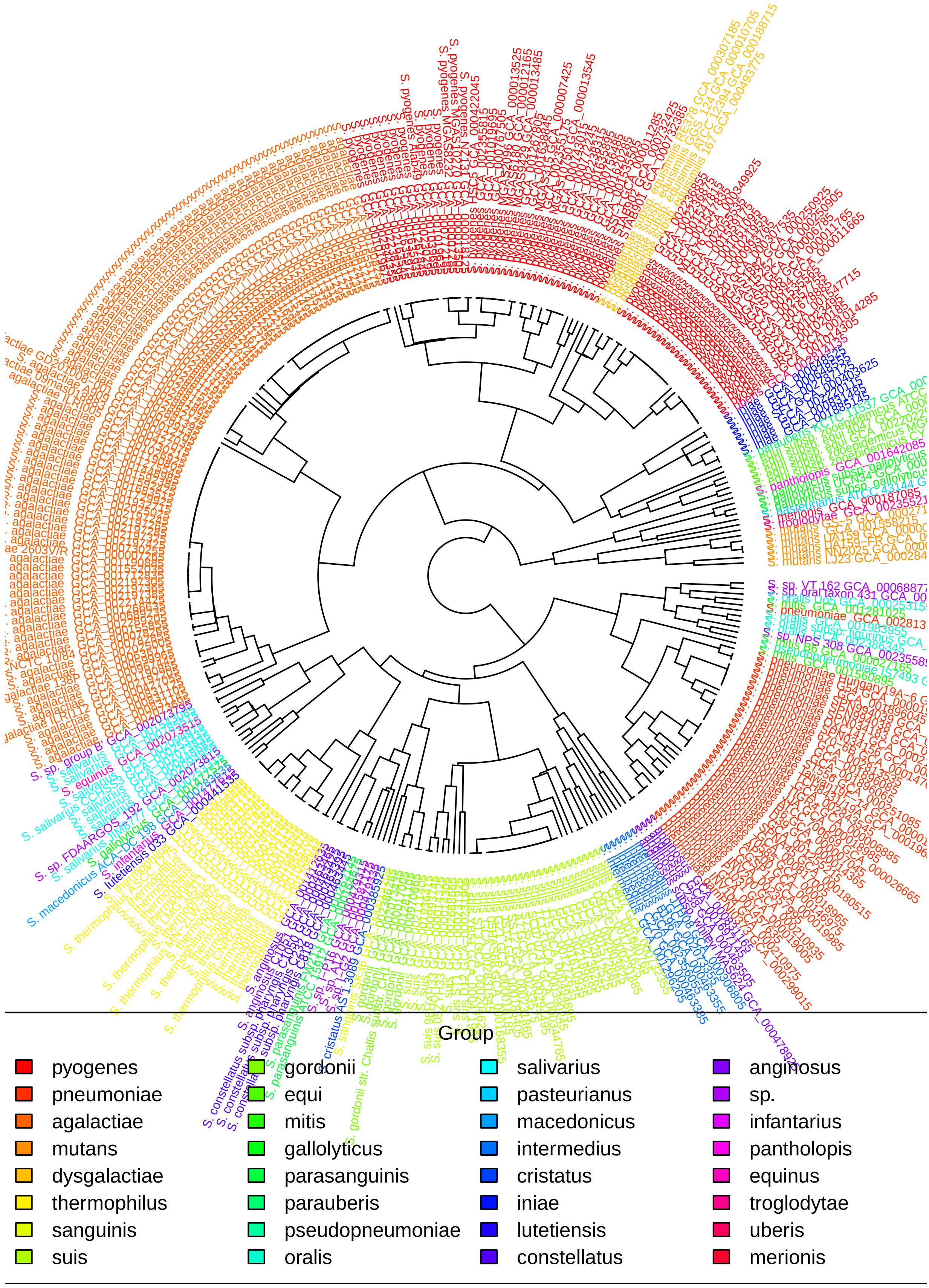

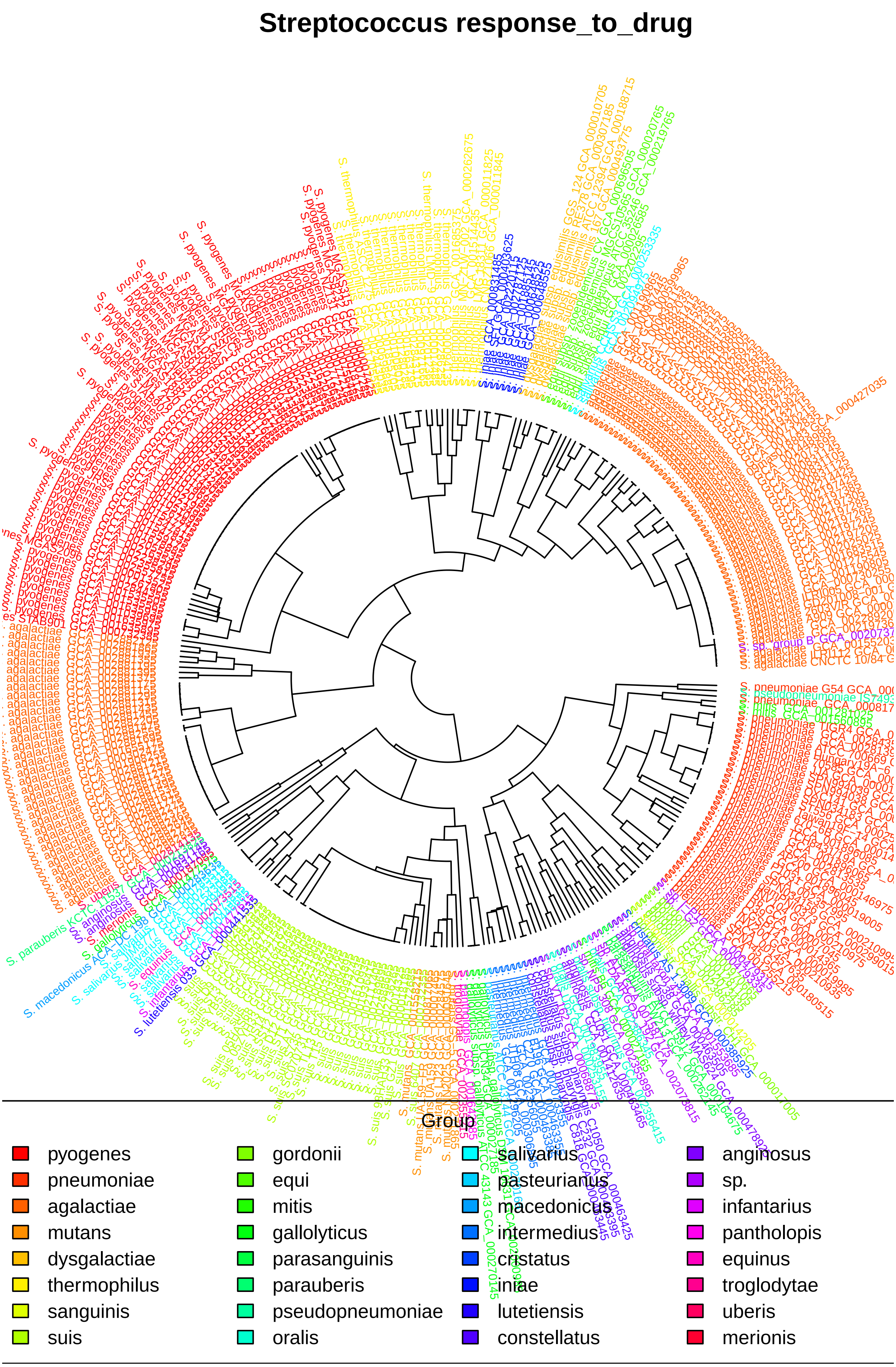
Streptococcus response_to_drug
Group
pyogenes
pneumoniae
agalactiae
mutans
dysgalactiae
thermophilus
sanguinis
suis
gordonii
equi
mitis
gallolyticus
parasanguinis
parauberis
pseudopneumoniae
oralis
salivarius
pasteurianus
macedonicus
intermedius
cristatus
iniae
lutetiensis
constellatus
anginosus
sp.
infantarius
pantholopis
equinus
troglodytae
uberis
merionis

Streptococcus signaling

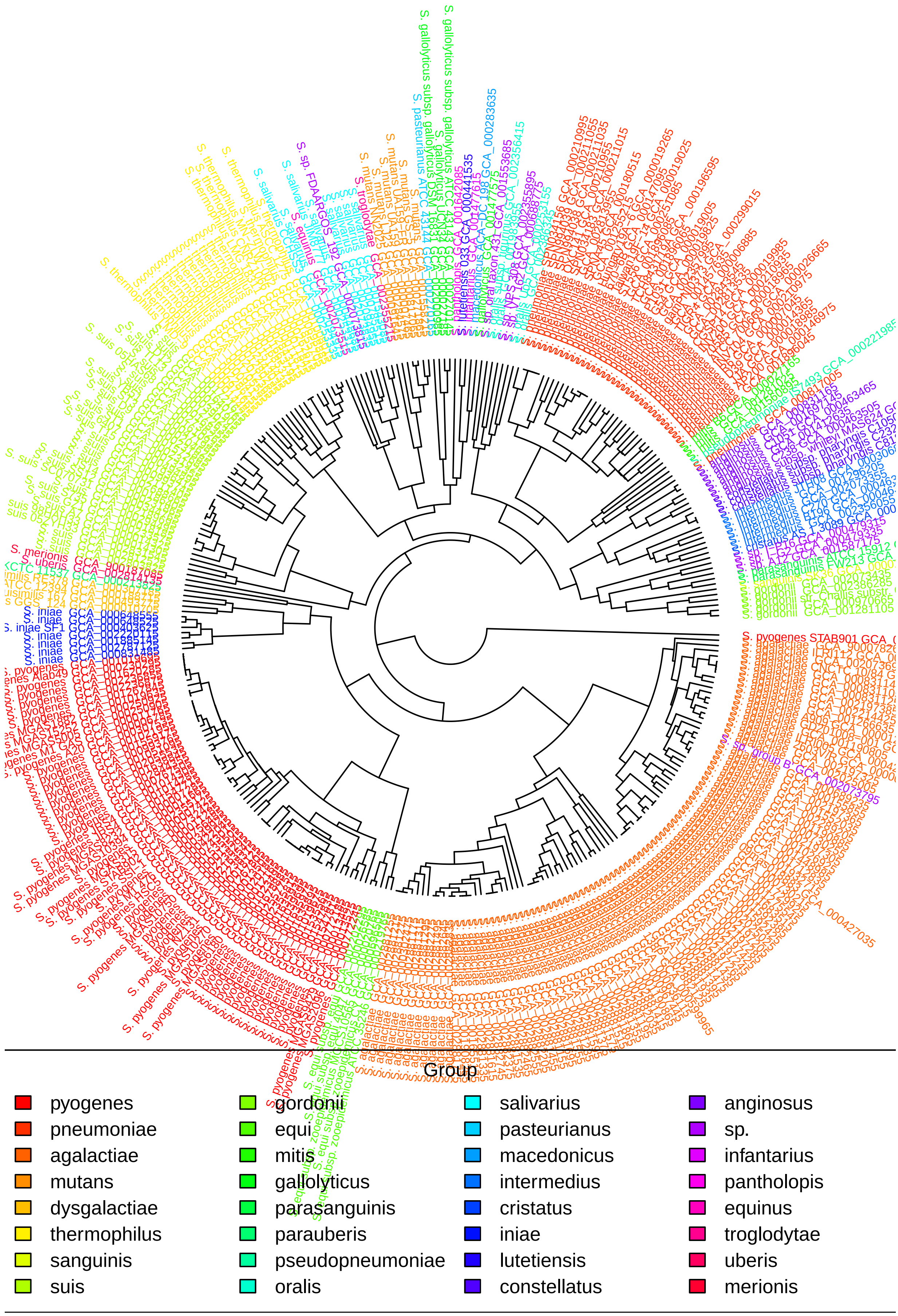

Streptococcus biological_regulation

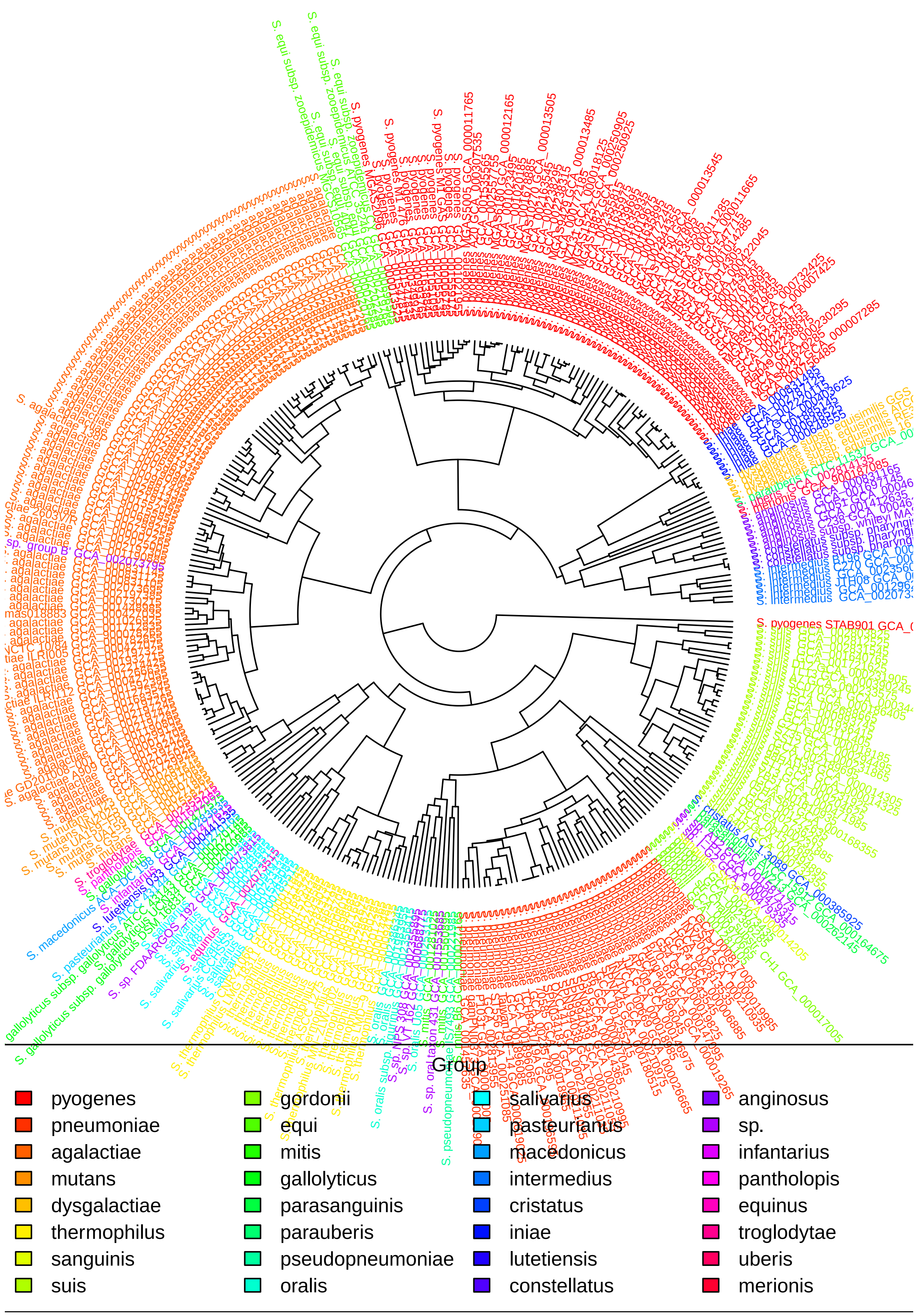

Streptococcus −biological_adhesion

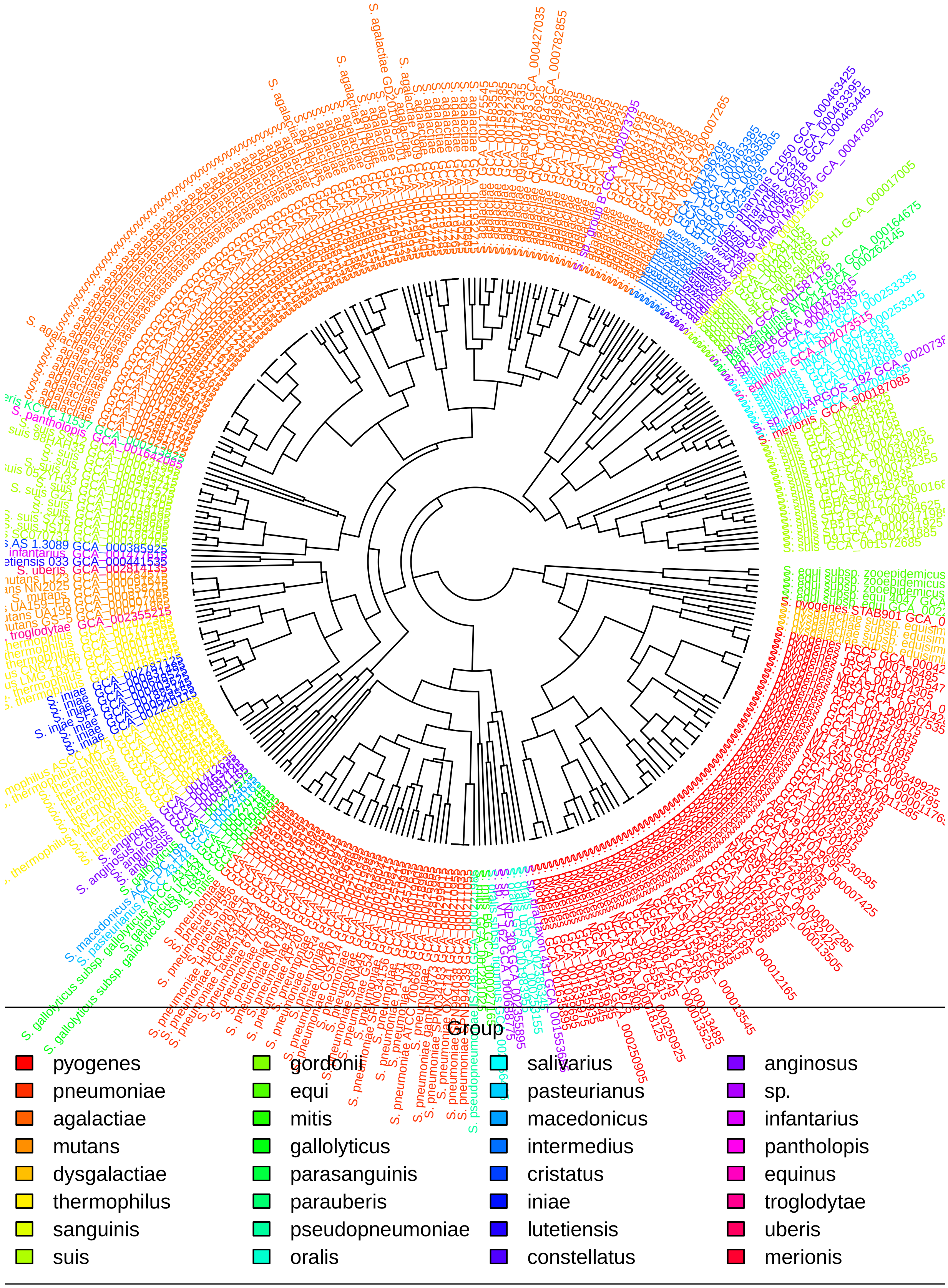

Streptococcus −−adhesion_of_symbiont_to_host

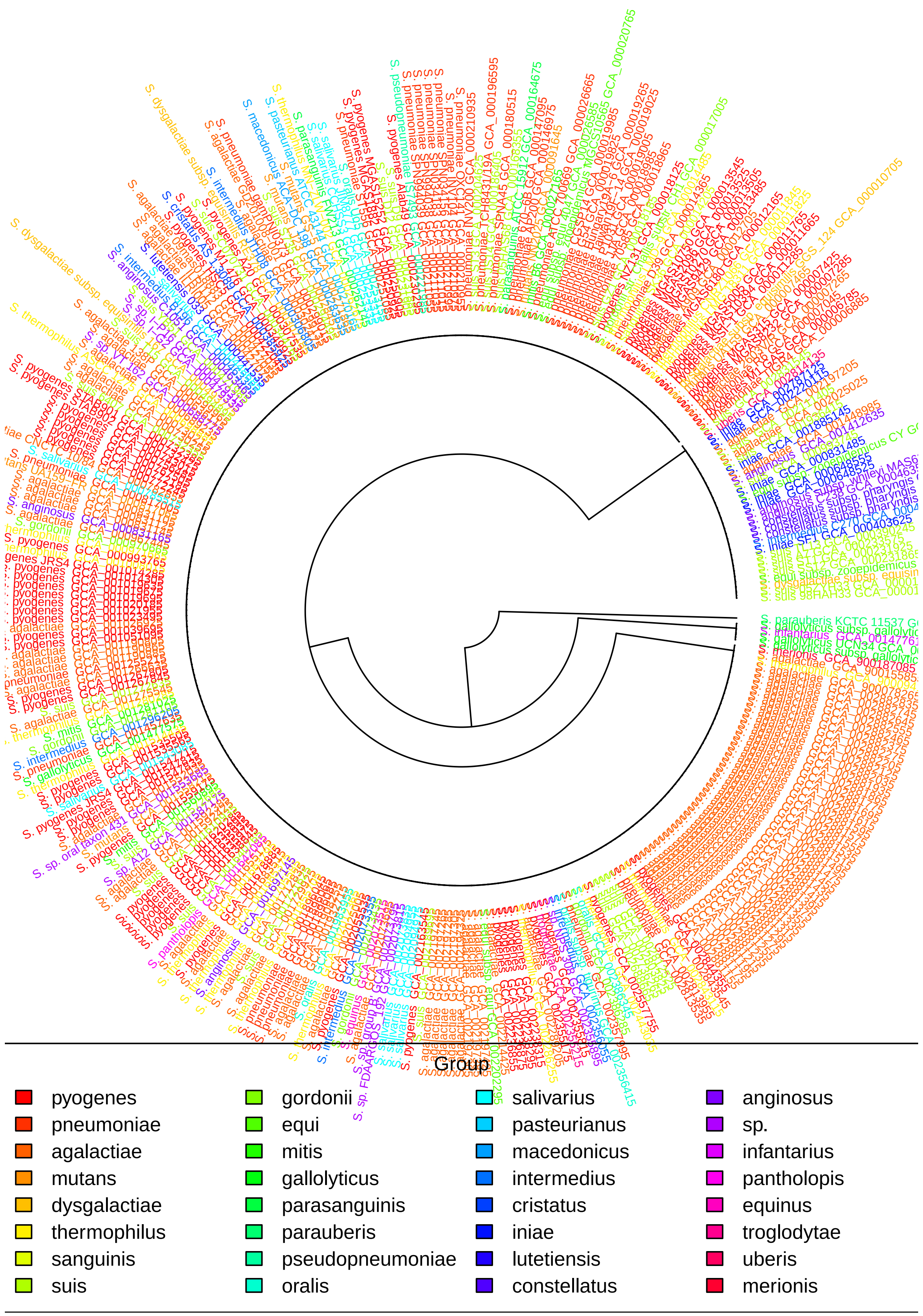

Streptococcus −−multi−organism_process

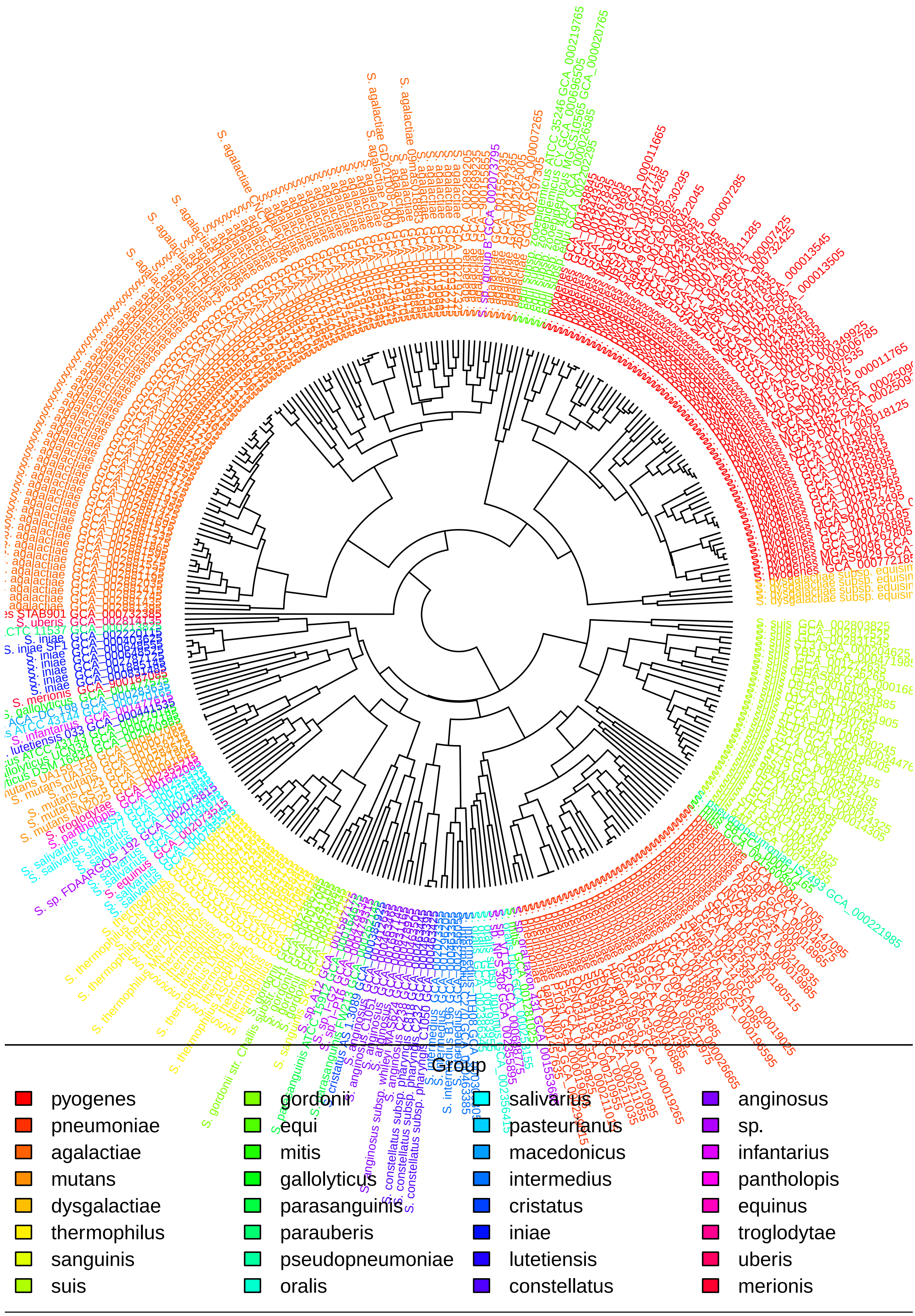

Streptococcus --inter_species_interaction_between_organisms

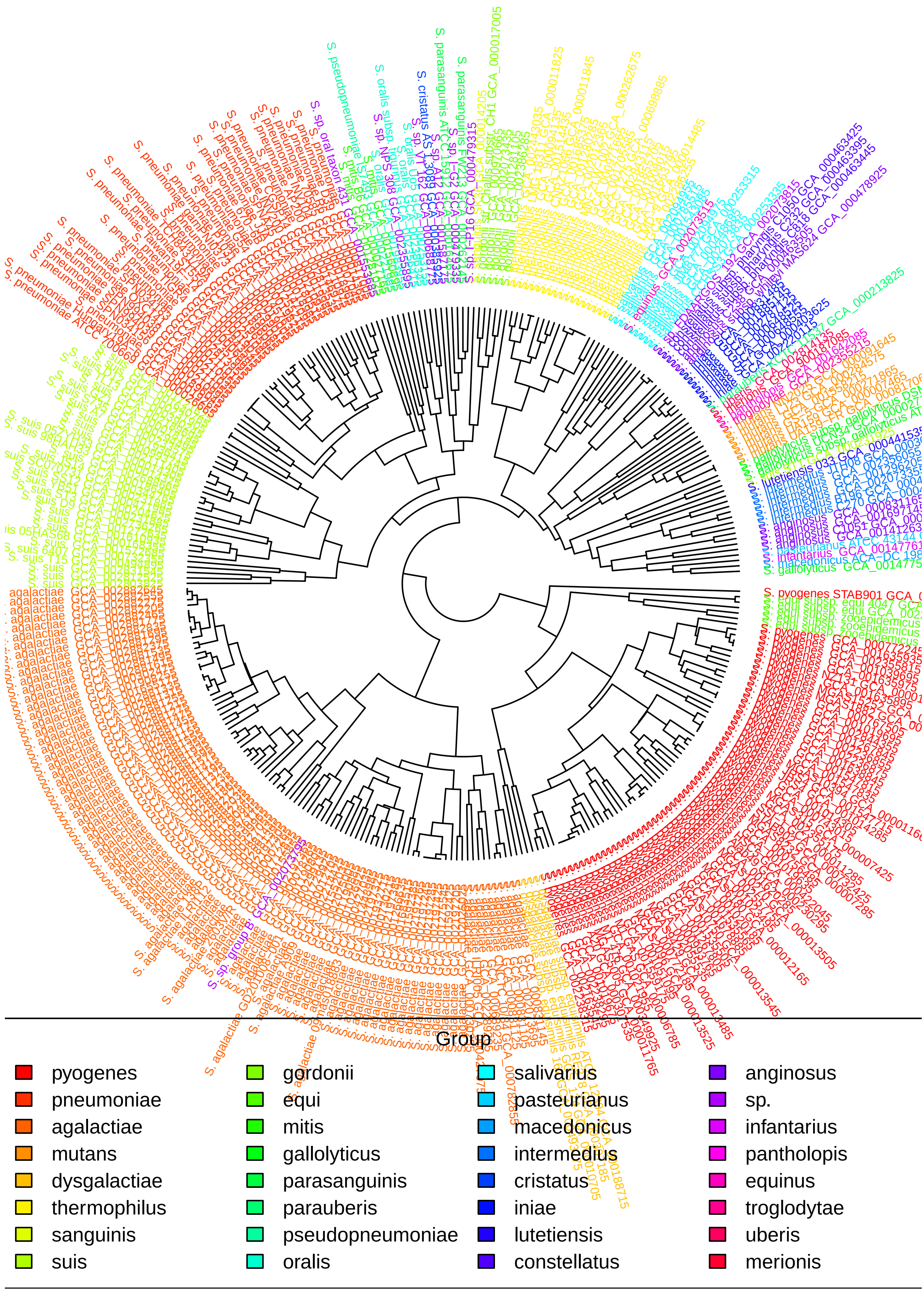

Streptococcus --biofilm_formation

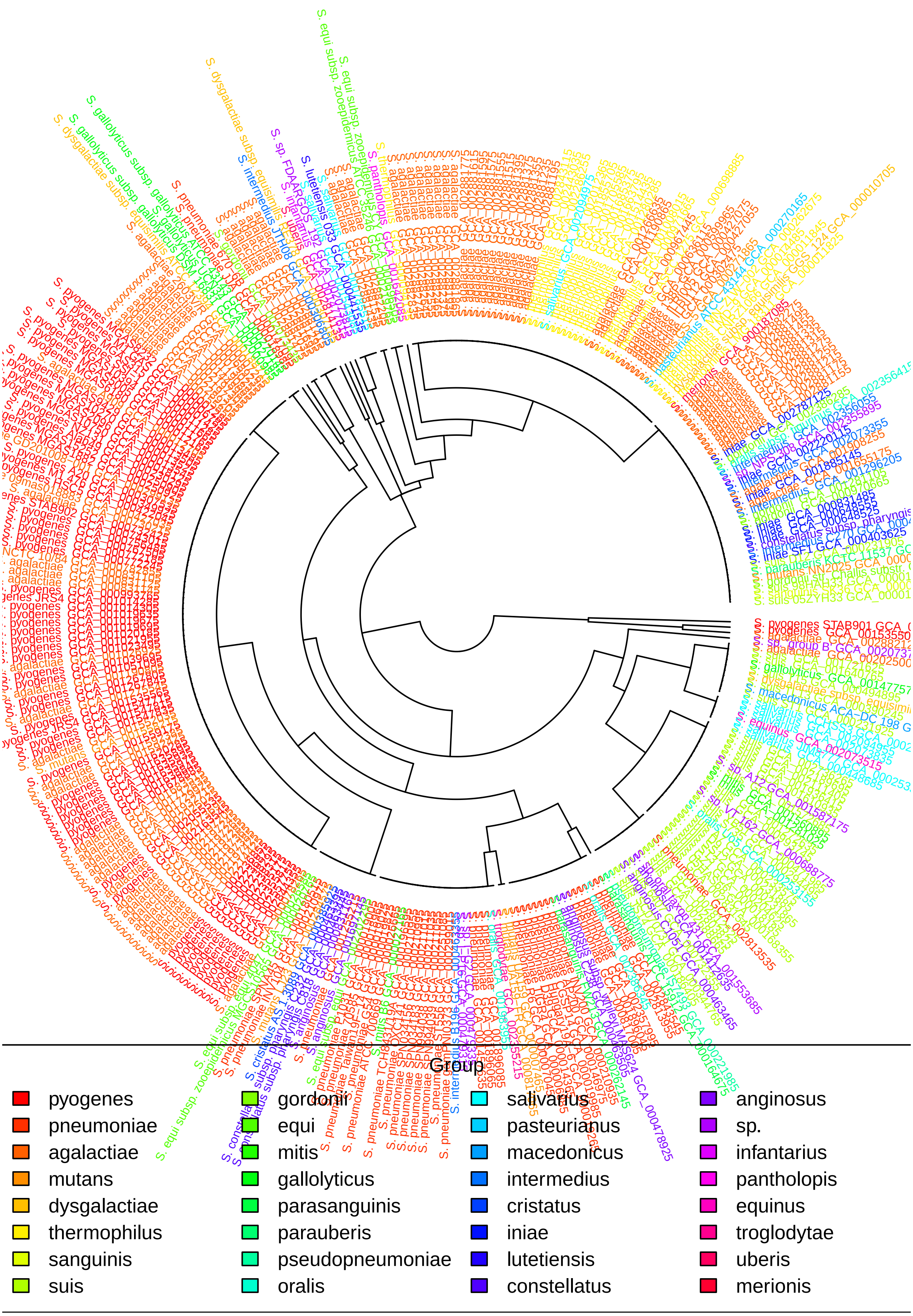

Streptococcus −cell_aggregation

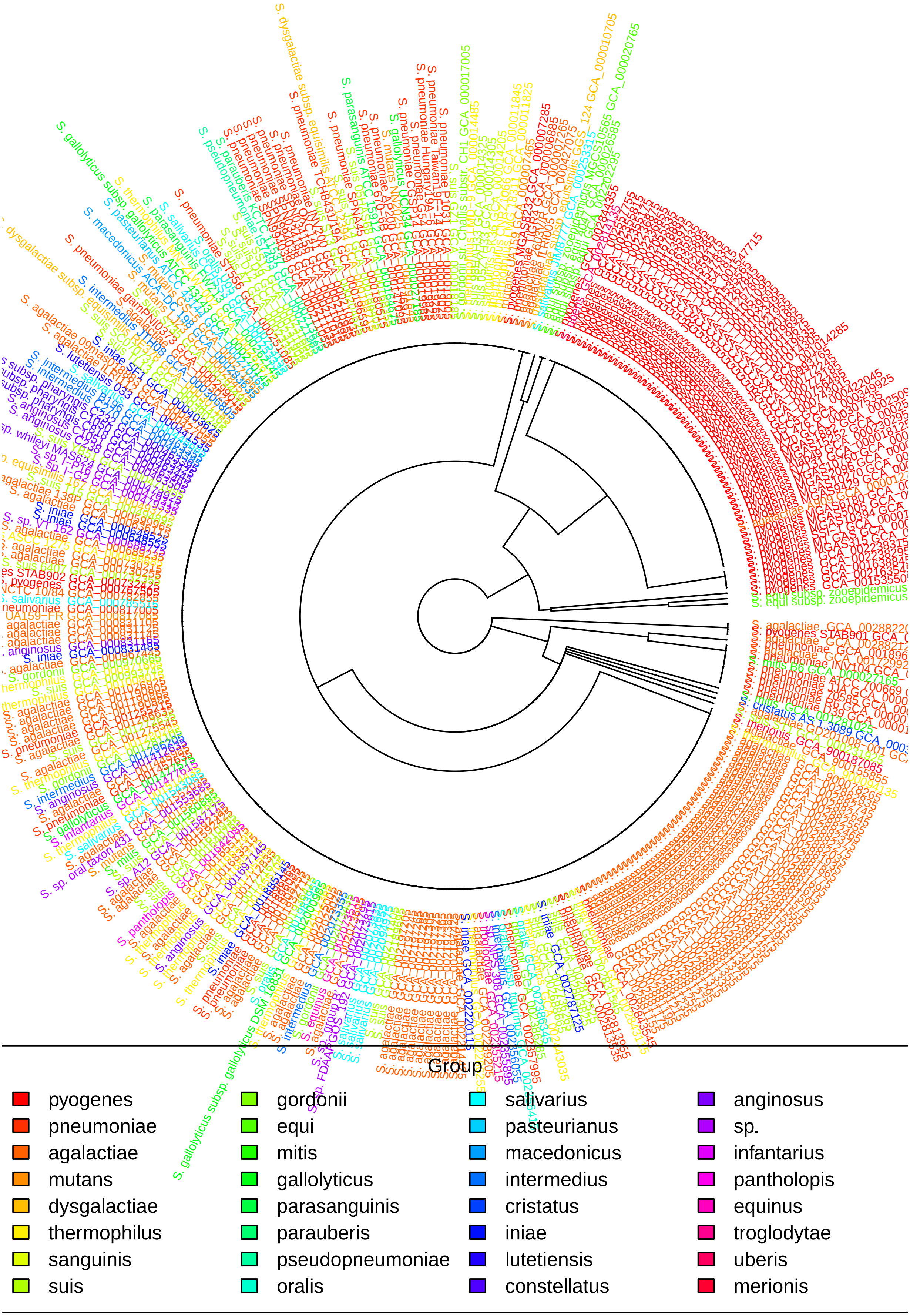

Streptococcus −−symbiont_process

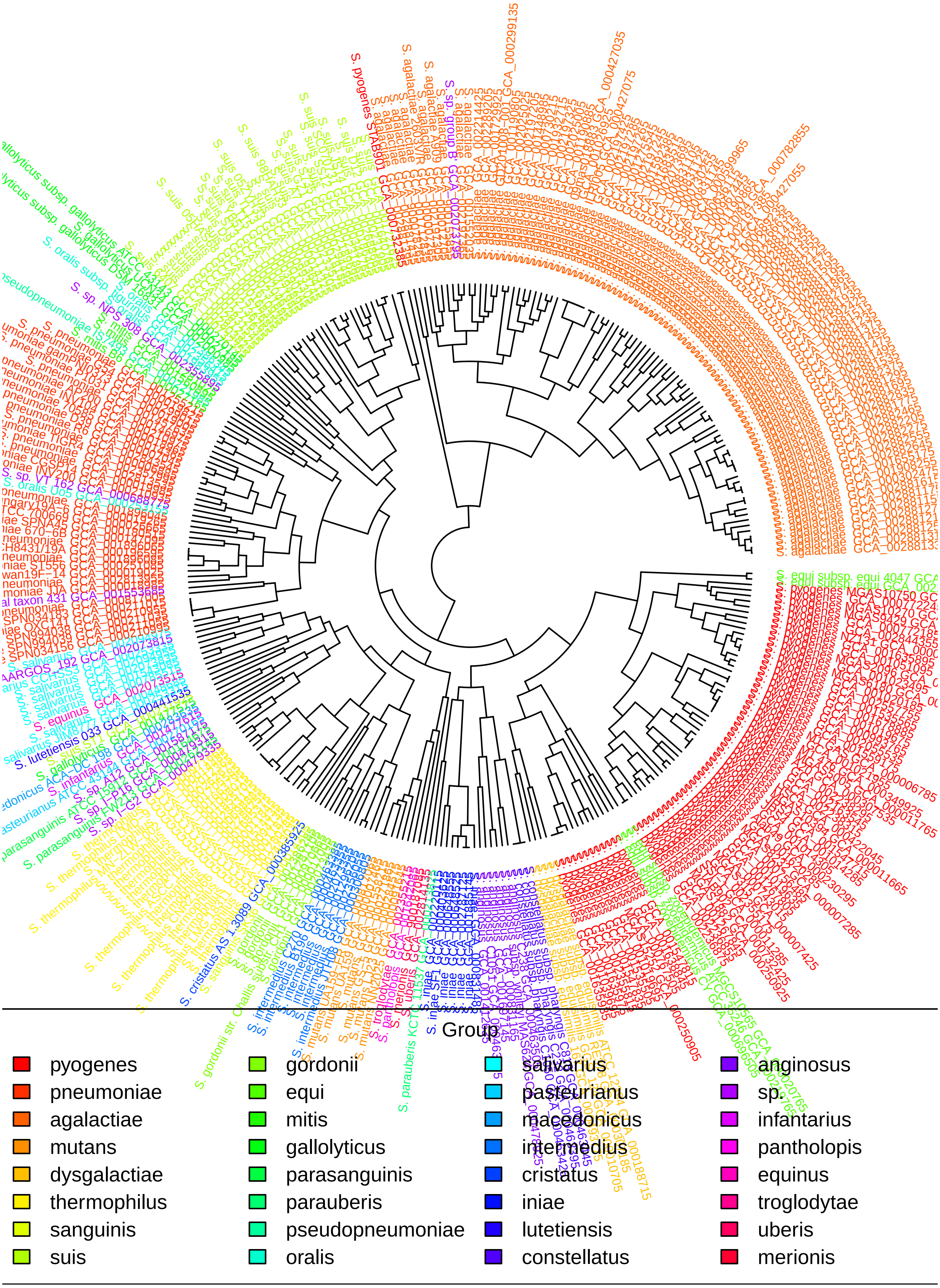

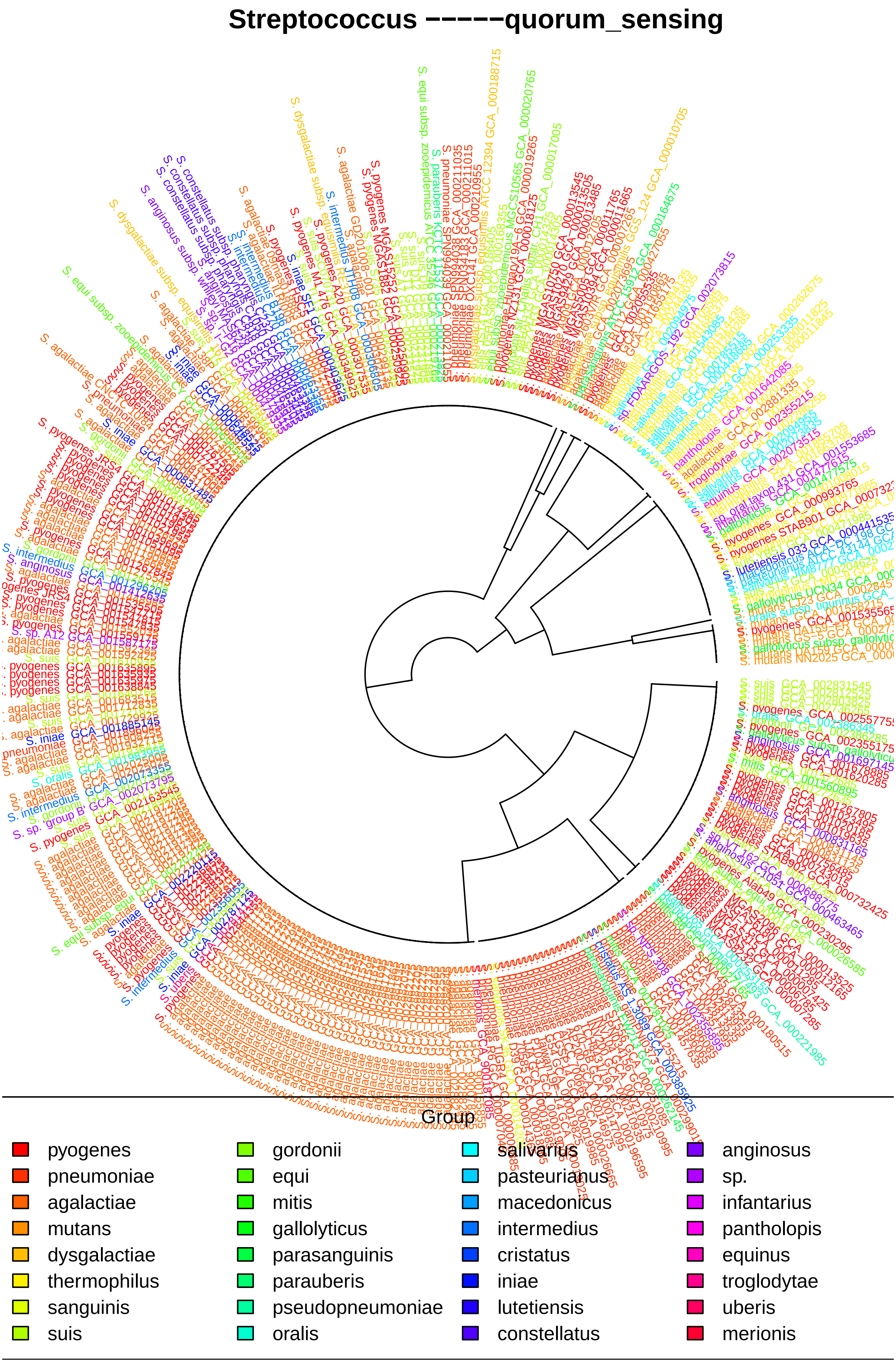
Streptococcus -----quorum_sensing
Group
pyogenes
pneumoniae
agalactiae
mutans
dysgalactiae
thermophilus
sanguinis
suis
gordonii
equi
mitis
gallolyticus
parasanguinis
parauberis
pseudopneumoniae
oralis
salivarius
pasteurianus
macedonicus
intermedius
cristatus
iniae
lutetiensis
constellatus
anginosus
sp.
infantarius
pantholopis
equinus
troglodytae
uberis
merionis

eptococcus ---modification_of _morphology_or_physiology_of_other_or

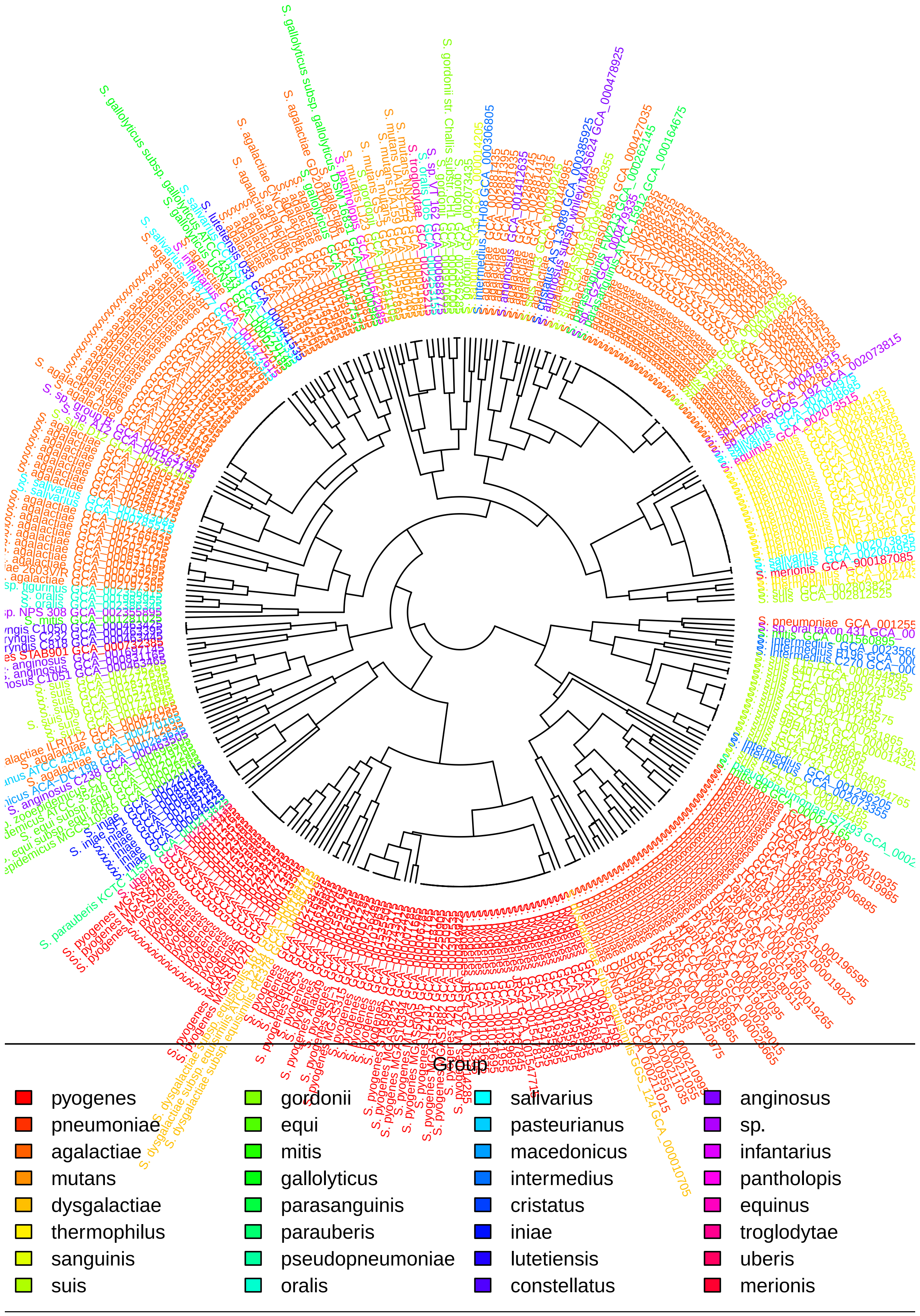

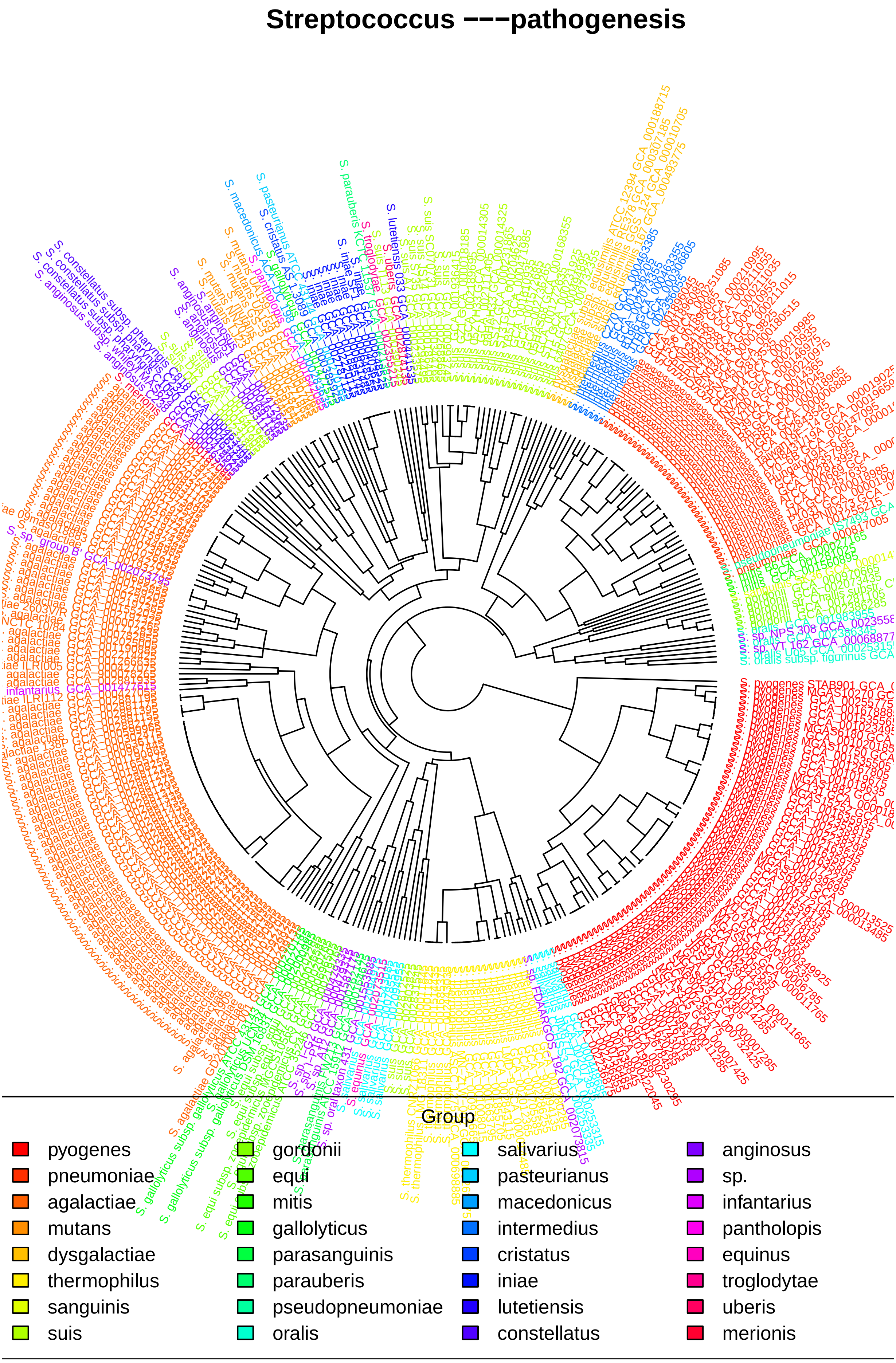
Streptococcus ---pathogenesis
Group
pyogenes
pneumoniae
agalactiae
mutans
dysgalactiae
thermophilus
sanguinis
suis
gordonii
equi
mitis
gallolyticus
parasanguinis
parauberis
pseudopneumoniae
oralis
salivarius
pasteurianus
macedonicus
intermedius
cristatus
iniae
lutetiensis
constellatus
anginosus
sp.
infantarius
pantholopis
equinus
troglodytae
uberis
merionis
