# Supplementary material for: Phenotype and multi-omics comparison of Staphylococcus and Streptococcus uncovers pathogenic traits and predicts zoonotic potential
Source: BMC Genomics. 2021 Feb 4;22:102. doi: 10.1186/s12864-021-07388-6 (PMC7860044; doi:10.1186/s12864-021-07388-6)
Supplement: Supplementary file 13 — Additional file 13. Staphylococcus & Streptococcus combined t-SNE [file 12864_2021_7388_MOESM13_ESM.pdf]

All proteins

All architectures

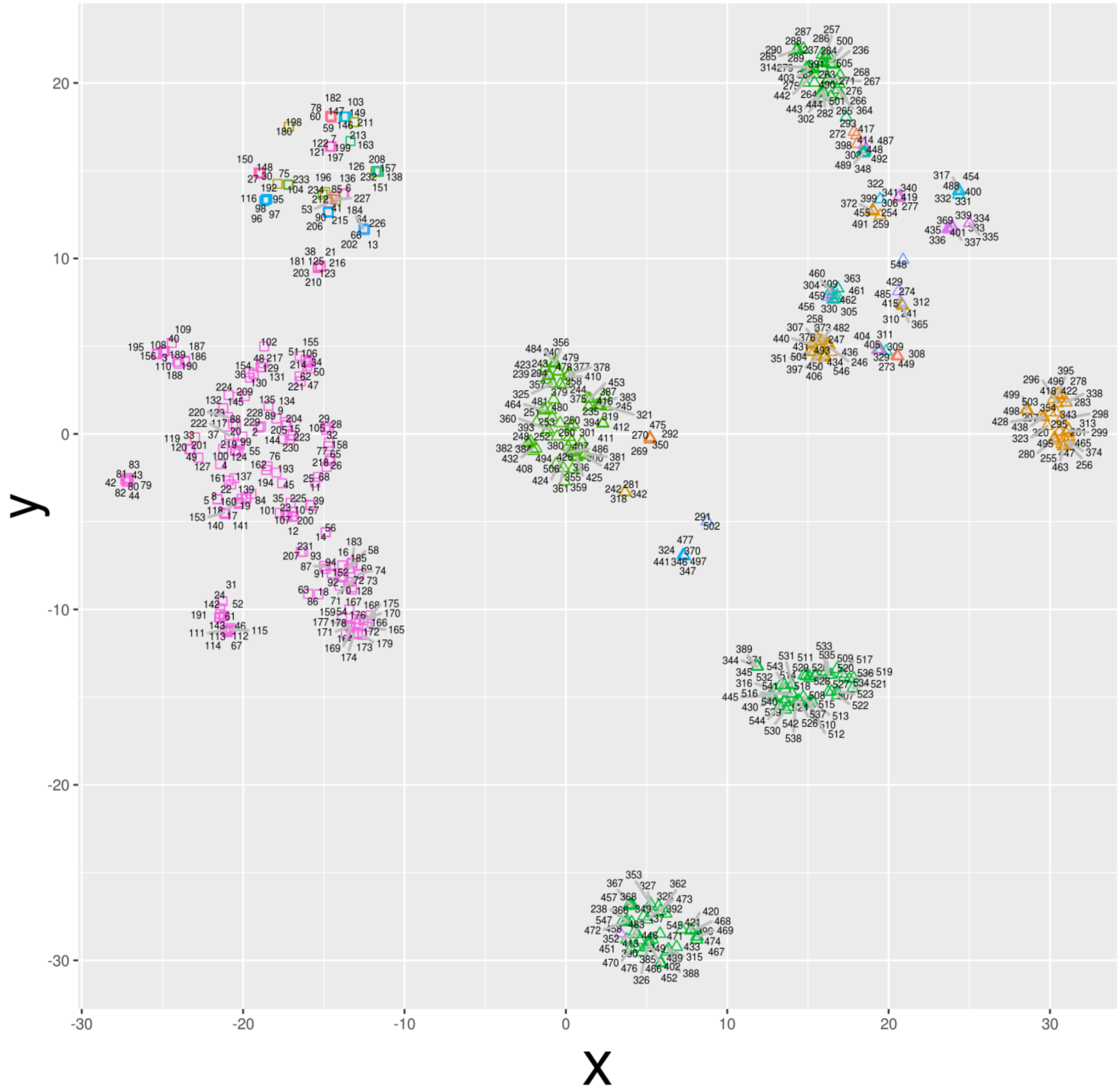

All proteins with GO annotation

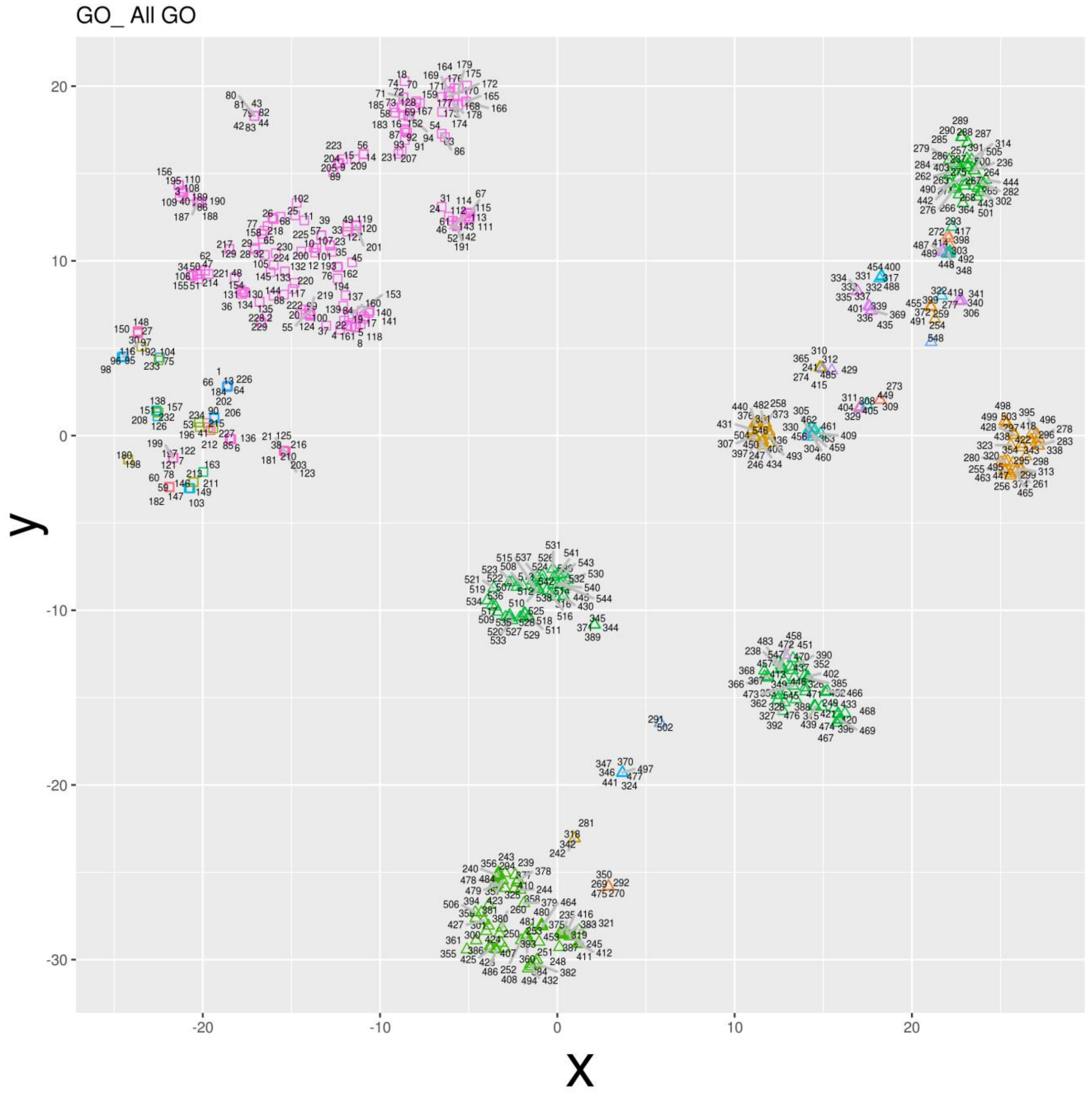

GO:0008150   Biological process

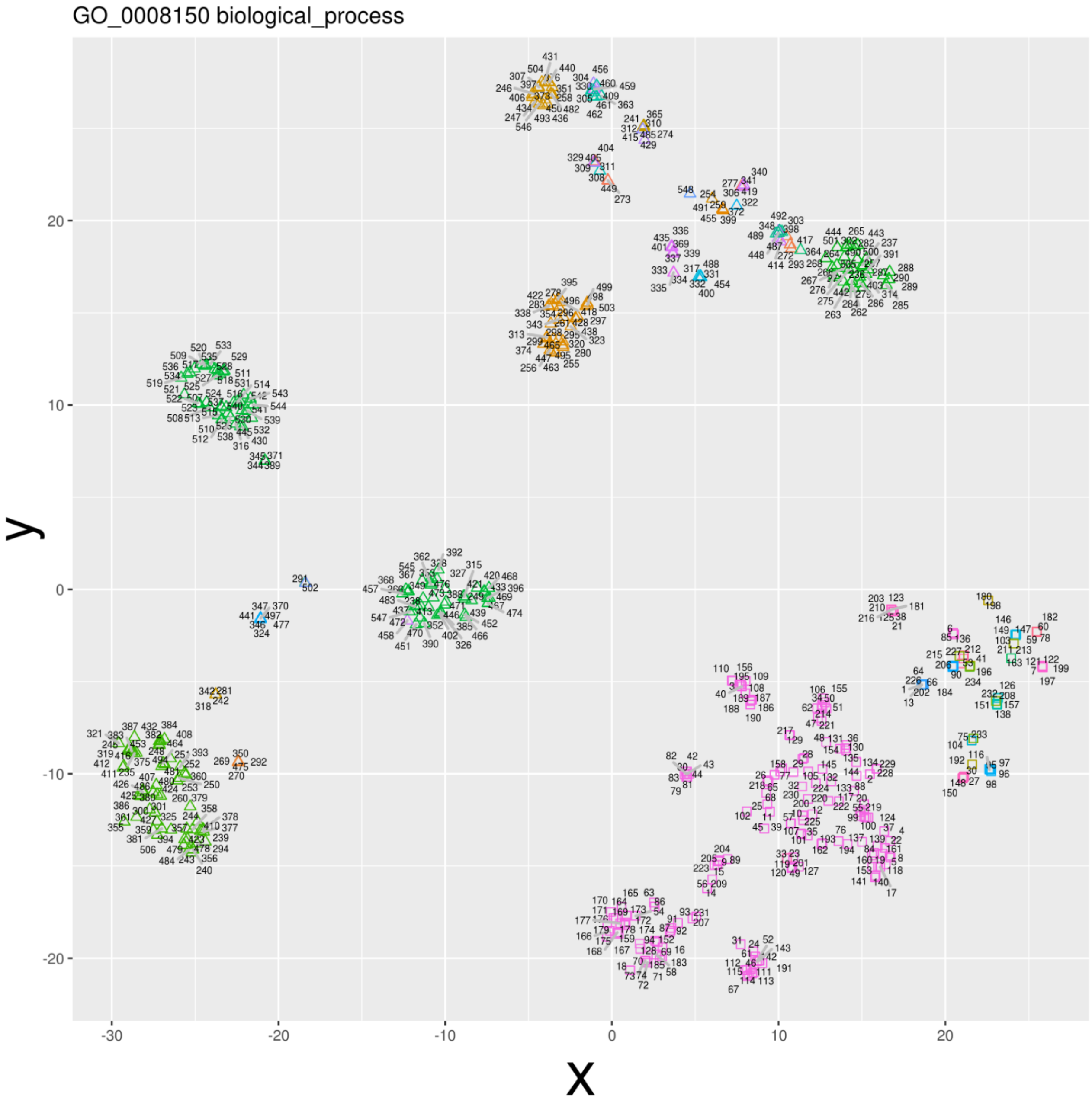

GO:0008152 *Metabolic process

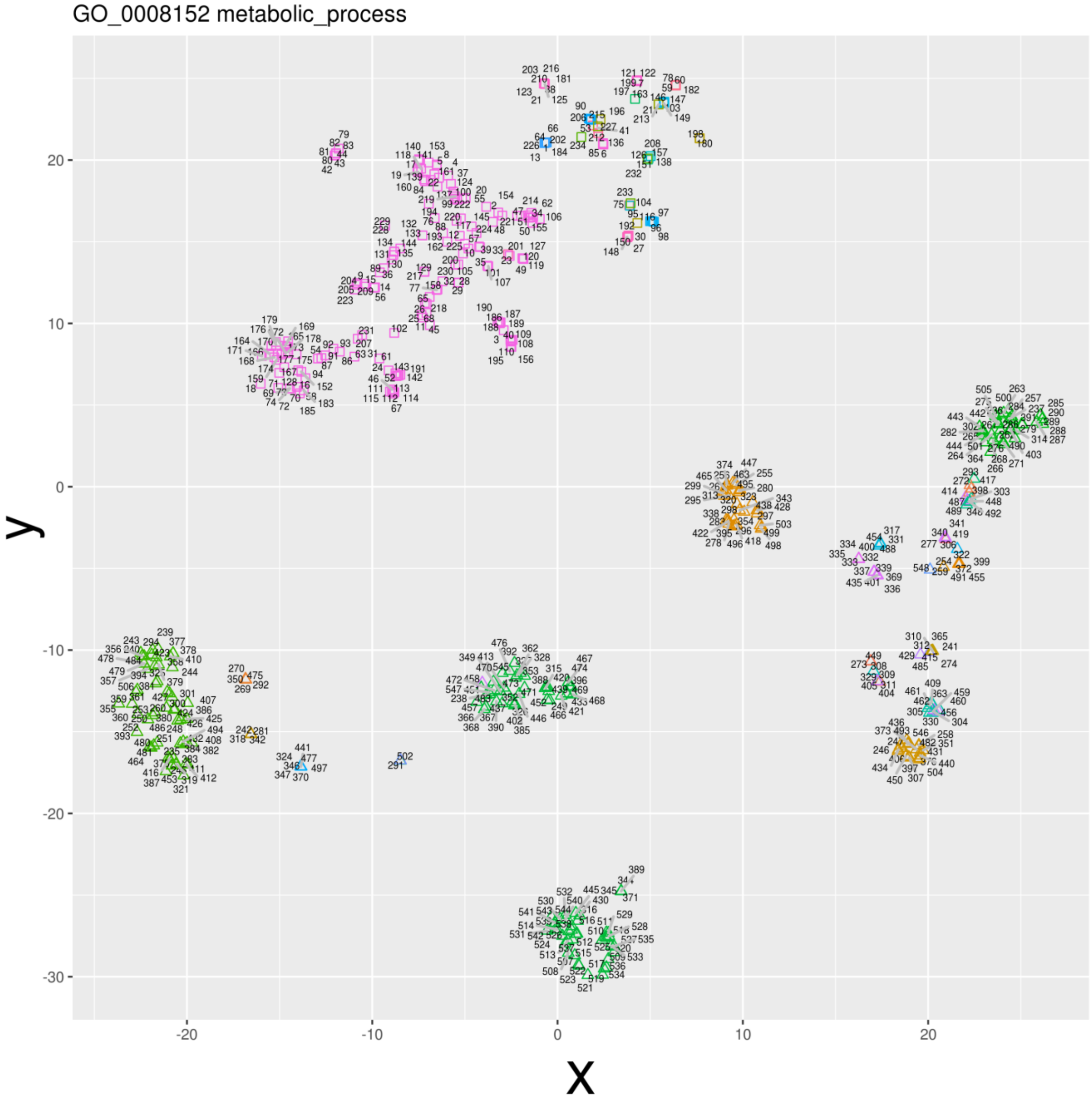

GO:0017144   Drug metabolic process

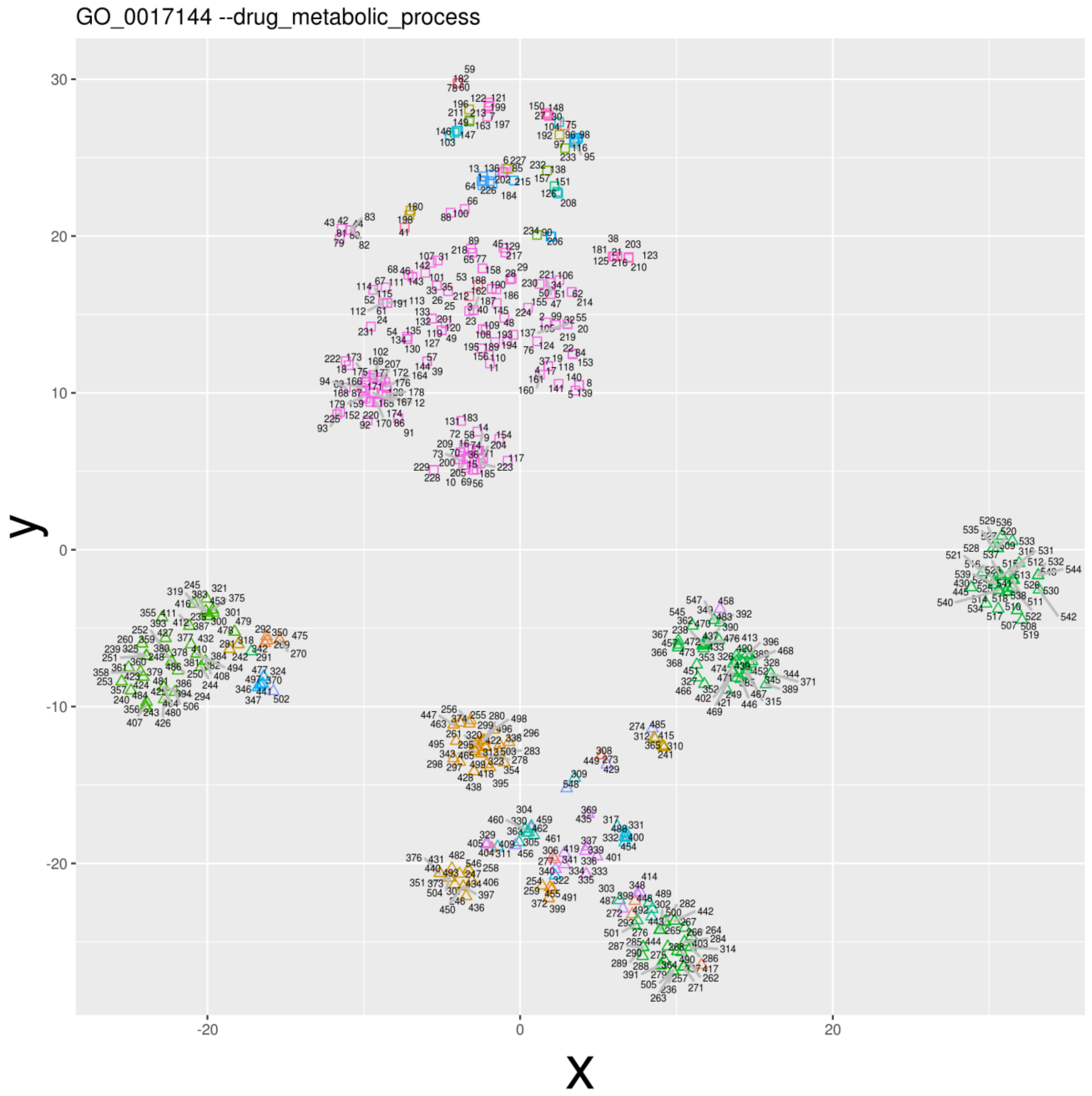

GO:0042493 Response to drug

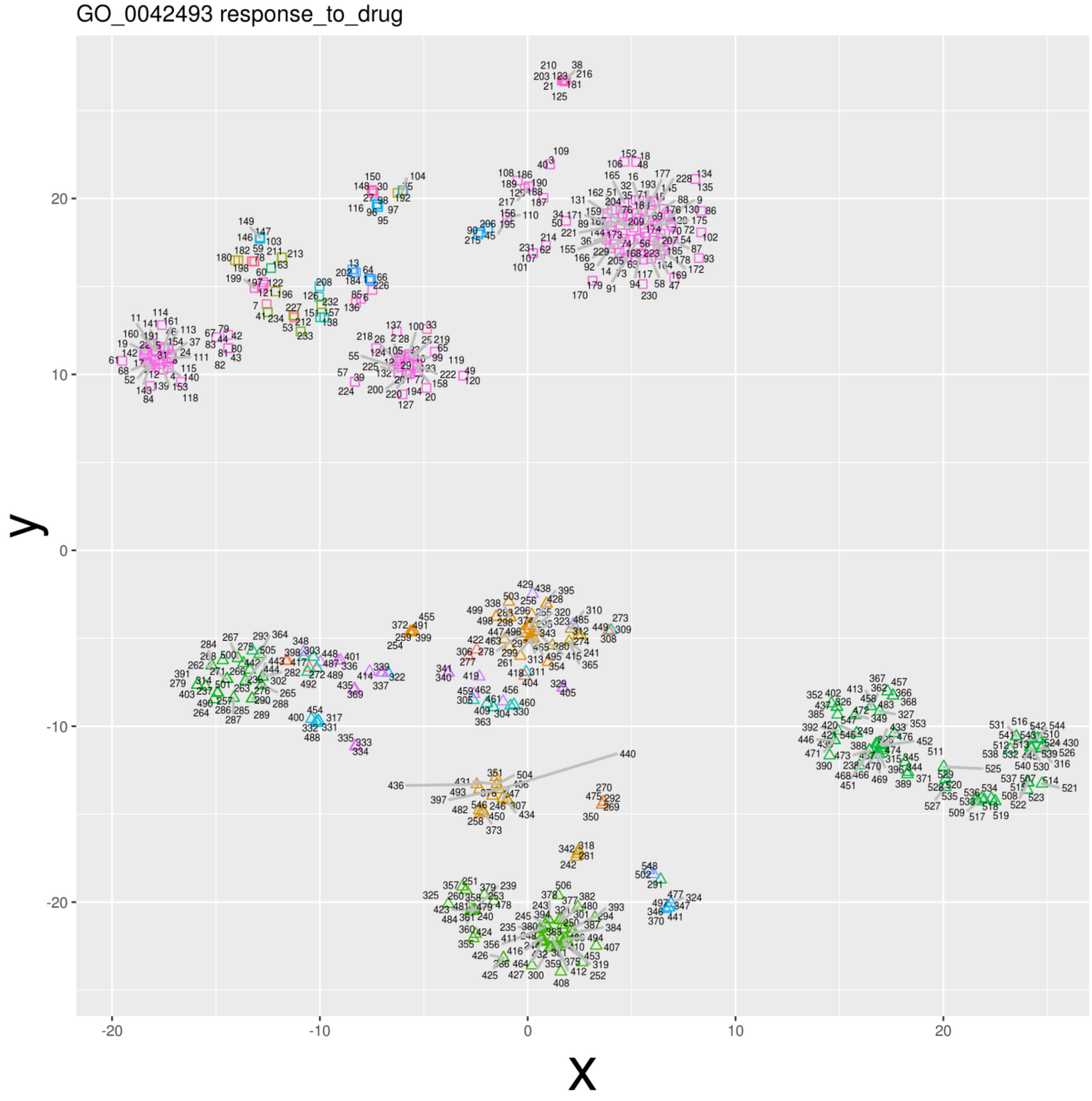

GO:0023052 *Signalling

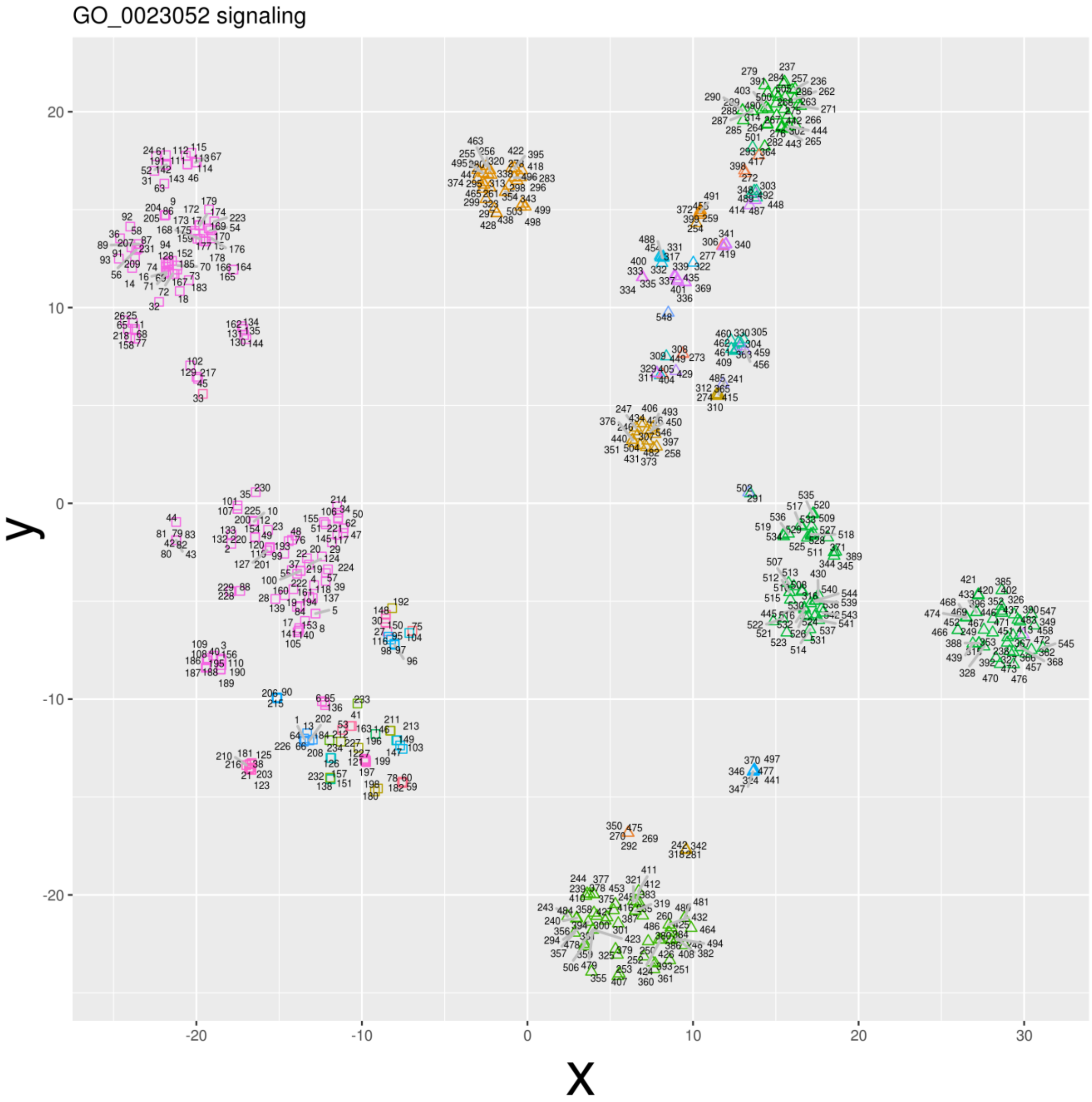

GO:0065007 *Biological regulation

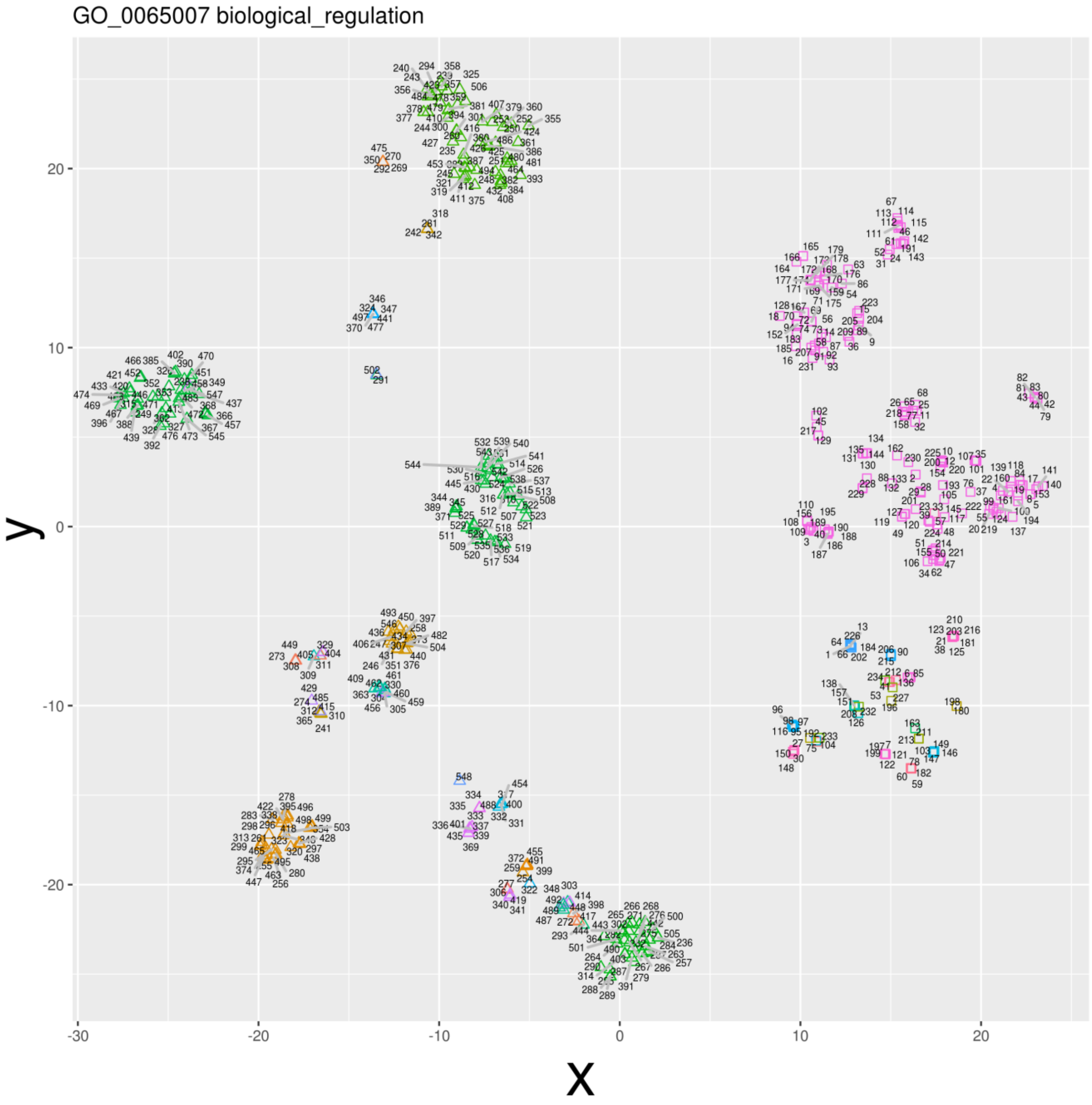

GO:0022610 *Biological adhesion

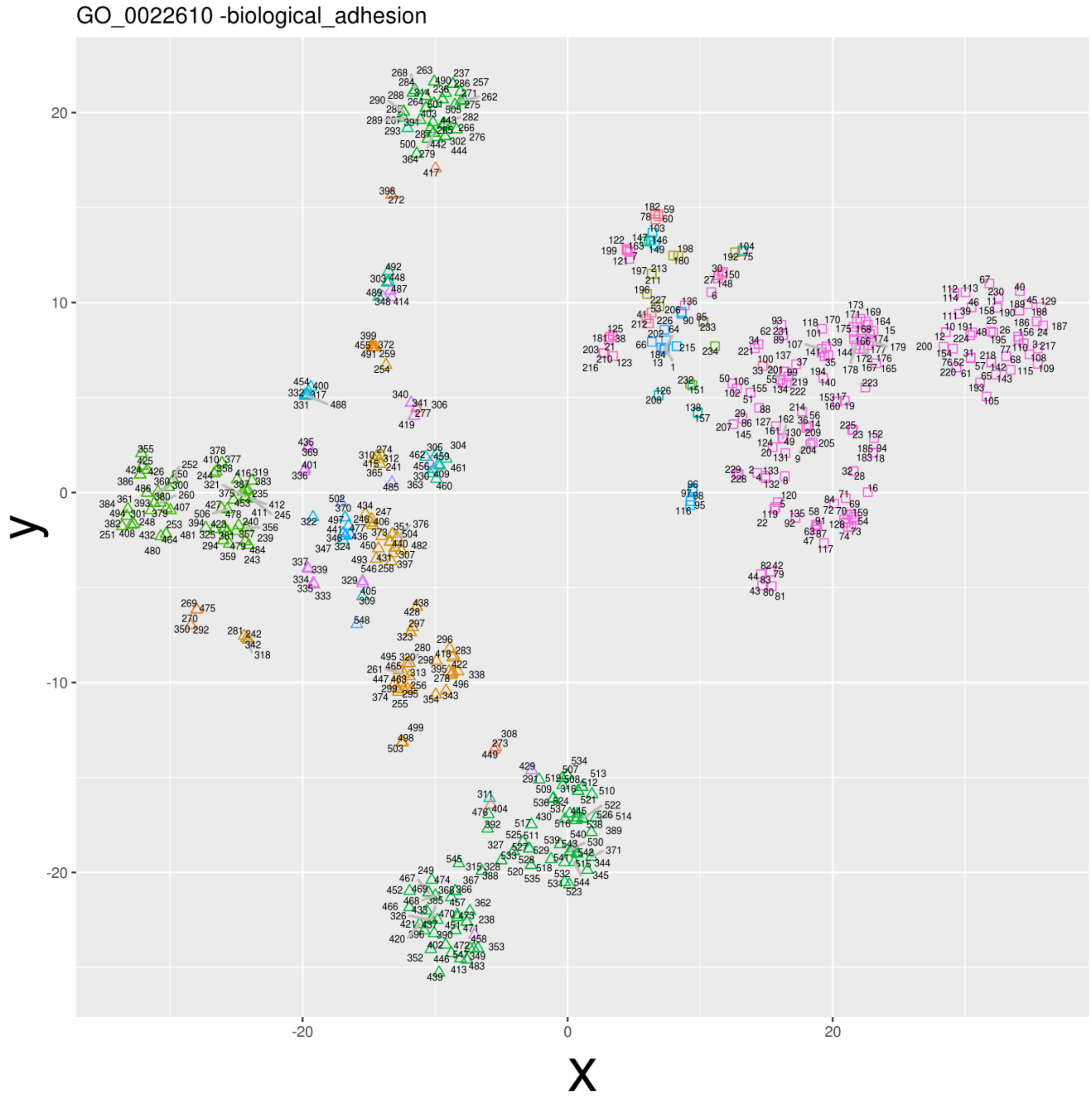

GO:0044419 Inter species interaction between organisms

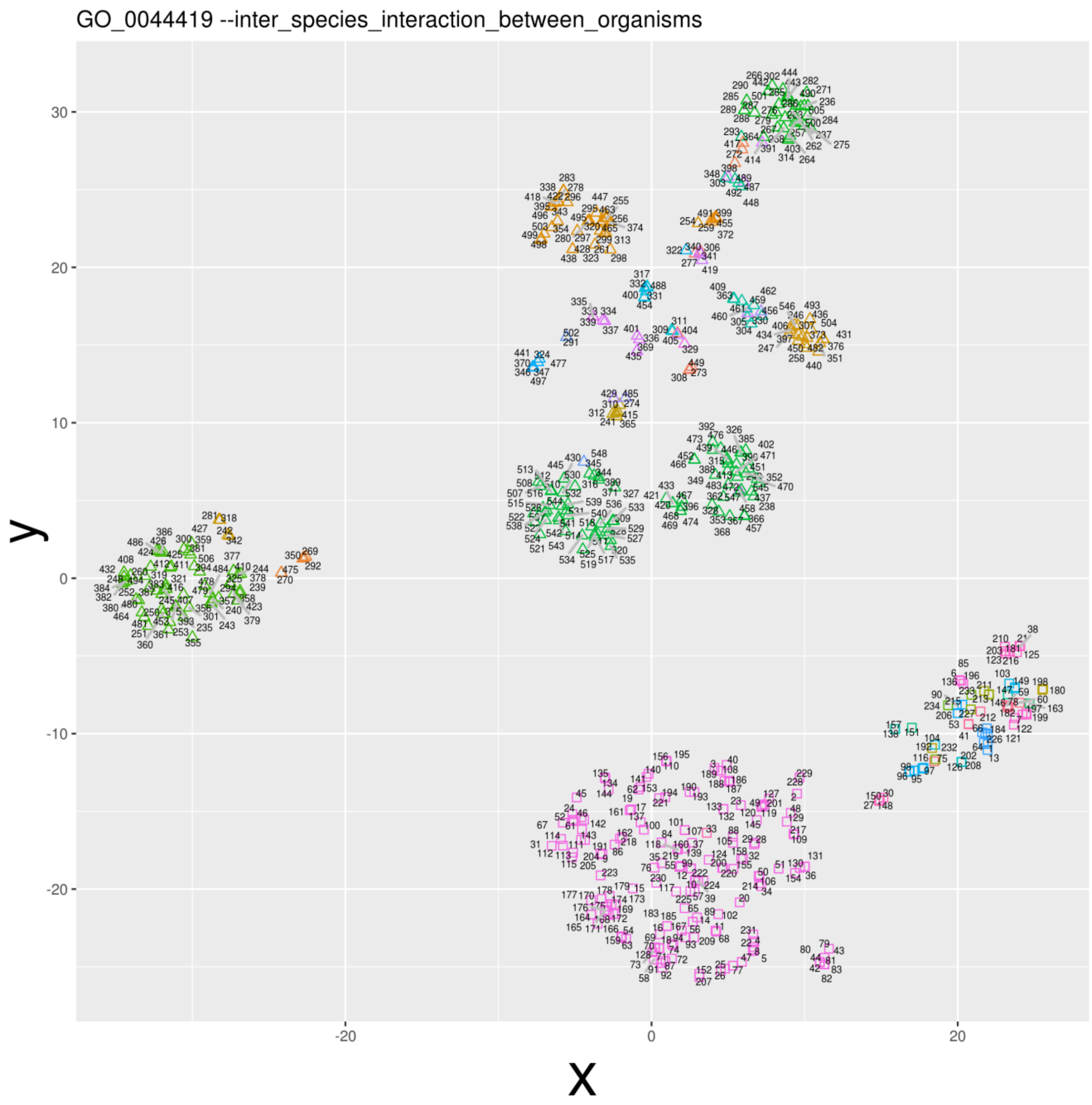

GO:0042710   Biofilm formation

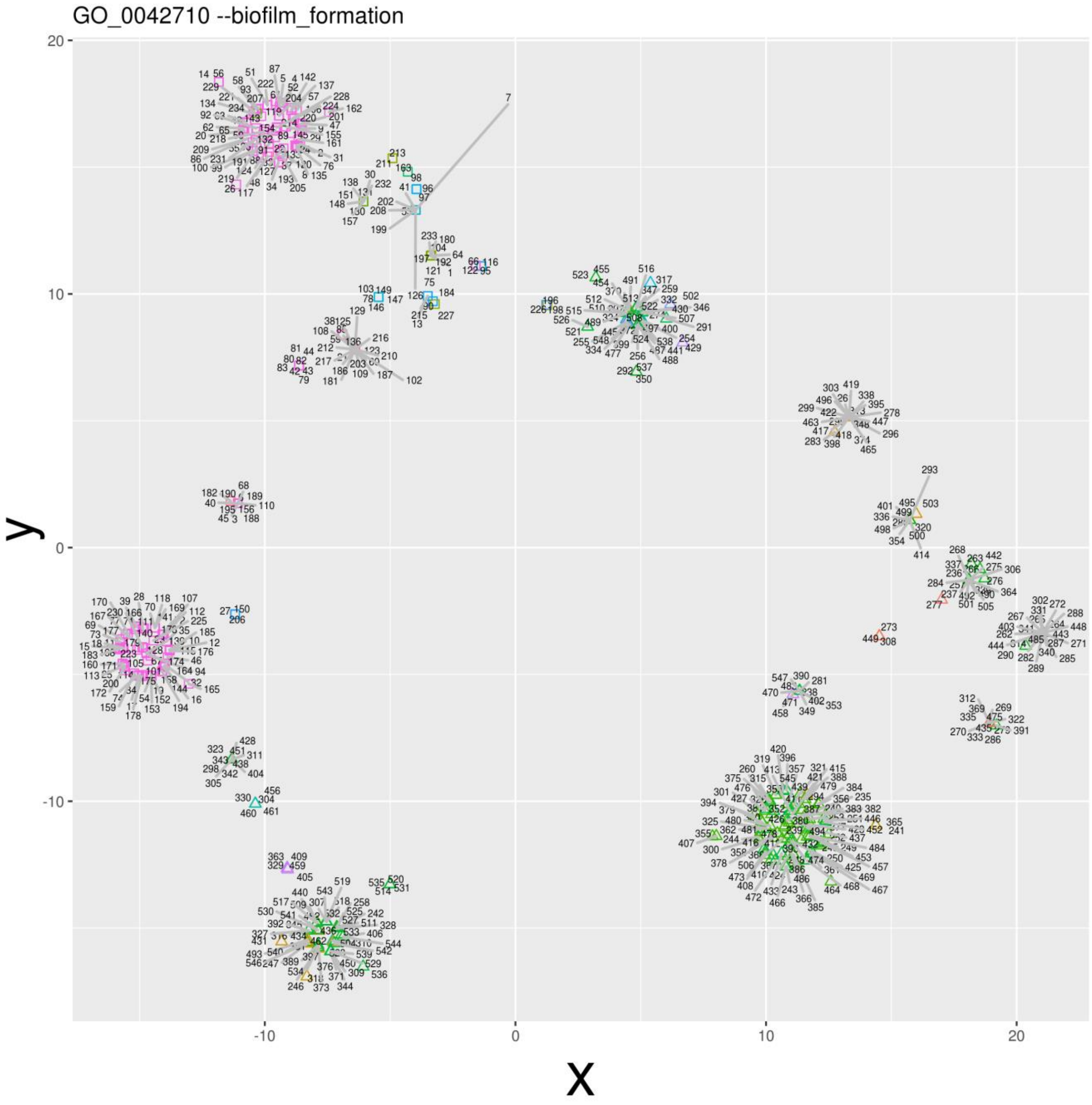

GO:0098743   Cell aggregation

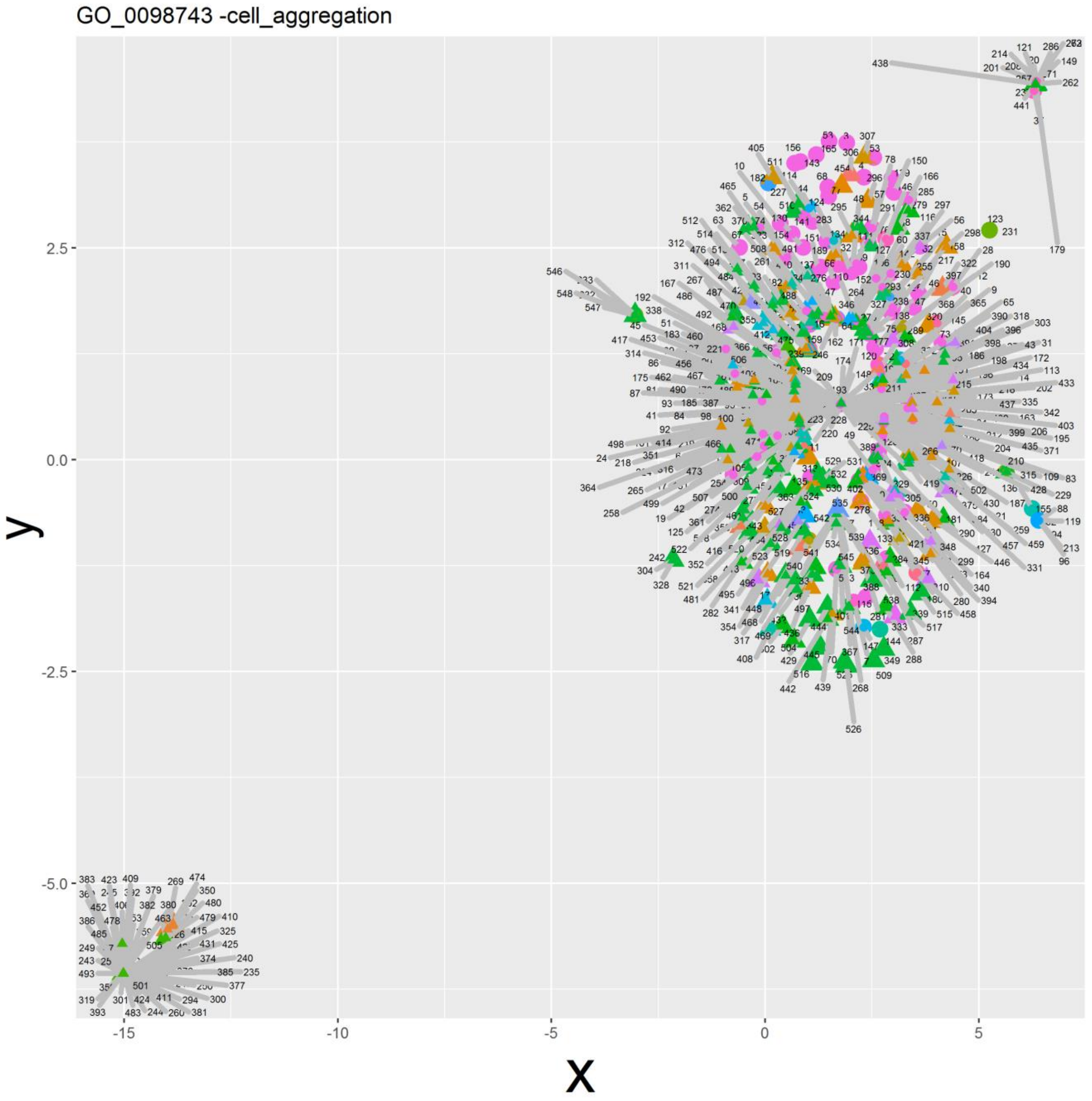

GO:0044403 Symbiont process

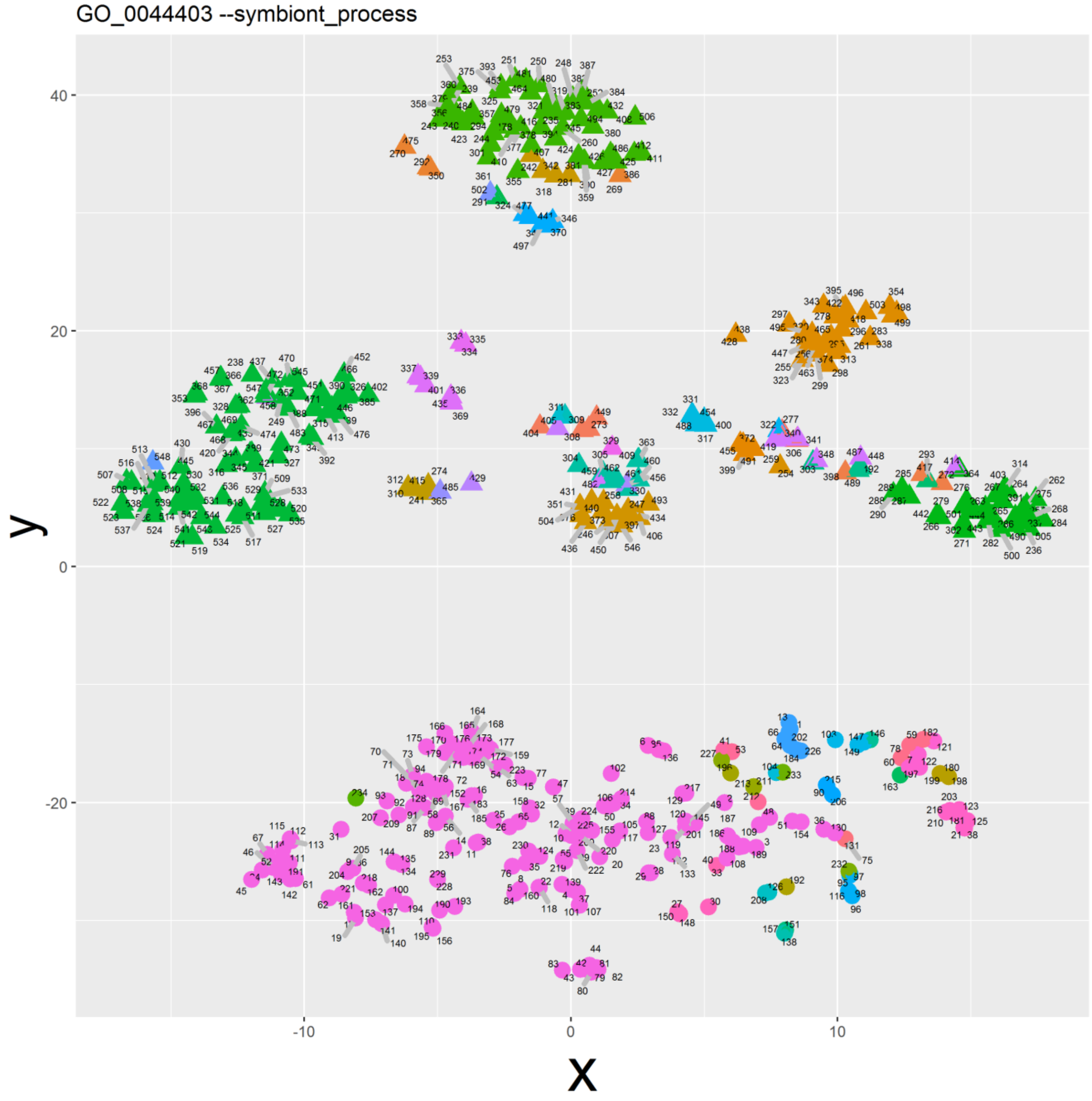

GO:0009372 Quorum sensing

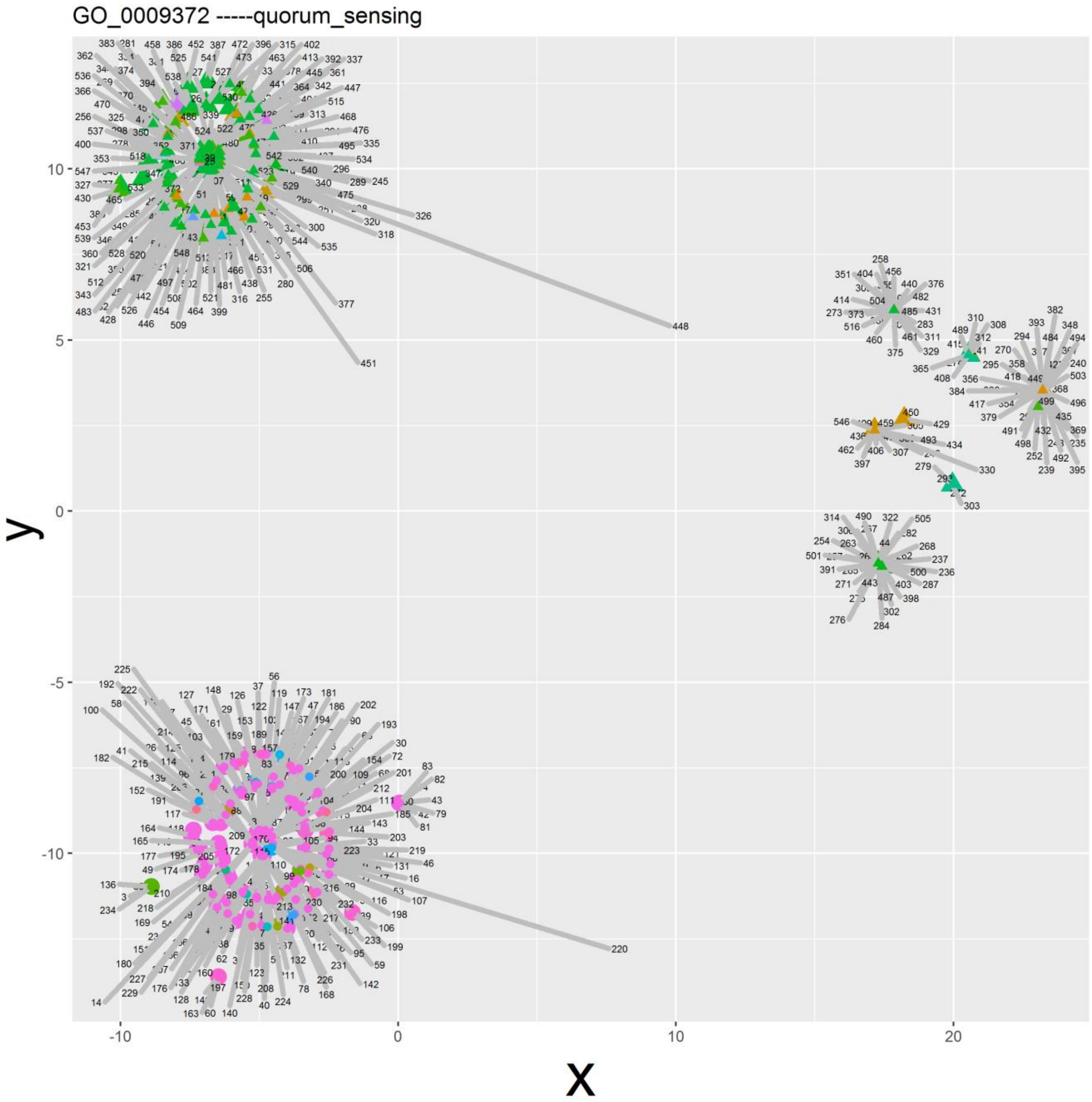

GO:0035821 Modification of morphology or physiology of other organism

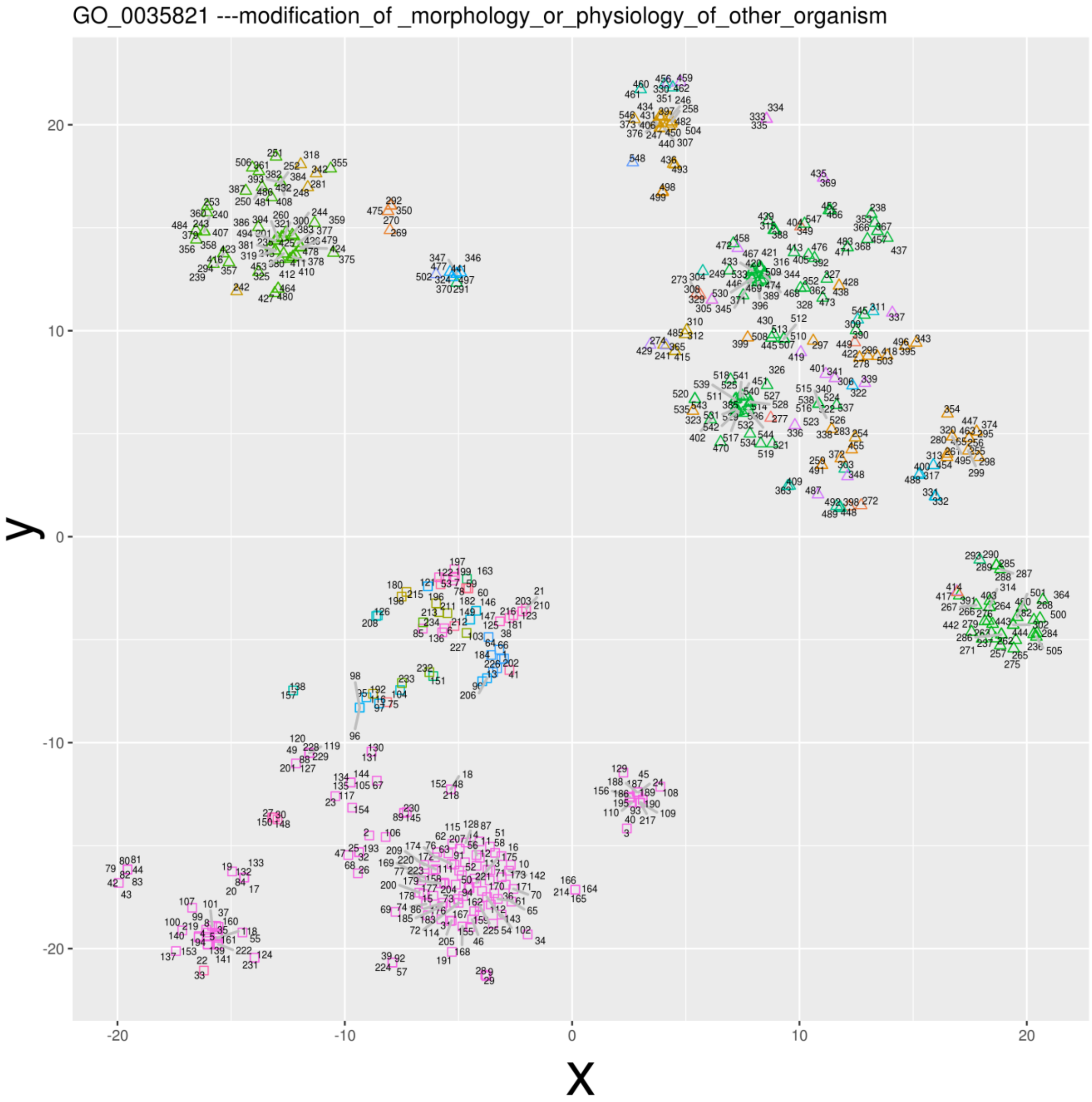

GO:0009405 Pathogenesis

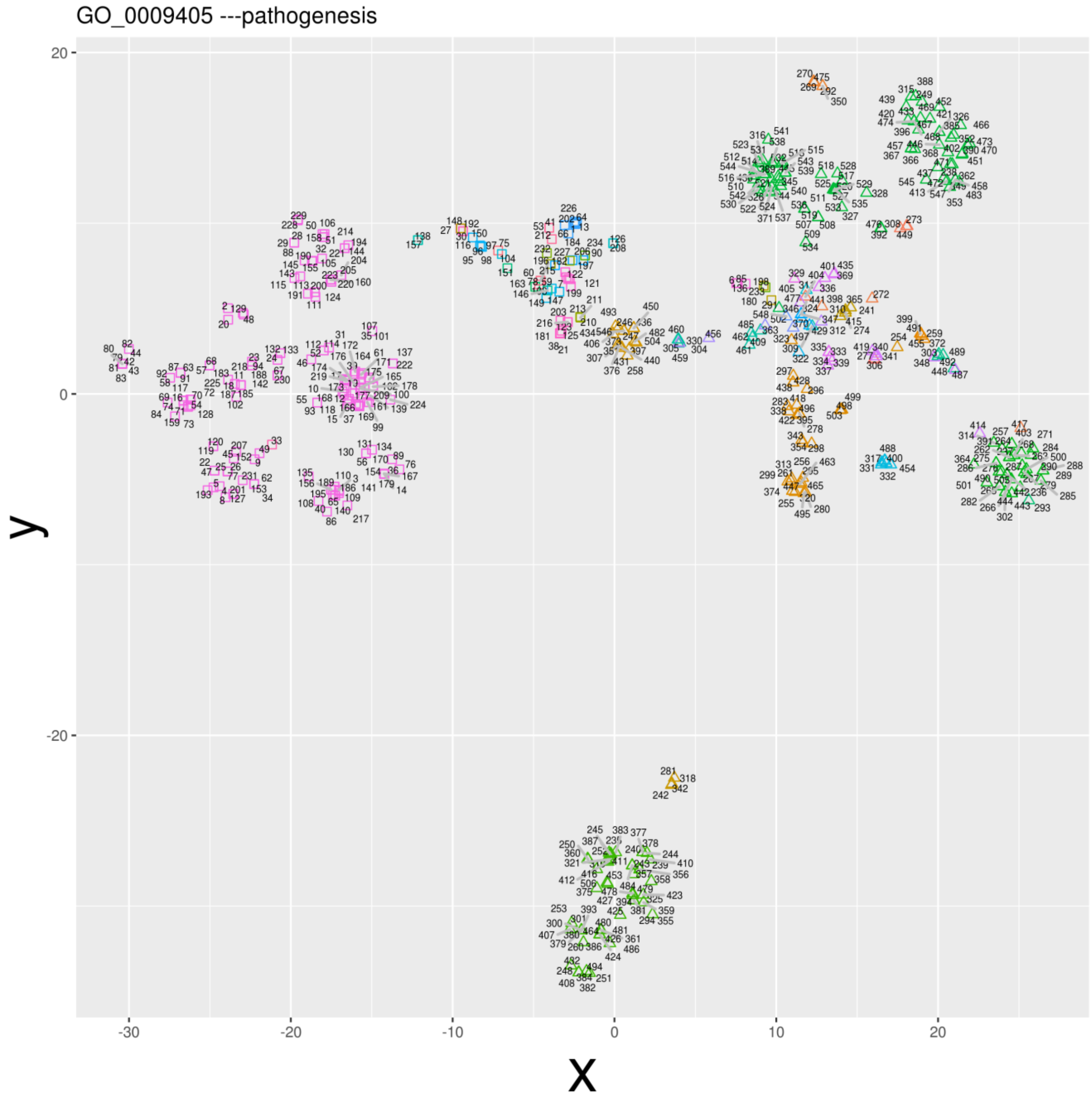

label	group
1	Staph. epidermidis
2	Staph. aureus
3	Staph. aureus
4	Staph. aureus
5	Staph. aureus
6	Staph. haemolyticus
7	Staph. saprophyticus
8	Staph. aureus
9	Staph. aureus
10	Staph. aureus
11	Staph. aureus
12	Staph. aureus
13	Staph. epidermidis
14	Staph. aureus
15	Staph. aureus
16	Staph. aureus
17	Staph. aureus
18	Staph. aureus
19	Staph. aureus
20	Staph. aureus
21	Staph. lugdunensis
22	Staph. aureus
23	Staph. aureus
24	Staph. aureus
25	Staph. aureus
26	Staph. aureus
27	Staph. pseudintermedius
28	Staph. aureus
29	Staph. aureus
30	Staph. pseudintermedius
31	Staph. aureus
32	Staph. aureus

33	Staph. argenteus
34	Staph. aureus
35	Staph. aureus
36	Staph. aureus
37	Staph. aureus
38	Staph. lugdunensis
39	Staph. aureus
40	Staph. aureus
41	Staph. warneri
42	Staph. aureus
43	Staph. aureus
44	Staph. aureus
45	Staph. aureus
46	Staph. aureus
47	Staph. aureus
48	Staph. aureus
49	Staph. aureus
50	Staph. aureus
51	Staph. aureus
52	Staph. aureus
53	Staph. pasteuri
54	Staph. aureus
55	Staph. aureus
56	Staph. aureus
57	Staph. aureus
58	Staph. aureus
59	Staph. xylosus
60	Staph. xylosus
61	Staph. aureus
62	Staph. aureus
63	Staph. aureus
64	Staph. epidermidis
65	Staph. aureus
66	Staph. epidermidis

67	Staph. aureus
68	Staph. aureus
69	Staph. aureus
70	Staph. aureus
71	Staph. aureus
72	Staph. aureus
73	Staph. aureus
74	Staph. aureus
75	Staph. hyicus
76	Staph. aureus
77	Staph. aureus
78	Staph. xylosus
79	Staph. aureus
80	Staph. aureus
81	Staph. aureus
82	Staph. aureus
83	Staph. aureus
84	Staph. aureus
85	Staph. haemolyticus
86	Staph. aureus
87	Staph. aureus
88	Staph. aureus
89	Staph. aureus
90	Staph. capitis
91	Staph. aureus
92	Staph. aureus
93	Staph. aureus
94	Staph. aureus
95	Staph. schleiferi
96	Staph. schleiferi
97	Staph. schleiferi
98	Staph. schleiferi
99	Staph. aureus
100	Staph. aureus

101	Staph. aureus
102	Staph. aureus
103	Staph. equorum
104	Staph. agnetis
105	Staph. aureus
106	Staph. aureus
107	Staph. aureus
108	Staph. aureus
109	Staph. aureus
110	Staph. aureus
111	Staph. aureus
112	Staph. aureus
113	Staph. aureus
114	Staph. aureus
115	Staph. aureus
116	Staph. schleiferi
117	Staph. aureus
118	Staph. aureus
119	Staph. aureus
120	Staph. aureus
121	Staph. saprophyticus
122	Staph. saprophyticus
123	Staph. lugdunensis
124	Staph. aureus
125	Staph. lugdunensis
126	Staph. simulans
127	Staph. aureus
128	Staph. aureus
129	Staph. aureus
130	Staph. aureus
131	Staph. aureus
132	Staph. aureus
133	Staph. aureus
134	Staph. aureus

135	Staph. aureus
136	Staph. haemolyticus
137	Staph. aureus
138	Staph. condimenti
139	Staph. aureus
140	Staph. aureus
141	Staph. aureus
142	Staph. aureus
143	Staph. aureus
144	Staph. aureus
145	Staph. aureus
146	Staph. sp.
147	Staph. equorum
148	Staph. pseudintermedius
149	Staph. equorum
150	Staph. pseudintermedius
151	Staph. carnosus
152	Staph. aureus
153	Staph. aureus
154	Staph. aureus
155	Staph. aureus
156	Staph. aureus
157	Staph. condimenti
158	Staph. aureus
159	Staph. aureus
160	Staph. aureus
161	Staph. aureus
162	Staph. aureus
163	Staph. cohnii
164	Staph. aureus
165	Staph. aureus
166	Staph. aureus
167	Staph. aureus

168	Staph. aureus
169	Staph. aureus
170	Staph. aureus
171	Staph. aureus
172	Staph. aureus
173	Staph. aureus
174	Staph. aureus
175	Staph. aureus
176	Staph. aureus
177	Staph. aureus
178	Staph. aureus
179	Staph. aureus
180	Staph. sciuri
181	Staph. lugdunensis
182	Staph. xylosus
183	Staph. aureus
184	Staph. epidermidis
185	Staph. aureus
186	Staph. aureus
187	Staph. aureus
188	Staph. aureus
189	Staph. aureus
190	Staph. aureus
191	Staph. aureus
192	Staph. lutrae
193	Staph. aureus
194	Staph. aureus
195	Staph. aureus
196	Staph. pettenkoferi
197	Staph. saprophyticus
198	Staph. sciuri
199	Staph. saprophyticus
200	Staph. aureus
201	Staph. aureus

202	Staph. epidermidis
203	Staph. lugdunensis
204	Staph. aureus
205	Staph. aureus
206	Staph. capitis
207	Staph. aureus
208	Staph. simulans
209	Staph. aureus
210	Staph. lugdunensis
211	Staph. nepalensis
212	Staph. pasteuri
213	Staph. nepalensis
214	Staph. aureus
215	Staph. capitis
216	Staph. lugdunensis
217	Staph. aureus
218	Staph. aureus
219	Staph. aureus
220	Staph. aureus
221	Staph. aureus
222	Staph. aureus
223	Staph. aureus
224	Staph. aureus
225	Staph. aureus
226	Staph. epidermidis
227	Staph. hominis
228	Staph. aureus
229	Staph. aureus
230	Staph. aureus
231	Staph. aureus
232	Staph. piscifermentans
233	Staph. muscae
234	Staph. simiae
235	Strep. pyogenes

236	Strep. pneumoniae
237	Strep. pneumoniae
238	Strep. agalactiae
239	Strep. pyogenes
240	Strep. pyogenes
241	Strep. mutans
242	Strep. dysgalactiae
243	Strep. pyogenes
244	Strep. pyogenes
245	Strep. pyogenes
246	Strep. thermophilus
247	Strep. thermophilus
248	Strep. pyogenes
249	Strep. agalactiae
250	Strep. pyogenes
251	Strep. pyogenes
252	Strep. pyogenes
253	Strep. pyogenes
254	Strep. sanguinis
255	Strep. suis
256	Strep. suis
257	Strep. pneumoniae
258	Strep. thermophilus
259	Strep. gordonii
260	Strep. pyogenes
261	Strep. suis
262	Strep. pneumoniae
263	Strep. pneumoniae
264	Strep. pneumoniae
265	Strep. pneumoniae
266	Strep. pneumoniae
267	Strep. pneumoniae
268	Strep. pneumoniae
269	Strep. equi

270	Strep. equi
271	Strep. pneumoniae
272	Strep. mitis
273	Strep. gallolyticus
274	Strep. mutans
275	Strep. pneumoniae
276	Strep. pneumoniae
277	Strep. parasanguinis
278	Strep. suis
279	Strep. pneumoniae
280	Strep. suis
281	Strep. dysgalactiae
282	Strep. pneumoniae
283	Strep. suis
284	Strep. pneumoniae
285	Strep. pneumoniae
286	Strep. pneumoniae
287	Strep. pneumoniae
288	Strep. pneumoniae
289	Strep. pneumoniae
290	Strep. pneumoniae
291	Strep. parauberis
292	Strep. equi
293	Strep. pseudopneumoniae
294	Strep. pyogenes
295	Strep. suis
296	Strep. suis
297	Strep. suis
298	Strep. suis
299	Strep. suis
300	Strep. pyogenes
301	Strep. pyogenes
302	Strep. pneumoniae
303	Strep. oralis

304	Strep. salivarius
305	Strep. salivarius
306	Strep. parasanguinis
307	Strep. thermophilus
308	Strep. gallolyticus
309	Strep. pasteurianus
310	Strep. mutans
311	Strep. macedonicus
312	Strep. mutans
313	Strep. suis
314	Strep. pneumoniae
315	Strep. agalactiae
316	Strep. agalactiae
317	Strep. intermedius
318	Strep. dysgalactiae
319	Strep. pyogenes
320	Strep. suis
321	Strep. pyogenes
322	Strep. cristatus
323	Strep. suis
324	Strep. iniae
325	Strep. pyogenes
326	Strep. agalactiae
327	Strep. agalactiae
328	Strep. agalactiae
329	Strep. lutetiensis
330	Strep. salivarius
331	Strep. intermedius
332	Strep. intermedius
333	Strep. constellatus
334	Strep. constellatus
335	Strep. constellatus
336	Strep. anginosus
337	Strep. anginosus

338	Strep. suis
339	Strep. anginosus
340	Strep. sp.
341	Strep. sp.
342	Strep. dysgalactiae
343	Strep. suis
344	Strep. agalactiae
345	Strep. agalactiae
346	Strep. iniae
347	Strep. iniae
348	Strep. sp.
349	Strep. agalactiae
350	Strep. equi
351	Strep. thermophilus
352	Strep. agalactiae
353	Strep. agalactiae
354	Strep. suis
355	Strep. pyogenes
356	Strep. pyogenes
357	Strep. pyogenes
358	Strep. pyogenes
359	Strep. pyogenes
360	Strep. pyogenes
361	Strep. pyogenes
362	Strep. agalactiae
363	Strep. salivarius
364	Strep. pneumoniae
365	Strep. mutans
366	Strep. agalactiae
367	Strep. agalactiae
368	Strep. agalactiae
369	Strep. anginosus
370	Strep. iniae
371	Strep. agalactiae

372	Strep. gordonii
373	Strep. thermophilus
374	Strep. suis
375	Strep. pyogenes
376	Strep. thermophilus
377	Strep. pyogenes
378	Strep. pyogenes
379	Strep. pyogenes
380	Strep. pyogenes
381	Strep. pyogenes
382	Strep. pyogenes
383	Strep. pyogenes
384	Strep. pyogenes
385	Strep. agalactiae
386	Strep. pyogenes
387	Strep. pyogenes
388	Strep. agalactiae
389	Strep. agalactiae
390	Strep. agalactiae
391	Strep. pneumoniae
392	Strep. agalactiae
393	Strep. pyogenes
394	Strep. pyogenes
395	Strep. suis
396	Strep. agalactiae
397	Strep. thermophilus
398	Strep. mitis
399	Strep. gordonii
400	Strep. intermedius
401	Strep. anginosus
402	Strep. agalactiae
403	Strep. pneumoniae
404	Strep. gallolyticus
405	Strep. infantarius

406	Strep. thermophilus
407	Strep. pyogenes
408	Strep. pyogenes
409	Strep. salivarius
410	Strep. pyogenes
411	Strep. pyogenes
412	Strep. pyogenes
413	Strep. agalactiae
414	Strep. sp.
415	Strep. mutans
416	Strep. pyogenes
417	Strep. mitis
418	Strep. suis
419	Strep. sp.
420	Strep. agalactiae
421	Strep. agalactiae
422	Strep. suis
423	Strep. pyogenes
424	Strep. pyogenes
425	Strep. pyogenes
426	Strep. pyogenes
427	Strep. pyogenes
428	Strep. suis
429	Strep. pantholopis
430	Strep. agalactiae
431	Strep. thermophilus
432	Strep. pyogenes
433	Strep. agalactiae
434	Strep. thermophilus
435	Strep. anginosus
436	Strep. thermophilus
437	Strep. agalactiae
438	Strep. suis
439	Strep. agalactiae

440	Strep. thermophilus
441	Strep. iniae
442	Strep. pneumoniae
443	Strep. pneumoniae
444	Strep. pneumoniae
445	Strep. agalactiae
446	Strep. agalactiae
447	Strep. suis
448	Strep. oralis
449	Strep. gallolyticus
450	Strep. thermophilus
451	Strep. agalactiae
452	Strep. agalactiae
453	Strep. pyogenes
454	Strep. intermedius
455	Strep. gordonii
456	Strep. equinus
457	Strep. agalactiae
458	Strep. sp.
459	Strep. sp.
460	Strep. salivarius
461	Strep. salivarius
462	Strep. salivarius
463	Strep. suis
464	Strep. pyogenes
465	Strep. suis
466	Strep. agalactiae
467	Strep. agalactiae
468	Strep. agalactiae
469	Strep. agalactiae
470	Strep. agalactiae
471	Strep. agalactiae
472	Strep. agalactiae
473	Strep. agalactiae

474	Strep. agalactiae
475	Strep. equi
476	Strep. agalactiae
477	Strep. iniae
478	Strep. pyogenes
479	Strep. pyogenes
480	Strep. pyogenes
481	Strep. pyogenes
482	Strep. thermophilus
483	Strep. agalactiae
484	Strep. pyogenes
485	Strep. troglodytae
486	Strep. pyogenes
487	Strep. sp.
488	Strep. intermedius
489	Strep. oralis
490	Strep. pneumoniae
491	Strep. gordonii
492	Strep. oralis
493	Strep. thermophilus
494	Strep. pyogenes
495	Strep. suis
496	Strep. suis
497	Strep. iniae
498	Strep. suis
499	Strep. suis
500	Strep. pneumoniae
501	Strep. pneumoniae
502	Strep. uberis
503	Strep. suis
504	Strep. thermophilus
505	Strep. pneumoniae
506	Strep. pyogenes
507	Strep. agalactiae

508	Strep. agalactiae
509	Strep. agalactiae
510	Strep. agalactiae
511	Strep. agalactiae
512	Strep. agalactiae
513	Strep. agalactiae
514	Strep. agalactiae
515	Strep. agalactiae
516	Strep. agalactiae
517	Strep. agalactiae
518	Strep. agalactiae
519	Strep. agalactiae
520	Strep. agalactiae
521	Strep. agalactiae
522	Strep. agalactiae
523	Strep. agalactiae
524	Strep. agalactiae
525	Strep. agalactiae
526	Strep. agalactiae
527	Strep. agalactiae
528	Strep. agalactiae
529	Strep. agalactiae
530	Strep. agalactiae
531	Strep. agalactiae
532	Strep. agalactiae
533	Strep. agalactiae
534	Strep. agalactiae
535	Strep. agalactiae
536	Strep. agalactiae
537	Strep. agalactiae
538	Strep. agalactiae
539	Strep. agalactiae
540	Strep. agalactiae
541	Strep. agalactiae

542	Strep. agalactiae
543	Strep. agalactiae
544	Strep. agalactiae
545	Strep. agalactiae
546	Strep. thermophilus
547	Strep. agalactiae
548	Strep. merionis
